# Cross-Species Functionality of Pararetroviral Elements Driving Ribosome Shunting

**DOI:** 10.1371/journal.pone.0001650

**Published:** 2008-02-20

**Authors:** Mikhail M. Pooggin, Johannes Fütterer, Thomas Hohn

**Affiliations:** 1 Institute of Botany, University of Basel, Basel, Switzerland; 2 Institute of Plant Sciences, Eidgenössische Technische Hochschule Zürich (ETH Zentrum), Zürich, Switzerland; Institut Pasteur Korea, Republic of Korea

## Abstract

**Background:**

*Cauliflower mosaic virus* (CaMV) and *Rice tungro bacilliform virus* (RTBV) belong to distinct genera of pararetroviruses infecting dicot and monocot plants, respectively. In both viruses, polycistronic translation of pregenomic (pg) RNA is initiated by shunting ribosomes that bypass a large region of the pgRNA leader with several short (s)ORFs and a stable stem-loop structure. The shunt requires translation of a 5′-proximal sORF terminating near the stem. In CaMV, mutations knocking out this sORF nearly abolish shunting and virus viability.

**Methodology/Principal Findings:**

Here we show that two distant regions of the CaMV leader that form a minimal shunt configuration comprising the sORF, a bottom part of the stem, and a shunt landing sequence can be replaced by heterologous sequences that form a structurally similar configuration in RTBV without any dramatic effect on shunt-mediated translation and CaMV infectivity. The CaMV-RTBV chimeric leader sequence was largely stable over five viral passages in turnip plants: a few alterations that did eventually occur in the virus progenies are indicative of fine tuning of the chimeric sequence during adaptation to a new host.

**Conclusions/Significance:**

Our findings demonstrate cross-species functionality of pararetroviral *cis*-elements driving ribosome shunting and evolutionary conservation of the shunt mechanism.

We are grateful to Matthias Müller and Sandra Pauli for technical assistance. This work was initiated at Friedrich Miescher Institute (Basel, Switzerland). We thank Prof. Thomas Boller for hosting the group at the Institute of Botany.

## Introduction

Ribosome shunt is a mechanism of eukaryotic translation initiation that combines features of both 5′-end dependent scanning and internal ribosome entry. It has been discovered in plants, first for *Cauliflower mosaic virus* (CaMV) [1], [2] and then for *Rice tungro bacilliform virus* (RTBV) [3]. Related phenomena have also been reported for several viral and cellular mRNAs in animal, yeast and green alga cells [14], [5], [6], [7], [8], [9]; [reviewed in Refs. 10]; [11].

**Figure 1:**
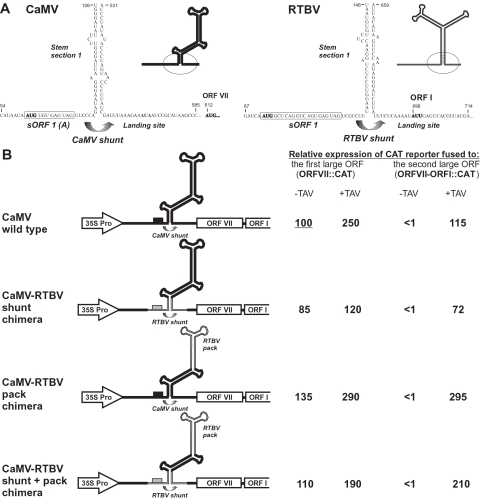
Conserved shunt configuration from the RTBV leader imbedded in the CaMV leader drives efficient polycistronic expression in plant protoplasts. (A) The large stem-loop structures of the leaders predicted by MFold for CaMV (left) and RTBV (right) and experimentally verified for CaMV [19] are schematically drawn with thick lines. The 5′- and 3′-sequences flanking the main structure are shown in open conformation. The stable structural element at the stem base (stem section 1) and adjacent regions, are enlarged and their sequences shown (these sequences substitute one another in the CaMV-RTBV chimeric leader and virus). The nucleotide numbering is from the pgRNA 5′-end (cap-site). The 5′-proximal short ORF (sORF 1) is boxed. The AUG start codons and the non-AUG initiating codons in the shunt landing site are in bold. (B) Polycistronic expression controlled by the wild type and chimeric CaMV-RTBV leaders (shown on the left) in *O. violaceous* protoplasts. Relative expression levels of the CAT reporter gene fused to the first (ORFVII::CAT) or the second (ORFVII-ORFI::CAT) viral ORF downstream of the leader in the absence (−TAV) or the presence (+TAV) of the TAV expressing plasmid are given. Expression of ORFVII::CAT downstream of the wild type CaMV leader in the absence of TAV is set to 100%.

The CaMV shunt mechanism has been extensively studied in plant protoplast and *in vitro* translation systems [12], [1], [2], [13], [14], [15], [16], [17], [18]. According to our current model, shunt-mediated translation initiation on the CaMV pregenomic (pg)RNA includes the following steps: (i) a 40S ribosomal subunit binds the pgRNA capped 5′-end and scans along the leader sequence until a first AUG, the start codon of short ORF 1 (also called sORF A), is encountered; (ii) an 80S ribosome assembles and initiates translation of sORF 1; (iii) the ribosome terminates translation and disassembles at the sORF stop codon, the shunt take-off site, located six nucleotides upstream of the base of a large stem-loop structure with two bifurcations dividing it into stem sections 1, 2, and 3 [19], [15] (Fig. 1A); (iv) the released 40S, retaining initiation factor(s) necessary for scanning and re-initiation but having lost those capable of melting stable structure, shunts over (bypasses) about 480 nt structured region to reach a shunt landing site downstream of the structure, where (v) it resumes scanning and finally re-initiates translation at the start codon of the first large viral ORF (ORF VII). In CaMV, mutations of the start or the stop codon of sORF 1, but not of its coding sequence, nearly abolished shunting and drastically reduced viral infectivity in turnip plants, leading to appearance of first and second site reversions restoring a short ORF [14], [15] These findings indicated that sORF-mediated ribosome shunting is essential for viral infectivity. However, the importance of other *cis*-acting elements found to be essential for ribosome shunting in protoplasts and *in vitro*, namely, the stem section 1 [12], [13], [16] and the shunt landing site [2], [17], was not tested *in planta*.

CaMV and RTBV belong to the *Caulimovirus* and *Tungrovirus* genera of the family *Caulimoviridae*. They replicate via reverse transcription of pgRNA and encapsidate circular double-stranded DNA of ∼8 kbp. Their life cycles differ in many aspects including host range, insect vectors and virion geometry [20], [21]. Furthermore, genome organization (seven genes in CaMV versus four in RTBV) and gene expression strategies are very different. In CaMV, 35S and 19S promoters drive transcription of two major units, pgRNA (35S RNA) and 3′ co-terminal, subgenomic RNA (19S RNA). 19S RNA serves as a monocistronic mRNA for transactivator/viroplasmin (TAV) [22] that transactivates expression of several internal ORFs from the polycistronic 35S RNA (ORFs I and II) and its spliced version (ORFs IV and V) by enabling ribosomes to re-initiate translation [23], [24], [25], [26]; [reviewed in Ref. 27]. In contrast, RTBV does not encode a translational transactivator and its internal ORFs II and III are translated from pgRNA by leaky scanning [28], while ORF IV is translated from a monocistronic, spliced version of pgRNA [29]. In the latter case, an inefficient splicing event fuses the pgRNA leader-based sORF 1 and ORF IV [29]. Notably in CaMV, a fraction of 35S RNA molecules are also spliced and one of the four splice donor sites was mapped to the leader, but far downstream from sORF 1 [29]. This splicing event removes ORFs VII, I and II, thus enabling expression of further downstream ORFs.

Despite these differences, both CaMV [2] and RTBV [3] use shunting to initiate translation of the first large ORF downstream of the respective pgRNA leaders that carry multiple sORFs and form stable stem-loop secondary structures (Fig. 1A). Our recent experiments in rice protoplasts and wheat germ extracts demonstrated that, like in CaMV, the mechanism of RTBV shunting involves translation of sORF 1 terminating near the stem structure and requires integrity of the stem section 1 and the shunt landing site just downstream of the stem [17]. This supported our earlier bioinformatic prediction of the conserved shunt configuration in the pgRNA leaders of CaMV and RTBV (Fig. 1A) as well as other plant pararetroviruses [30], [31]. Here we demonstrate that all the three essential *cis*-elements forming the minimal shunt configuration in CaMV–sORF 1, stem section 1 and the landing site-can be functionally substituted by respective elements from RTBV to confer efficient shunt-mediated polycistronic translation and virus infectivity *in planta*.

## Results and Discussion

### The RTBV shunt elements imbedded in the CaMV leader confer efficient shunting and TAV-activated polycistronic translation in protoplasts derived from a CaMV host plant

Using a PCR ligation method, we replaced two distant regions of the 612 nt CaMV leader sequence (positions 54-106 and 530-585) with corresponding regions of the RTBV leader (positions 87-148 and 659-714) (Fig. 1A). Secondary structure analysis using the Wisconsin GCG MFold program suggested that this replacement did not affect integrity of an upper part of the central stem-loop structure that harbors a putative RNA packaging signal specifically interacting with the CaMV coat protein [32], although the overall stability of the leader secondary structure was predicted to be slightly increased (see Data S1). Other known *cis*-elements located in the CaMV leader, namely, a transcriptional/translational enhancer (positions 1-52) [33], a poly(A) signal with upstream elements (positions 148-177) [34], the 5′-splice site (positions 482-491) [25] and a primer binding site for reverse transcription (positions 599-612), were not affected.

To test the effect of this replacement on leader-controlled translation we used a transient expression system based on plant protoplasts from cell suspension of *Orychophragmus violaceus* (a CaMV host plant), in which the CAT reporter gene is expressed from transiently-transfected plasmid constructs as part of the modified CaMV 35S RNA transcription unit. This system has been established in our previous studies on shunting and transactivation [23], [2], [16]. In the construct ORFVII::CAT, CAT is fused to ORF VII to monitor shunt-mediated expression of the first ORF downstream of the leader. In the construct ORFVII-ORFI::CAT, CAT is fused to ORF I to monitor TAV-mediated expression of the second ORF following ORF VII (Fig. 1B). The relative expression of CAT from these plasmids was examined in the absence and presence of a separate plasmid expressing the CaMV TAV protein (pHELP7) [23]. Additionally, a plasmid expressing GUS as a second reporter gene was always co-transfected to serve as an internal control of transfection efficiency and to normalize CAT levels as described by Pooggin et al [16].

The basal level of shunt-mediated expression from the monocistronic construct ORFVII::CAT was set to 100%. Consistent with previous reports [2], [16], [14], it was enhanced 2.5 times by TAV (Fig. 1B). The relative CAT expression from the dicistronic construct ORFVII-ORFI::CAT was below 1%. In the presence of TAV, it was transactivated up to 115%, again confirming previous results [23]. The CaMV-RTBV shunt chimeras had similar expression profiles, albeit the levels of shunting (85%) and TAV-activated ORF I expression (72%) were slightly lower (Fig. 1B).

Similar results were obtained when, in addition to the shunt elements, the CaMV leader region (positions 230-408) forming stem-section 3 and the bowl structure (a presumed pgRNA packaging signal) [32] was replaced with the corresponding region of the RTBV leader (positions 238-421) designated here “RTBV pack” (Fig. 1B). Interestingly, both in the presence and the absence of TAV, the expression levels of the RTBV pack-containing constructs were higher than those of the respective constructs with the original “CaMV pack” sequence. Previously, we have reported that replacement of the CaMV leader region forming the entire hairpin structure above stem section 1 with a short sequence forming a perfectly double-stranded hairpin also increased shunt-mediated translation [16]. In both cases, higher propensity of the leader sequence to fold into the shunt-supporting configuration, with stem section 1 stabilized by a more stable (and/or compact) structure above, might account for increased efficiency of shunting. According to the current model of CaMV polycistronic translation, the ribosomes having completed translation of ORF VII can reinitiate at ORF I in the presence of TAV [26], [11]. Therefore, an increase in translation of ORF VII should lead to a comparable increase in translation of ORF I. However, this model does not explain why the RTBV pack sequence had a greater contribution to expression of ORF I than to that of ORF VII (Fig 1B). This finding suggests an additional, leader sequence-controlled and TAV-dependent mechanism of ORF I translation, which would bypass the ORF VII start codon.

We conclude that the RTBV *cis*-elements imbedded in the CaMV leader can functionally substitute the corresponding CaMV elements in driving efficient shunt-mediated translation initiation and TAV-activated dicistronic translation.

### The RTBV shunt elements support CaMV infection in turnip plants

To test whether the RTBV shunt configuration can support CaMV infection *in planta*, we introduced the four chimeric leader sequences into the CaMV infectious clone pCa540, a derivative of CM4-184 lacking the insect transmission factor due to a natural deletion in ORF II, and mechanically inoculated turnip seedlings with the resulting DNA as described in [15]. Both chimeric viruses containing the RTBV pack sequence were not infectious, most likely because the presumed RNA packaging signal within this region requires the RTBV coat protein for functionality [32]. In contrast, plants inoculated with the chimeric virus containing the sORF1, the stem-section 1 and the landing site sequences of RTBV developed viral disease symptoms similar to those caused by wild-type CaMV, albeit with a delay of about 10 days.

**Figure 2:**
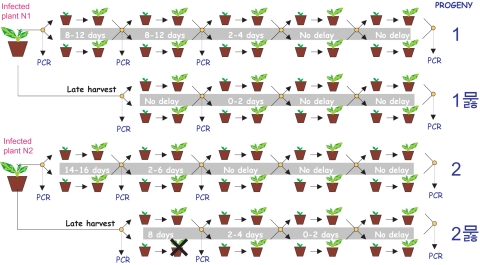
Serial passages of the CaMV-RTBV chimeric virus in turnip plants. The experimental scheme of serial passages for the early harvest (1 and 2) and the late harvest (1′ and 2′) progenies stemming from two initially infected plants (N1 and N2) is depicted. For each progeny, a delay (in days) in symptom development for the chimeric virus versus the wild type virus is given. Samples taken for PCR amplification and sequencing of viral DNA are indicated by circles (for the detailed sequencing data, see Data S1).

The increased latency period indicated that the chimeric leader sequence is not fully optimal for one or more processes of the viral replication cycle. We have shown previously that suboptimal viral genomes restore or adjust features important for optimized infectivity and fitness by first or second site reversions [14], [15]. The nature of selected revertant genomes might allow conclusions about the underlying mechanisms affected by the original mutations. Therefore, we performed several passages of the viral progenies to new turnip plants using sap-inoculation and, after each passage, monitored virus latency periods (Fig. 2). Alterations in the chimeric leader sequence were examined in samples from young, systemically infected leaves, which had been used for sap-inoculation, by PCR amplification of a 834 bp fragment of viral DNA containing the complete leader with flanking 35S promoter and ORF VII sequences, followed by cloning of the PCR product and sequencing of several individual clones per progeny as described in detail previously [15].

For each of the two plants initially infected with the chimeric virus, samples of young leaves were harvested at about 2 and 3 months post-inoculation, and two parallel series of passages were performed (five passages for the early harvest and four for the late harvest). The early harvest progenies were designated 1 and 2, the late harvest ones 1′ and 2′ (Fig. 2). All the sequence alterations detected in each viral progeny in the course of passages are provided in Data S1.

**Figure 3:**
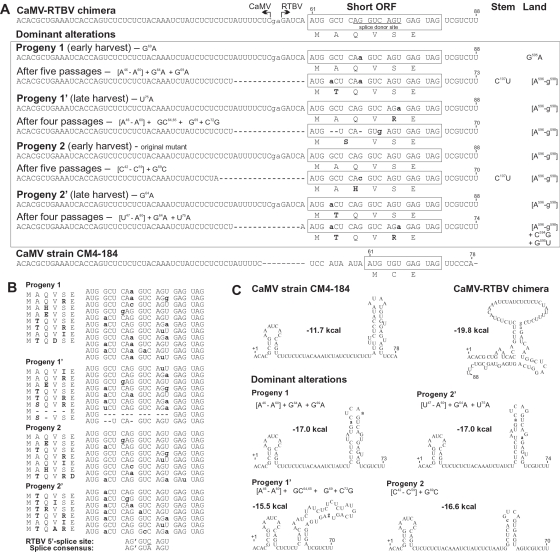
Alterations in the sORF1 region of the CaMV-RTBV chimeric virus. (A) The nucleotide sequences of the original CaMV-RTBV chimeric leader (top), the wild type CaMV strains CM4-184 and Cabb-S (bottom) and the dominant revertants obtained in the four progenies initially and after several passages (middle panel) are shown. The junction between CaMV and RTBV sequences is indicated by bent arrows (two non-viral nucleotides are in lower case). For each sequence, sORF is boxed and the encoded peptide indicated (altered amino acids are in bold). Nucleotide substitutions in the progeny viruses are shown in bold, low case. The middle panel also shows alterations in stem section 1 (Stem) and the shunt landing site (Land) regions dominating in the respective progenies. The nucleotide numbering is from the pgRNA 5′-end. (B) A complete collection of the sORF1 peptide and nucleotide sequence variants found in the sequenced clones for each progeny, both initially and on passage (for details, see Data S1). The RTBV splice donor and the consensus spliced donor sequences are aligned below. (C) The MFold-predicted local secondary structure for the wild type, the chimeric and the dominant progeny leader sequences preceding the central stem structure are depicted. The stability of each structure in kcal per mole is indicated.

**Figure 4:**
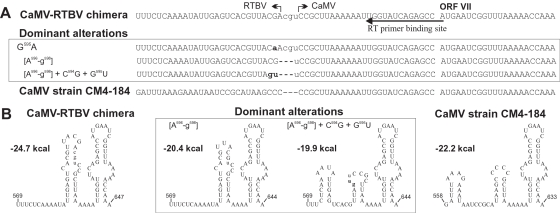
Alterations near the shunt landing region of the CaMV-RTBV chimeric virus. (A) The nucleotide sequences of the original CaMV-RTBV chimeric leader (top), the wild type CaMV strain CM4-184 (bottom) and the revertants obtained in the four progenies (middle panel) are shown. The junction between CaMV and RTBV sequences is indicated by bent arrows (three non-viral nucleotides are in lower case). Nucleotide substitutions in the progeny viruses are shown in bold, low case. (B) The MFold-predicted local secondary structure for the wild type (right), the chimeric (left) and the dominant progeny leader sequences (middle) located downstream of the central stem-loop structure are depicted. The stability of each structure in kcal per mole is indicated.

For each progeny, the wild-type latency period was completely restored after one to three passages (Fig. 2). This restoration correlated well with the appearance of some predominant alterations in the chimeric leader sequence (summarized in Figs. 3 and 4; for more details of each passage, see Data S1). In contrast, sequencing of the ∼1.6 kbp TAV coding region of several clones representing each progeny of the chimeric virus after the final passages did not reveal any sequence alteration. Changes in the chimeric leader occurring several times independently and changes that are fixed in subsequent generations therefore most likely have a functional importance.

Our analysis of the virus genomes at early stages of propagation revealed a large collection of individual clones with and without reversions. The recurring feature was one nucleotide substitution inside the RTBV sORF (G64A, G69A, or, less frequently, C67G, G69C, G74U and U75G) combined either with a substitution G595A or a deletion of three adjacent nucleotide (Δ596-598) at the junction between the RTBV landing site and the downstream CaMV sequence (Fig. 3A and 4A; and Data S1). Interestingly, analysis of the late harvest progenies showed that the chimeric virus continued to evolve within a single, initially infected plant (Fig. 3A and Data S1). In fact, these additional alterations appeared to improve viral fitness, because the late harvest progenies had shorter latency periods than the early harvest ones (Fig. 2). Notably, most of the dominant alterations (e.g. G64A and Δ596-598) occurred independently in progenies stemming from the two initially infected plants, indicating their importance for viral fitness.

For the initial heterogeneous populations, it is unclear which of the clones actually support infection and how much of the present variation is covered by the sequenced clones. However, upon serial passage, a purifying selection should occur and analysis of the resulting populations should become more informative.

### Analysis of the viral progenies after several passages

Upon several passages, symptom development was no longer retarded (Fig. 2) and the population isolated from a given plant turned out to be more uniform (see Data S1). By comparing the four progenies, a small number of recurring alterations in the chimeric leader sequences could be discerned. All 43 analyzed clones showed alterations of the sORF1 coding sequence and all clones also showed changes at the 3′ border of the RTBV landing site or the junction sequence. In the sORF of three progenies, the second codon was altered such that the initiation context was weakened and a Ser or Thr was encoded instead of an Ala (Fig. 3A and 3B). In all but one progenies, the third codon containing the exonic part of the RTBV splice donor site was altered (Fig. 3A) in a way predicted to reduce the splicing efficiency. Surprisingly, the GU of the intronic part of the splice site, which represents the most conserved signal [35], was altered only in one of the late progeny clones and also only in two clones of the intermediate populations (Fig. 3B). The fourth or fifth codon was altered in two of the four progenies, while the sixth codon remained unchanged in all the progenies.

Mutations at the junction of the RTBV landing site to the CaMV downstream region affected the two last nucleotides in the RTBV sequence or the artificial junction itself (Fig. 4A). The G595A mutation was frequent already in the first generation progenies, while, in all the late progenies, small deletions in this region were found, with Δ596-598 being predominant (Fig. 4A and Data S1). Strikingly, also the 5′ junction between CaMV leader and RTBV sORF1 gave rise to deletions or mutations in most cases (Fig. 3A) and smaller deletions close by in some (see Data S1). Almost all these changes affected the palindromic restriction sites engineered at the junctions. Besides these common alterations, a C107U or C109U mutation appeared independently in two virus progenies. These bases are located in a three-nucleotide bulge of stem section 1 and might be a protein-binding site or be involved in long-range RNA interactions.

The pgRNA leader sequence of plant pararetroviruses is involved in many different processes that could potentially be affected by reversions. Reversions affecting the RTBV splice donor in sORF1 should lead to reduced production of aberrant splicing products from the RTBV splice site to the authentic CaMV splice acceptor site [25]. In one late progeny population, the most conserved nucleotides of the splice site remained unchanged but the viral latency period was no longer compromised. In this case, mIn this utation in the fifth codon of sORF 1 (U75A) may also affect splicing by weakening interactions with the U1 and U6 snRNAs [35]. Furthermore, we have previously found that mutation of the normal splice site leads to the usage of an alternative splice donor including the GU of this fifth codon [29]. Besides splicing, sORF1 mutations might influence translation initiation or termination events at this ORF and thus might be involved in fine tuning of the ribosome fate for shunting and reinitiation. From our previous work, we conclude that many different coding sequences can support the sORF1 function in controlling translation, however, clearly not all codon combinations work equally well and some are inhibitory [14], [16], [18]. We mobilized some of the “revertant” leader sequences into our gene expression constructs and tested them for effects on ORF VII translation and found no or only marginal improvements of expression (data not shown). Since the ORF VII::CAT reporter construct lacks any splice acceptor, the latter results suggest that the reversions in the sORF might not primarily occur to compensate for a slight decrease in shunt-mediated translation (Fig. 1B) and could therefore be more important for inactivation of the RTBV splice donor that would interfere with proper splicing in the context of viral pgRNA.

All changes of the RNA sequences lead to subtle alterations of the predicted secondary structures and–as a rule–the observed reversions reduce stability and restore shape of the local secondary structures (Fig. 3C and 4B; and for all the revertant structures, see Data S1). Deletions at the 3′ junction occur in the region where reverse transcription of pregenomic RNA is initiated. The small insertions and substitutions frequently observed in a nearby region in front of the primer binding site [15] (also, see Data S1) suggest that this initiation and early elongation process may be particularly error prone, possibly because the association between reverse transcriptase and pgRNA is not yet stable. In such a situation, elongation might be particularly affected by RNA secondary structure. The observed alterations all reduce the number of ACGU palindromes which might be involved in structure formation within the RNA or with the second RNA molecule that most likely is involved in complete reverse transcription of the genome.

### Concluding remarks

Our study demonstrates that *cis*-elements driving ribosome shunting are functionally conserved between monocot and dicot pararetroviruses. Indeed, two distant regions of CaMV pgRNA leader–the shunt take-off and the landing sites brought into a close spatial vicinity by formation of stem section 1-can be substituted by the corresponding regions from RTBV in driving efficient shunt-mediated polycistronic translation in CaMV host plant protoplasts and in supporting CaMV infectivity in turnip plants. Our findings indicate that primary sequences of sORF 1, stem-section 1 and landing site are not absolutely essential for ribosome shunting and viral infectivity, unless they carry some inhibitory features or alter low index of local secondary structure upstream and downstream of the central stem-loop structure. Our previous work demonstrated that a regulatory sORF such as the *AdoMetDC* sORF MAGDIS [36] or the *GCN4* sORF 4 [37] that can conditionally block downstream translation reinitiation, when introduced in place of CaMV sORF1, inhibits shunt-mediated translation downstream of the CaMV leader [16], [18]. We therefore assume that some of the dominant alterations in the coding content of RTBV sORF 1 that occurred on passage of the chimeric virus in turnip plants might have slightly modulated elongation or termination rates of sORF 1 translation that controls shunt-mediated polycistonic translation on viral pgRNA. In addition, heterologous sequences may carry elements that affect proper processing (capping, splicing and polyadenylation) or decay of RNA. Indeed, the 5′-splice site located within the RTBV sORF 1 sequence was affected by most (but not all) nucleotide substitution dominating in progenies of the CaMV-RTBV chimera. Low propensity to formation of secondary structure appears to be a main characteristic of the shunt landing site in both CaMV and RTBV, although some unknown features of its primary sequence do contribute to shunt efficiency in host and non-host translation systems [17]. Consistent with the fact that the RTBV shunt landing site, individually or in combinations with other *cis*-elements comprising the minimal shunt configuration, can function efficiently in CaMV host plant protoplasts [17; this study], it does so in the context of CaMV infection in turnip plants as well. The only dominant alteration that occurred in vicinity of the RTBV landing site was the deletion of three nucleotides at the junction with downstream CaMV sequence. Our MFold-assisted analysis shows that the latter deletion restores both low index and shape of local secondary structure. Likewise, a main purpose of the dominant deletions in the chimeric sequence upstream of sORF 1 appears to be relaxation of local secondary structure. We assume that, in both cases, the ribosome scanning process should be facilitated. This assumption is supported by earlier findings that CaMV shunting does not operate, when a stable secondary structure element that blocks scanning is inserted just upstream of sORF 1 or downstream of the landing site in the CaMV leader [2].

It has been proposed that the ascending and descending arms of the CaMV leader stem section 1 have evolved through head-to-head incorporation of long terminal repeats of an ancient retrotransposon found in the yeast genome [38]. However, the primary sequences building the stem section 1 in RTBV (Fig. 1) do not appear to bear any significant homology to the yeast retrotransposon sequence. Moreover, our studies show that primary sequences of stem section 1 are not essential for shunt-mediated translation [12], [16], [17] and CaMV infectivity [this study]. Thus, the conserved, minimal shunt configurations identified in CaMV and RTBV as well as in other plant pararetroviruses [30] may have evolved independently.

Moissiard and Voinnet [39] have reported that the central hairpin of the CaMV leader codes for small interfering RNAs, the effector molecules of RNA silencing, that target certain host transcripts for cleavage and degradation in a sequence-specific manner. In particular, two such siRNAs are derived from the ascending and descending arms of stem section 1. Our study shows that heterologous RTBV sequences, which do not bear any similarity to the predicted siRNAs, can substitute for the CaMV sequences involved in formation of stem-section 1 without any notable effect on CaMV infectivity and these sequences are stable over several passages. Furthermore, our previous studies have shown that second site reversions accumulating in progenies of different CaMV mutants compensated for defects in secondary structure rather than primary sequences of the leader stem section 3 [14], [15] to which three other predicted siRNAs map [39]. Taken together, our findings indicate that siRNAs derived from different regions of the CaMV leader including stem section 1 [40; and our unpublished data] may not have any substantial deliberate function in sequence-specific inactivation of certain host genes.

## Materials and Methods

### Protoplast preparation and transfection

Protoplasts were prepared and transfected with plasmid DNA as described previously [16]. Briefly, 2×10^6^ protoplasts were transfected with 10 µg CAT-expressing plasmid and 2 µl GUS-expressing plasmid. The latter served as an internal control of transfection efficiency. For transactivation, 5 µg TAV-expressing plasmid was also added. Following incubation for 19–24 hrs at 27°C in the dark, protoplasts were harvested, protein extracts prepared and assayed for CAT and GUS accumulation. Relative GUS activities were taken for normalization of CAT expression levels. For each construct, the values given are the means of at least three experiments in independent batches of protoplasts. Deviations from the mean values did not exceed 20%.

### Virus and plants

Construction of CaMV mutants, mechanical inoculation of turnip plants, DNA preparation and PCR, cloning and sequencing of viral progeny from infected plants were performed as described in detail in [14], [15].

## Supporting Information

Data S1 (1.57 MB PPT)
