# Supplementary material for: Cross-Species Functionality of Pararetroviral Elements Driving Ribosome Shunting
Source: PLoS One. 2008 Feb 20;3(2):e1650. doi: 10.1371/journal.pone.0001650 (PMC2241666; doi:10.1371/journal.pone.0001650)
Supplement: Data S1 — (1.57 MB PPT) [file pone.0001650.s001.ppt]

## Slide 1
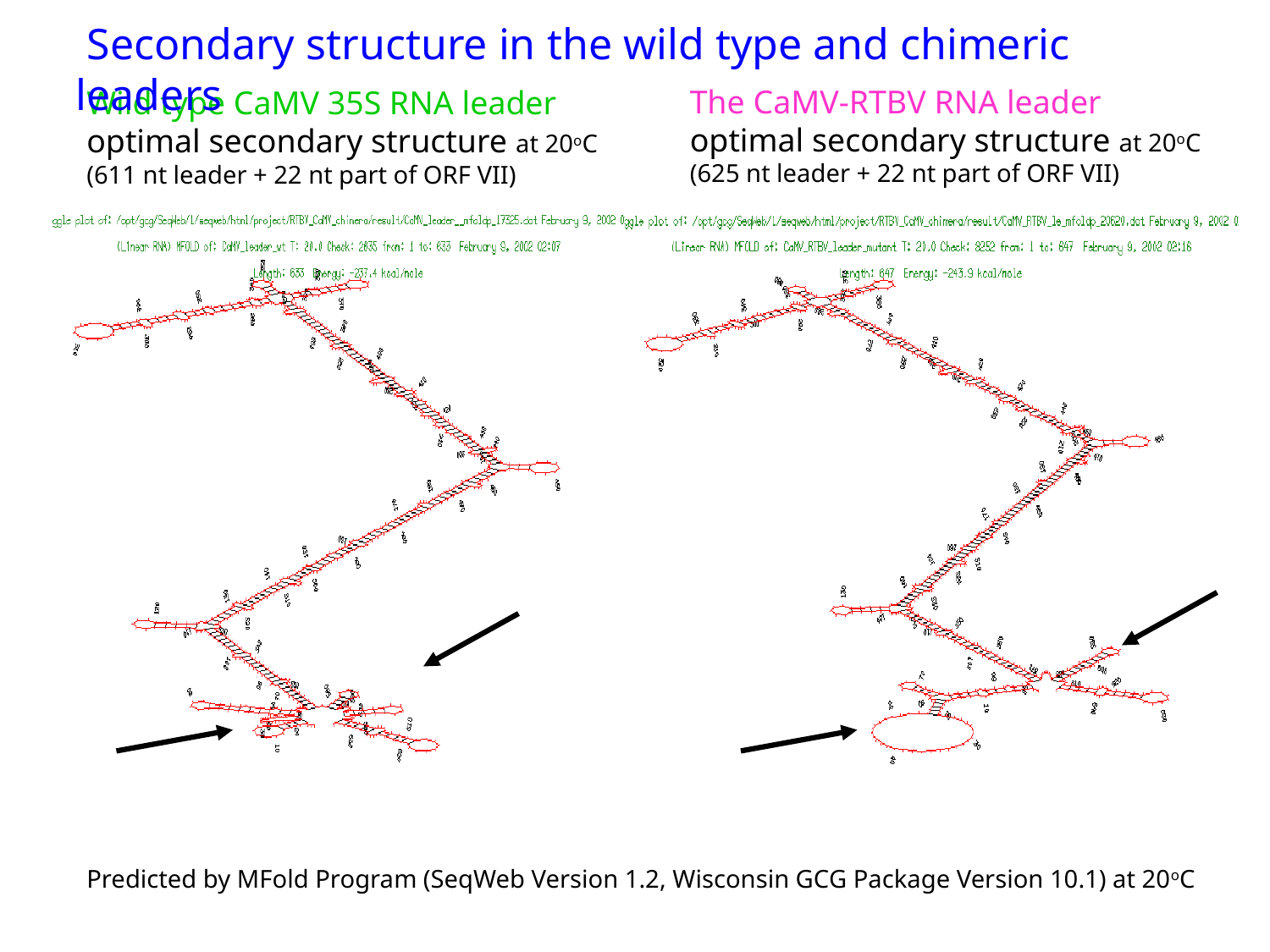

Secondary structure in the wild type and chimeric leaders
The CaMV-RTBV RNA leader optimal secondary structure at 20oC
(625 nt leader + 22 nt part of ORF VII)
Wild type CaMV 35S RNA leader optimal secondary structure at 20oC
(611 nt leader + 22 nt part of ORF VII)
Predicted by MFold Program (SeqWeb Version 1.2, Wisconsin GCG Package Version 10.1) at 20oC

## Slide 2
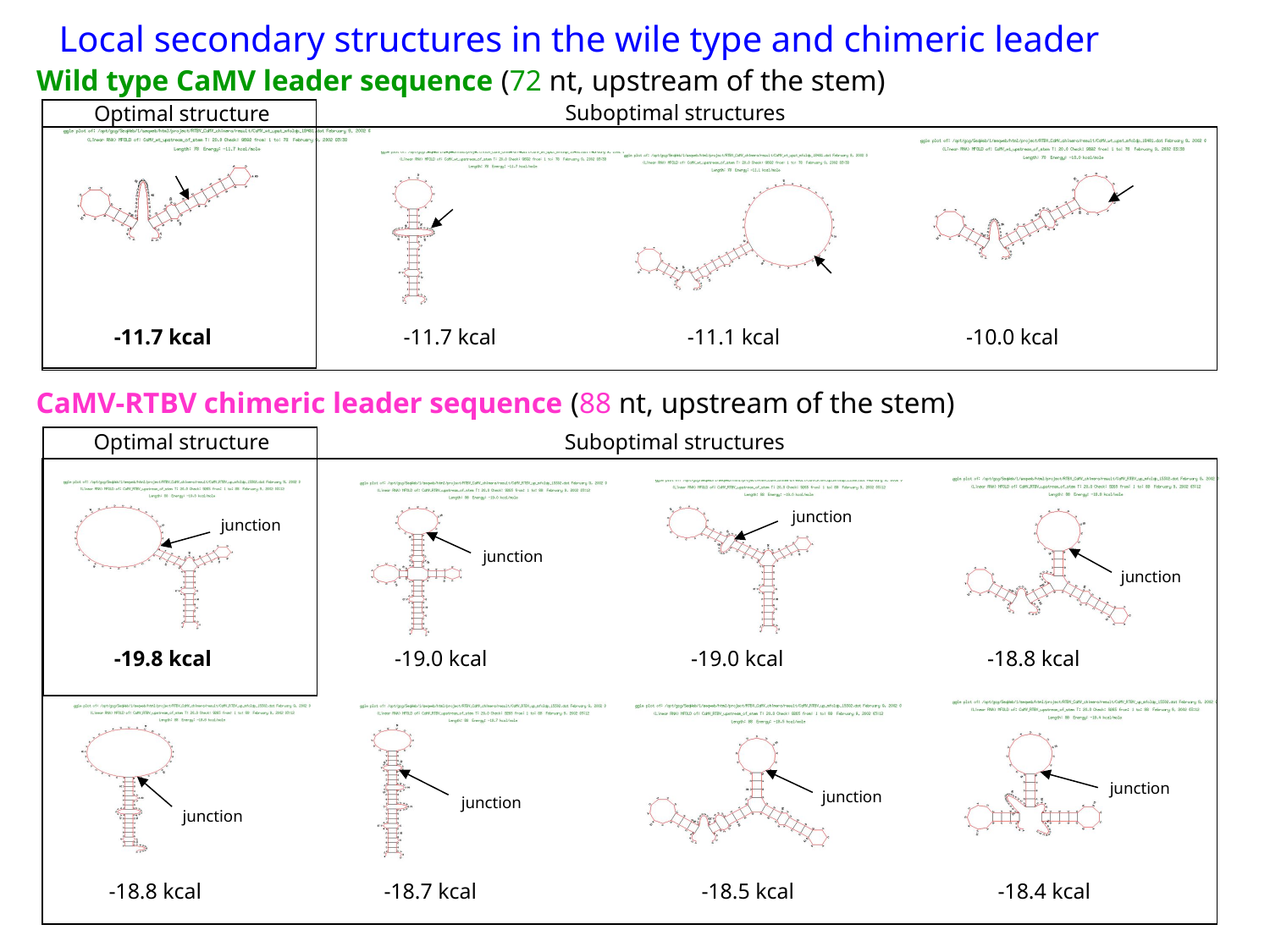

Local secondary structures in the wile type and chimeric leader
Wild type CaMV leader sequence (72 nt, upstream of the stem)
Suboptimal structures
Optimal structure
-11.7 kcal
-11.7 kcal
-11.1 kcal
-10.0 kcal
CaMV-RTBV chimeric leader sequence (88 nt, upstream of the stem)
Suboptimal structures
Optimal structure
junction
junction
junction
junction
-19.8 kcal
-19.0 kcal
-19.0 kcal
-18.8 kcal
junction
junction
junction
junction
-18.8 kcal
-18.7 kcal
-18.5 kcal
-18.4 kcal

## Slide 3
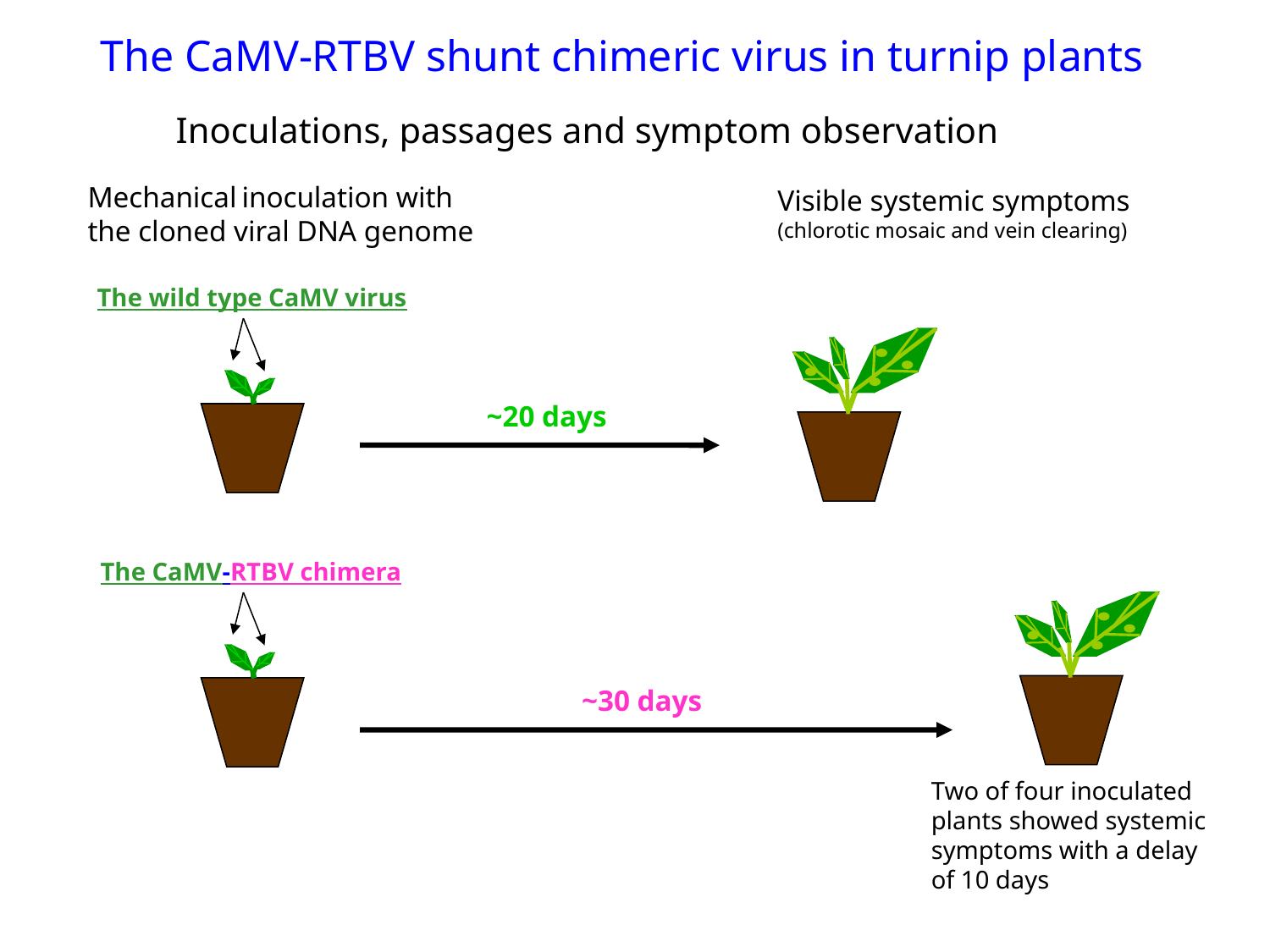

The CaMV-RTBV shunt chimeric virus in turnip plants
Inoculations, passages and symptom observation
Mechanical inoculation with
the cloned viral DNA genome
Visible systemic symptoms
(chlorotic mosaic and vein clearing)
The wild type CaMV virus
~20 days
The CaMV-RTBV chimera
~30 days
Two of four inoculated
plants showed systemic
symptoms with a delay
of 10 days

## Slide 4
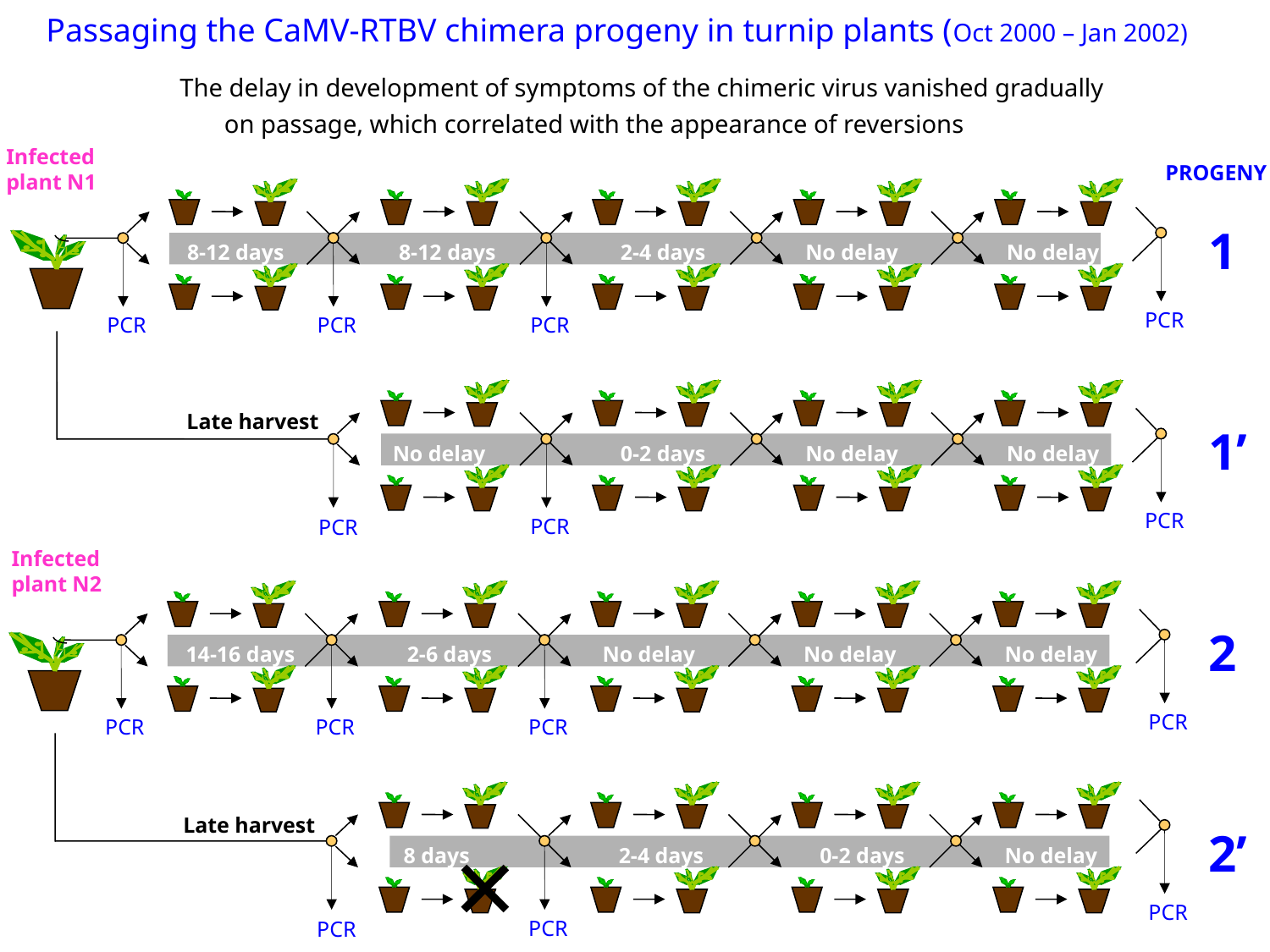

Passaging the CaMV-RTBV chimera progeny in turnip plants (Oct 2000 – Jan 2002)
The delay in development of symptoms of the chimeric virus vanished gradually
 on passage, which correlated with the appearance of reversions
Infected
plant N1
PROGENY
PCR
PCR
PCR
1
8-12 days
8-12 days
2-4 days
No delay
No delay
PCR
Late harvest
PCR
PCR
1’
No delay
0-2 days
No delay
No delay
PCR
Infected
plant N2
PCR
PCR
PCR
2
14-16 days
2-6 days
No delay
No delay
No delay
PCR
PCR
Late harvest
2’
8 days
2-4 days
0-2 days
No delay
PCR
PCR

## Slide 5
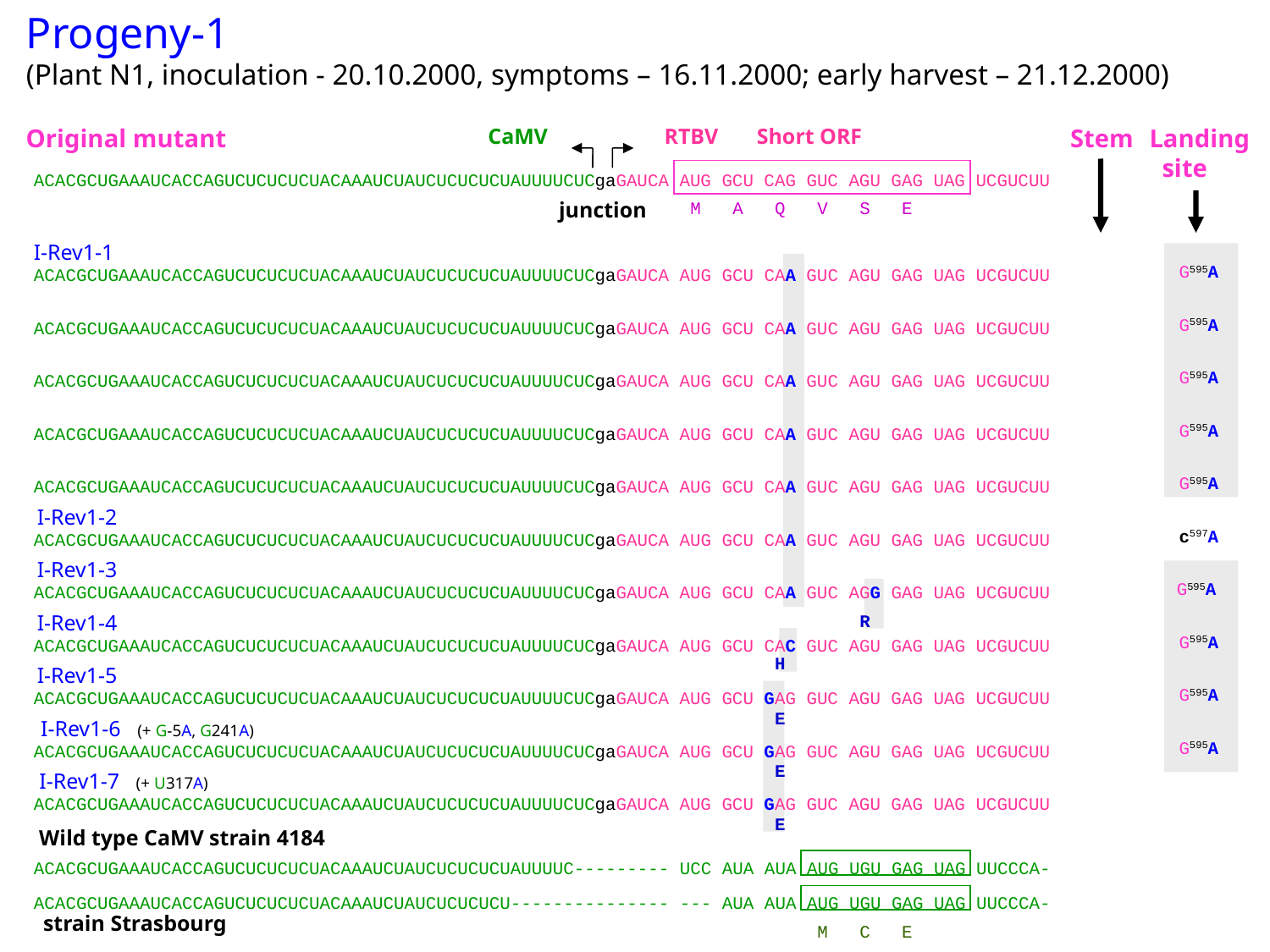

Progeny-1
(Plant N1, inoculation - 20.10.2000, symptoms – 16.11.2000; early harvest – 21.12.2000)
Original mutant
CaMV
RTBV Short ORF
Stem
Landing
 site
ACACGCUGAAAUCACCAGUCUCUCUCUACAAAUCUAUCUCUCUCUAUUUUCUCgaGAUCA AUG GCU CAG GUC AGU GAG UAG UCGUCUU
junction
M A Q V S E
I-Rev1-1
ACACGCUGAAAUCACCAGUCUCUCUCUACAAAUCUAUCUCUCUCUAUUUUCUCgaGAUCA AUG GCU CAA GUC AGU GAG UAG UCGUCUU
G595A
ACACGCUGAAAUCACCAGUCUCUCUCUACAAAUCUAUCUCUCUCUAUUUUCUCgaGAUCA AUG GCU CAA GUC AGU GAG UAG UCGUCUU
G595A
ACACGCUGAAAUCACCAGUCUCUCUCUACAAAUCUAUCUCUCUCUAUUUUCUCgaGAUCA AUG GCU CAA GUC AGU GAG UAG UCGUCUU
G595A
ACACGCUGAAAUCACCAGUCUCUCUCUACAAAUCUAUCUCUCUCUAUUUUCUCgaGAUCA AUG GCU CAA GUC AGU GAG UAG UCGUCUU
G595A
ACACGCUGAAAUCACCAGUCUCUCUCUACAAAUCUAUCUCUCUCUAUUUUCUCgaGAUCA AUG GCU CAA GUC AGU GAG UAG UCGUCUU
G595A
I-Rev1-2
ACACGCUGAAAUCACCAGUCUCUCUCUACAAAUCUAUCUCUCUCUAUUUUCUCgaGAUCA AUG GCU CAA GUC AGU GAG UAG UCGUCUU
c597A
I-Rev1-3
ACACGCUGAAAUCACCAGUCUCUCUCUACAAAUCUAUCUCUCUCUAUUUUCUCgaGAUCA AUG GCU CAA GUC AGG GAG UAG UCGUCUU
G595A
I-Rev1-4
R
ACACGCUGAAAUCACCAGUCUCUCUCUACAAAUCUAUCUCUCUCUAUUUUCUCgaGAUCA AUG GCU CAC GUC AGU GAG UAG UCGUCUU
G595A
H
I-Rev1-5
ACACGCUGAAAUCACCAGUCUCUCUCUACAAAUCUAUCUCUCUCUAUUUUCUCgaGAUCA AUG GCU GAG GUC AGU GAG UAG UCGUCUU
G595A
E
I-Rev1-6 (+ G-5A, G241A)
ACACGCUGAAAUCACCAGUCUCUCUCUACAAAUCUAUCUCUCUCUAUUUUCUCgaGAUCA AUG GCU GAG GUC AGU GAG UAG UCGUCUU
G595A
E
I-Rev1-7 (+ U317A)
ACACGCUGAAAUCACCAGUCUCUCUCUACAAAUCUAUCUCUCUCUAUUUUCUCgaGAUCA AUG GCU GAG GUC AGU GAG UAG UCGUCUU
E
Wild type CaMV strain 4184
ACACGCUGAAAUCACCAGUCUCUCUCUACAAAUCUAUCUCUCUCUAUUUUC--------- UCC AUA AUA AUG UGU GAG UAG UUCCCA-
ACACGCUGAAAUCACCAGUCUCUCUCUACAAAUCUAUCUCUCUCU--------------- --- AUA AUA AUG UGU GAG UAG UUCCCA-
strain Strasbourg
M C E

## Slide 6
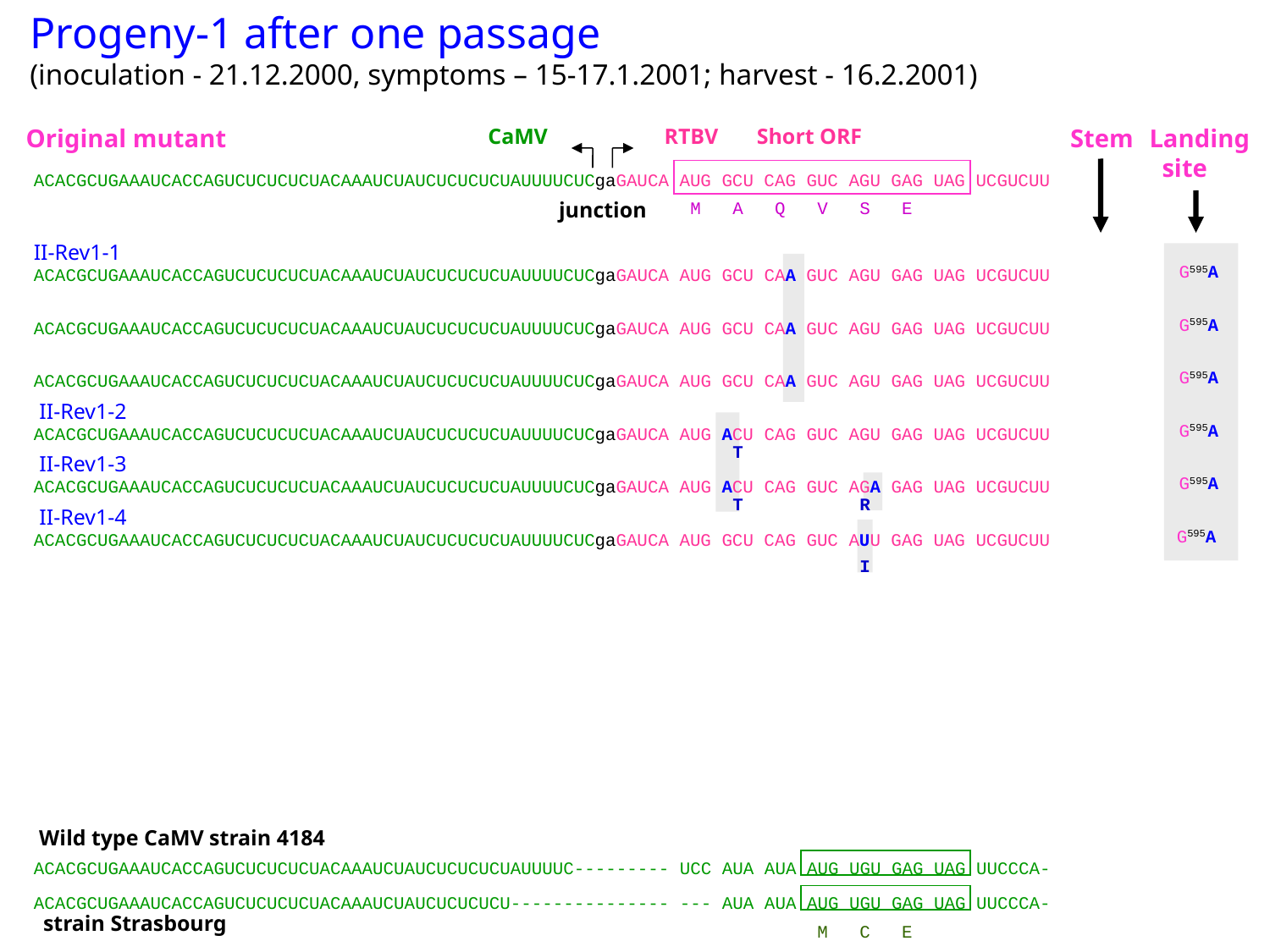

Progeny-1 after one passage
(inoculation - 21.12.2000, symptoms – 15-17.1.2001; harvest - 16.2.2001)
Original mutant
CaMV
RTBV Short ORF
Stem
Landing
 site
ACACGCUGAAAUCACCAGUCUCUCUCUACAAAUCUAUCUCUCUCUAUUUUCUCgaGAUCA AUG GCU CAG GUC AGU GAG UAG UCGUCUU
junction
M A Q V S E
II-Rev1-1
ACACGCUGAAAUCACCAGUCUCUCUCUACAAAUCUAUCUCUCUCUAUUUUCUCgaGAUCA AUG GCU CAA GUC AGU GAG UAG UCGUCUU
G595A
ACACGCUGAAAUCACCAGUCUCUCUCUACAAAUCUAUCUCUCUCUAUUUUCUCgaGAUCA AUG GCU CAA GUC AGU GAG UAG UCGUCUU
G595A
ACACGCUGAAAUCACCAGUCUCUCUCUACAAAUCUAUCUCUCUCUAUUUUCUCgaGAUCA AUG GCU CAA GUC AGU GAG UAG UCGUCUU
G595A
II-Rev1-2
ACACGCUGAAAUCACCAGUCUCUCUCUACAAAUCUAUCUCUCUCUAUUUUCUCgaGAUCA AUG ACU CAG GUC AGU GAG UAG UCGUCUU
G595A
T
II-Rev1-3
ACACGCUGAAAUCACCAGUCUCUCUCUACAAAUCUAUCUCUCUCUAUUUUCUCgaGAUCA AUG ACU CAG GUC AGA GAG UAG UCGUCUU
G595A
T
R
II-Rev1-4
ACACGCUGAAAUCACCAGUCUCUCUCUACAAAUCUAUCUCUCUCUAUUUUCUCgaGAUCA AUG GCU CAG GUC AUU GAG UAG UCGUCUU
G595A
I
Wild type CaMV strain 4184
ACACGCUGAAAUCACCAGUCUCUCUCUACAAAUCUAUCUCUCUCUAUUUUC--------- UCC AUA AUA AUG UGU GAG UAG UUCCCA-
ACACGCUGAAAUCACCAGUCUCUCUCUACAAAUCUAUCUCUCUCU--------------- --- AUA AUA AUG UGU GAG UAG UUCCCA-
strain Strasbourg
M C E

## Slide 7
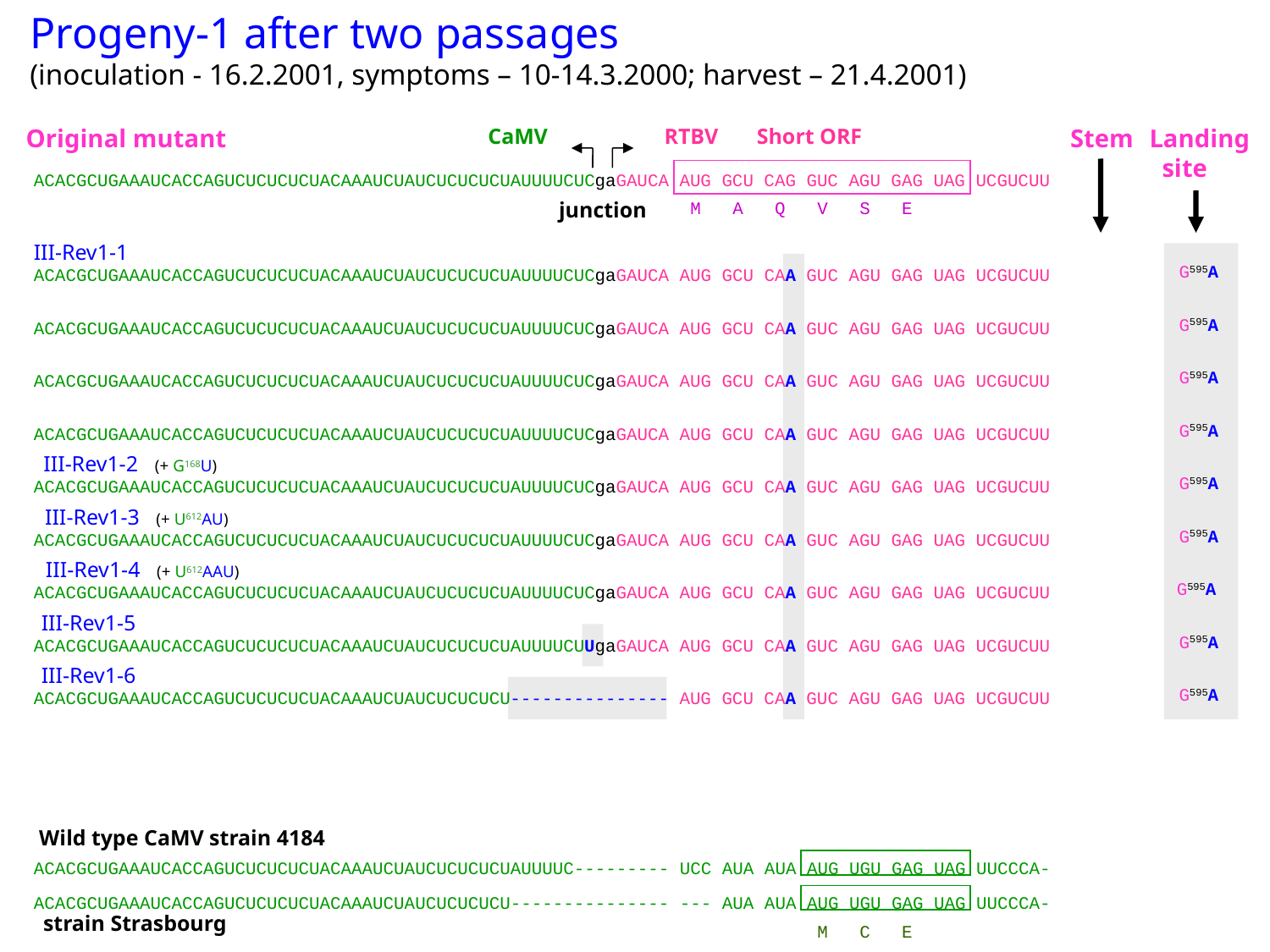

Progeny-1 after two passages
(inoculation - 16.2.2001, symptoms – 10-14.3.2000; harvest – 21.4.2001)
Original mutant
CaMV
RTBV Short ORF
Stem
Landing
 site
ACACGCUGAAAUCACCAGUCUCUCUCUACAAAUCUAUCUCUCUCUAUUUUCUCgaGAUCA AUG GCU CAG GUC AGU GAG UAG UCGUCUU
junction
M A Q V S E
III-Rev1-1
ACACGCUGAAAUCACCAGUCUCUCUCUACAAAUCUAUCUCUCUCUAUUUUCUCgaGAUCA AUG GCU CAA GUC AGU GAG UAG UCGUCUU
G595A
ACACGCUGAAAUCACCAGUCUCUCUCUACAAAUCUAUCUCUCUCUAUUUUCUCgaGAUCA AUG GCU CAA GUC AGU GAG UAG UCGUCUU
G595A
ACACGCUGAAAUCACCAGUCUCUCUCUACAAAUCUAUCUCUCUCUAUUUUCUCgaGAUCA AUG GCU CAA GUC AGU GAG UAG UCGUCUU
G595A
ACACGCUGAAAUCACCAGUCUCUCUCUACAAAUCUAUCUCUCUCUAUUUUCUCgaGAUCA AUG GCU CAA GUC AGU GAG UAG UCGUCUU
G595A
III-Rev1-2 (+ G168U)
ACACGCUGAAAUCACCAGUCUCUCUCUACAAAUCUAUCUCUCUCUAUUUUCUCgaGAUCA AUG GCU CAA GUC AGU GAG UAG UCGUCUU
G595A
III-Rev1-3 (+ U612AU)
ACACGCUGAAAUCACCAGUCUCUCUCUACAAAUCUAUCUCUCUCUAUUUUCUCgaGAUCA AUG GCU CAA GUC AGU GAG UAG UCGUCUU
G595A
III-Rev1-4 (+ U612AAU)
ACACGCUGAAAUCACCAGUCUCUCUCUACAAAUCUAUCUCUCUCUAUUUUCUCgaGAUCA AUG GCU CAA GUC AGU GAG UAG UCGUCUU
G595A
III-Rev1-5
ACACGCUGAAAUCACCAGUCUCUCUCUACAAAUCUAUCUCUCUCUAUUUUCUUgaGAUCA AUG GCU CAA GUC AGU GAG UAG UCGUCUU
G595A
III-Rev1-6
ACACGCUGAAAUCACCAGUCUCUCUCUACAAAUCUAUCUCUCUCU--------------- AUG GCU CAA GUC AGU GAG UAG UCGUCUU
G595A
Wild type CaMV strain 4184
ACACGCUGAAAUCACCAGUCUCUCUCUACAAAUCUAUCUCUCUCUAUUUUC--------- UCC AUA AUA AUG UGU GAG UAG UUCCCA-
ACACGCUGAAAUCACCAGUCUCUCUCUACAAAUCUAUCUCUCUCU--------------- --- AUA AUA AUG UGU GAG UAG UUCCCA-
strain Strasbourg
M C E

## Slide 8
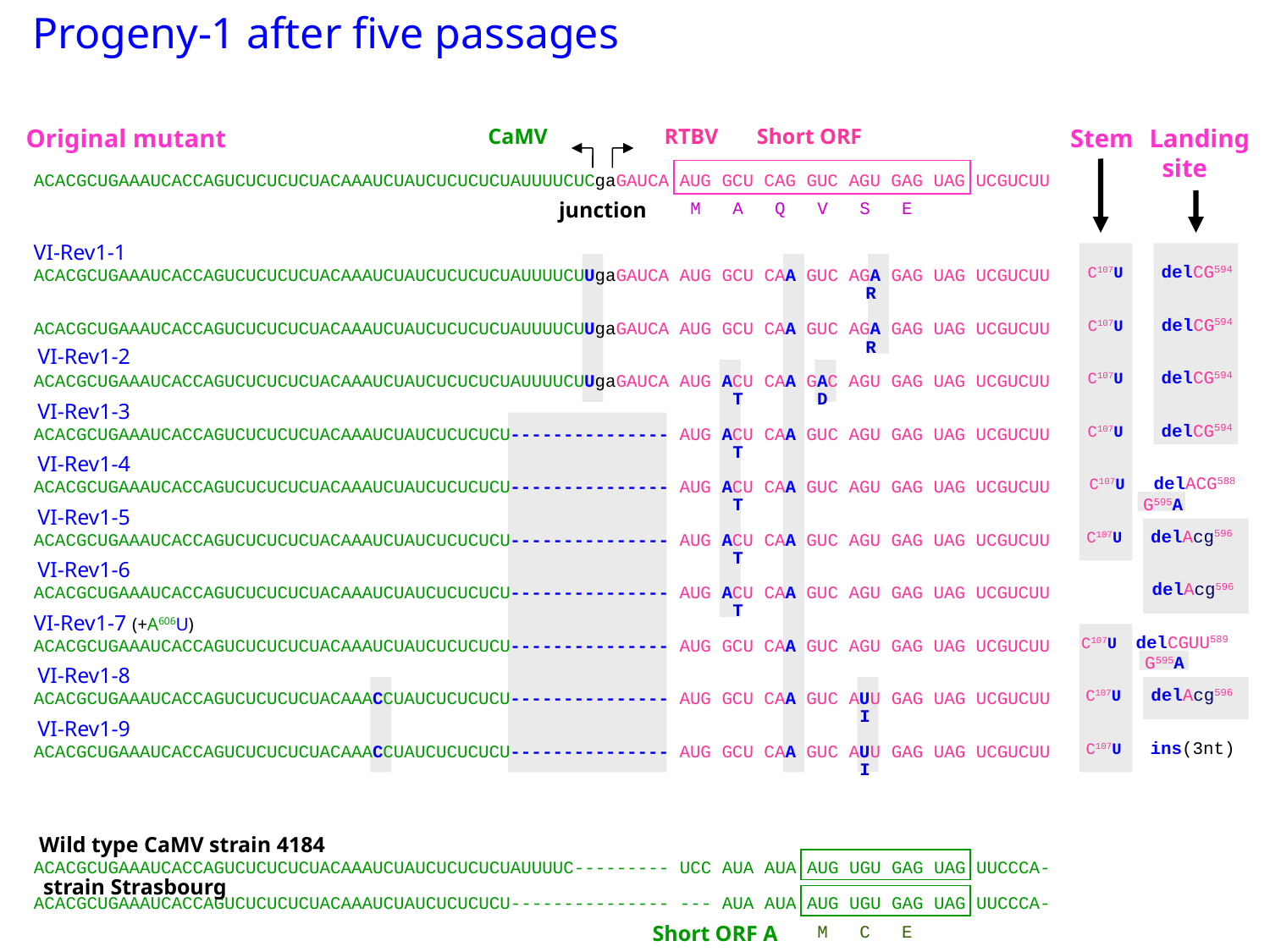

Progeny-1 after five passages
Original mutant
CaMV
RTBV Short ORF
Stem
Landing
 site
ACACGCUGAAAUCACCAGUCUCUCUCUACAAAUCUAUCUCUCUCUAUUUUCUCgaGAUCA AUG GCU CAG GUC AGU GAG UAG UCGUCUU
junction
M A Q V S E
VI-Rev1-1
ACACGCUGAAAUCACCAGUCUCUCUCUACAAAUCUAUCUCUCUCUAUUUUCUUgaGAUCA AUG GCU CAA GUC AGA GAG UAG UCGUCUU
C107U delCG594
R
ACACGCUGAAAUCACCAGUCUCUCUCUACAAAUCUAUCUCUCUCUAUUUUCUUgaGAUCA AUG GCU CAA GUC AGA GAG UAG UCGUCUU
C107U delCG594
R
VI-Rev1-2
ACACGCUGAAAUCACCAGUCUCUCUCUACAAAUCUAUCUCUCUCUAUUUUCUUgaGAUCA AUG ACU CAA GAC AGU GAG UAG UCGUCUU
C107U delCG594
T
D
VI-Rev1-3
ACACGCUGAAAUCACCAGUCUCUCUCUACAAAUCUAUCUCUCUCU--------------- AUG ACU CAA GUC AGU GAG UAG UCGUCUU
C107U delCG594
T
VI-Rev1-4
ACACGCUGAAAUCACCAGUCUCUCUCUACAAAUCUAUCUCUCUCU--------------- AUG ACU CAA GUC AGU GAG UAG UCGUCUU
 C107U delACG588
 G595A
T
VI-Rev1-5
ACACGCUGAAAUCACCAGUCUCUCUCUACAAAUCUAUCUCUCUCU--------------- AUG ACU CAA GUC AGU GAG UAG UCGUCUU
C107U delAcg596
T
VI-Rev1-6
ACACGCUGAAAUCACCAGUCUCUCUCUACAAAUCUAUCUCUCUCU--------------- AUG ACU CAA GUC AGU GAG UAG UCGUCUU
 delAcg596
T
VI-Rev1-7 (+A606U)
ACACGCUGAAAUCACCAGUCUCUCUCUACAAAUCUAUCUCUCUCU--------------- AUG GCU CAA GUC AGU GAG UAG UCGUCUU
C107U delCGUU589
 G595A
VI-Rev1-8
ACACGCUGAAAUCACCAGUCUCUCUCUACAAACCUAUCUCUCUCU--------------- AUG GCU CAA GUC AUU GAG UAG UCGUCUU
C107U delAcg596
I
VI-Rev1-9
ACACGCUGAAAUCACCAGUCUCUCUCUACAAACCUAUCUCUCUCU--------------- AUG GCU CAA GUC AUU GAG UAG UCGUCUU
C107U ins(3nt)
I
Wild type CaMV strain 4184
ACACGCUGAAAUCACCAGUCUCUCUCUACAAAUCUAUCUCUCUCUAUUUUC--------- UCC AUA AUA AUG UGU GAG UAG UUCCCA-
strain Strasbourg
ACACGCUGAAAUCACCAGUCUCUCUCUACAAAUCUAUCUCUCUCU--------------- --- AUA AUA AUG UGU GAG UAG UUCCCA-
Short ORF A
M C E

## Slide 9
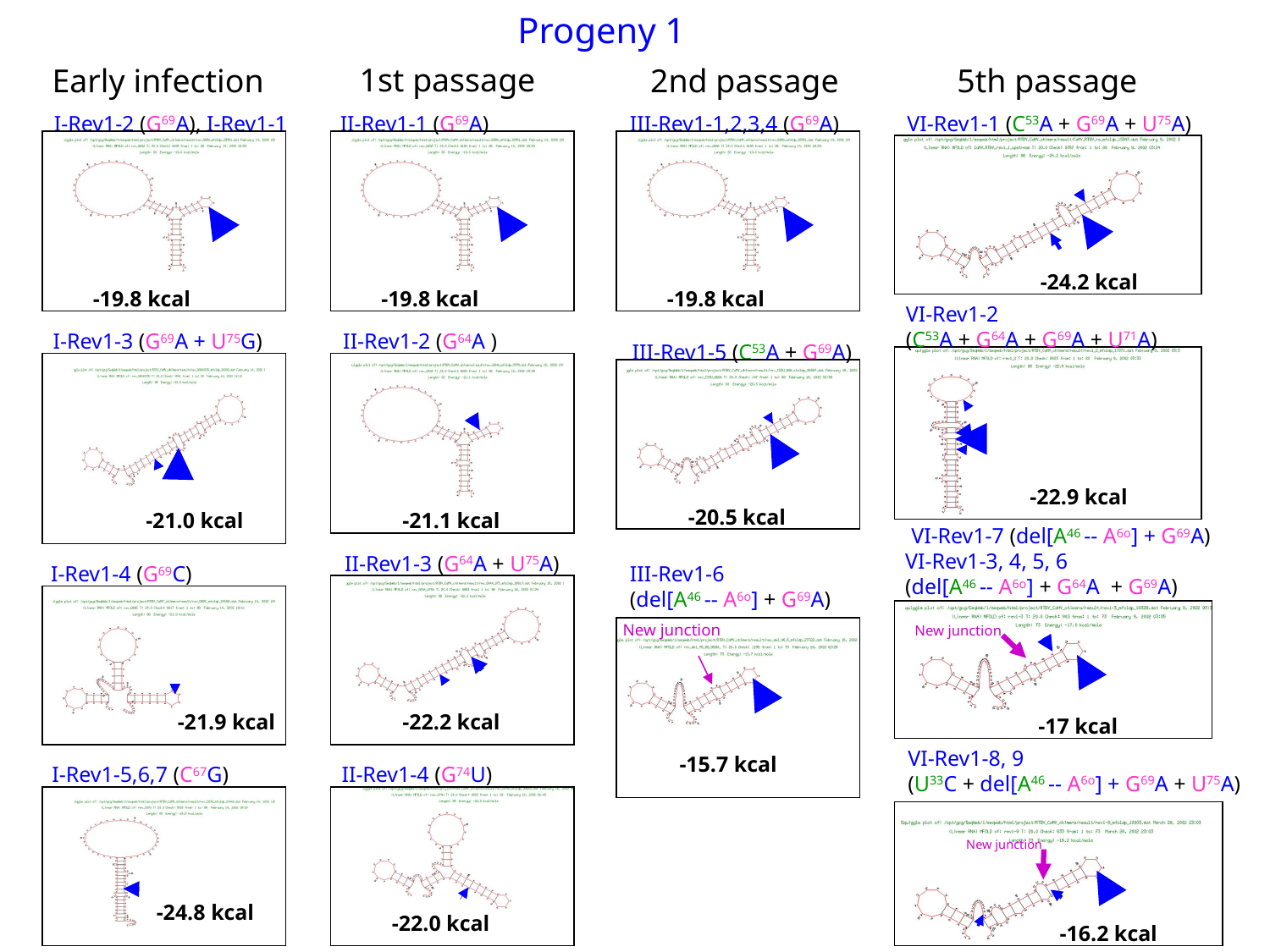

Progeny 1
1st passage
Early infection
2nd passage
5th passage
I-Rev1-2 (G69A), I-Rev1-1
II-Rev1-1 (G69A)
III-Rev1-1,2,3,4 (G69A)
VI-Rev1-1 (C53A + G69A + U75A)
-24.2 kcal
-19.8 kcal
-19.8 kcal
-19.8 kcal
VI-Rev1-2
(C53A + G64A + G69A + U71A)
I-Rev1-3 (G69A + U75G)
II-Rev1-2 (G64A )
III-Rev1-5 (C53A + G69A)
-22.9 kcal
-20.5 kcal
-21.0 kcal
-21.1 kcal
VI-Rev1-7 (del[A46 -- A6o] + G69A)
VI-Rev1-3, 4, 5, 6
(del[A46 -- A6o] + G64A + G69A)
II-Rev1-3 (G64A + U75A)
I-Rev1-4 (G69C)
III-Rev1-6
(del[A46 -- A6o] + G69A)
New junction
New junction
-21.9 kcal
-22.2 kcal
-17 kcal
VI-Rev1-8, 9
(U33C + del[A46 -- A6o] + G69A + U75A)
-15.7 kcal
I-Rev1-5,6,7 (C67G)
II-Rev1-4 (G74U)
New junction
-24.8 kcal
-22.0 kcal
-16.2 kcal

## Slide 10
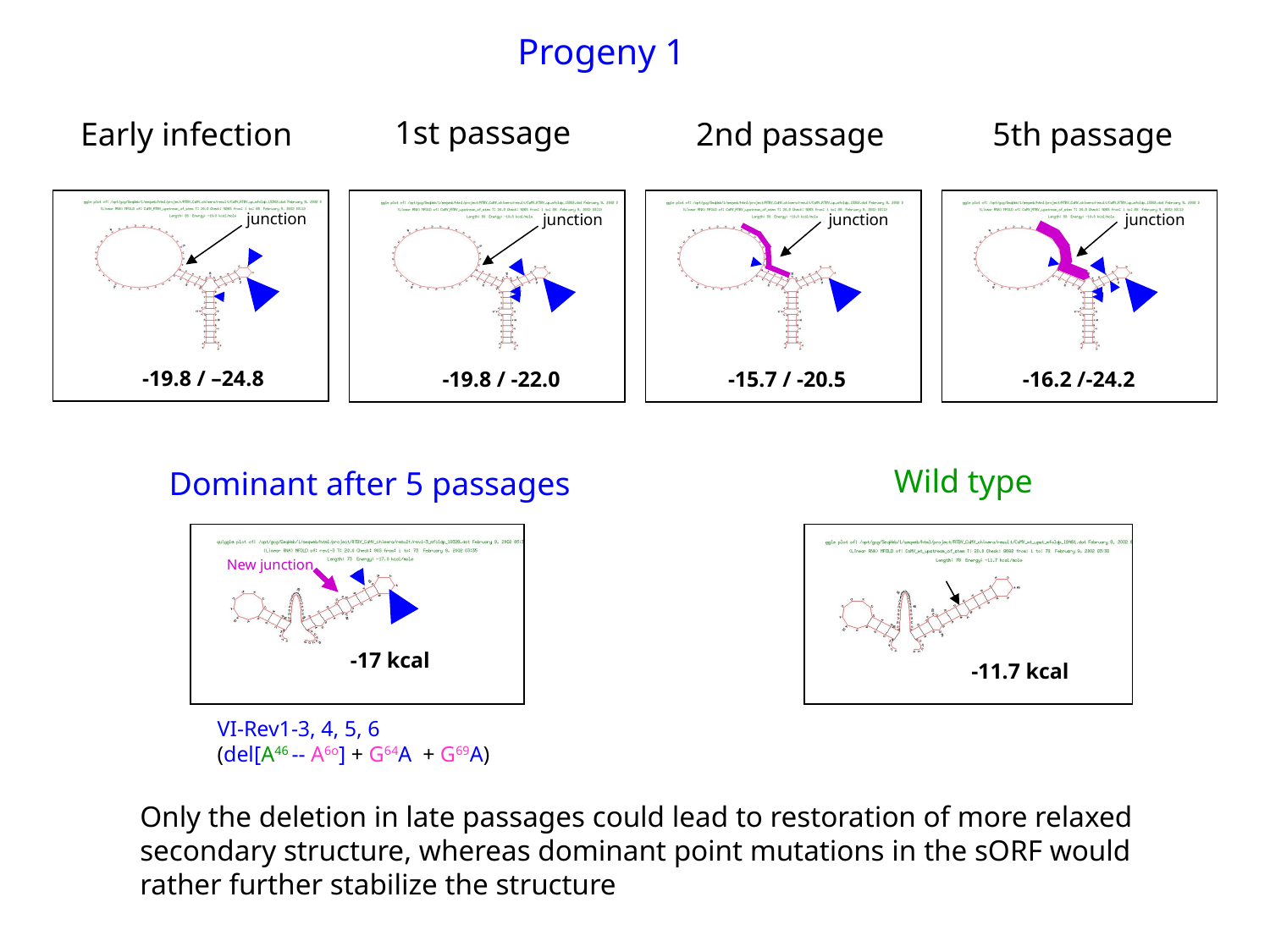

Progeny 1
1st passage
Early infection
2nd passage
5th passage
junction
junction
junction
junction
-19.8 / –24.8
-19.8 / -22.0
-15.7 / -20.5
-16.2 /-24.2
Wild type
Dominant after 5 passages
New junction
-17 kcal
-11.7 kcal
VI-Rev1-3, 4, 5, 6
(del[A46 -- A6o] + G64A + G69A)
Only the deletion in late passages could lead to restoration of more relaxed
secondary structure, whereas dominant point mutations in the sORF would
rather further stabilize the structure

## Slide 11
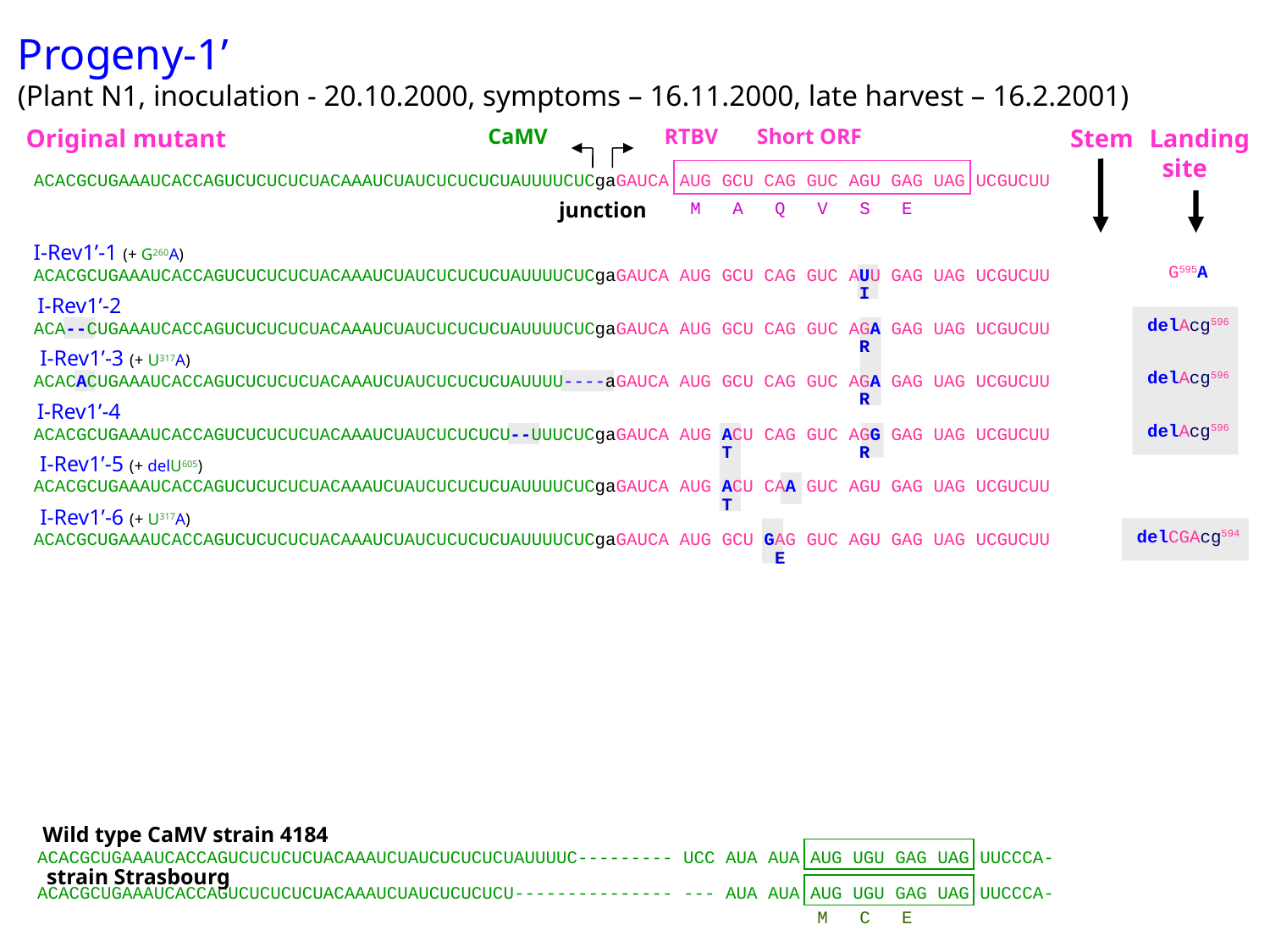

Progeny-1’
(Plant N1, inoculation - 20.10.2000, symptoms – 16.11.2000, late harvest – 16.2.2001)
Original mutant
CaMV
RTBV Short ORF
Stem
Landing
 site
ACACGCUGAAAUCACCAGUCUCUCUCUACAAAUCUAUCUCUCUCUAUUUUCUCgaGAUCA AUG GCU CAG GUC AGU GAG UAG UCGUCUU
junction
M A Q V S E
I-Rev1’-1 (+ G260A)
ACACGCUGAAAUCACCAGUCUCUCUCUACAAAUCUAUCUCUCUCUAUUUUCUCgaGAUCA AUG GCU CAG GUC AUU GAG UAG UCGUCUU
 G595A
I
I-Rev1’-2
ACA--CUGAAAUCACCAGUCUCUCUCUACAAAUCUAUCUCUCUCUAUUUUCUCgaGAUCA AUG GCU CAG GUC AGA GAG UAG UCGUCUU
 delAcg596
R
I-Rev1’-3 (+ U317A)
ACACACUGAAAUCACCAGUCUCUCUCUACAAAUCUAUCUCUCUCUAUUUU----aGAUCA AUG GCU CAG GUC AGA GAG UAG UCGUCUU
 delAcg596
R
I-Rev1’-4
ACACGCUGAAAUCACCAGUCUCUCUCUACAAAUCUAUCUCUCUCU--UUUCUCgaGAUCA AUG ACU CAG GUC AGG GAG UAG UCGUCUU
 delAcg596
T
R
I-Rev1’-5 (+ delU605)
ACACGCUGAAAUCACCAGUCUCUCUCUACAAAUCUAUCUCUCUCUAUUUUCUCgaGAUCA AUG ACU CAA GUC AGU GAG UAG UCGUCUU
T
I-Rev1’-6 (+ U317A)
 delCGAcg594
ACACGCUGAAAUCACCAGUCUCUCUCUACAAAUCUAUCUCUCUCUAUUUUCUCgaGAUCA AUG GCU GAG GUC AGU GAG UAG UCGUCUU
E
Wild type CaMV strain 4184
ACACGCUGAAAUCACCAGUCUCUCUCUACAAAUCUAUCUCUCUCUAUUUUC--------- UCC AUA AUA AUG UGU GAG UAG UUCCCA-
strain Strasbourg
ACACGCUGAAAUCACCAGUCUCUCUCUACAAAUCUAUCUCUCUCU--------------- --- AUA AUA AUG UGU GAG UAG UUCCCA-
M C E

## Slide 12
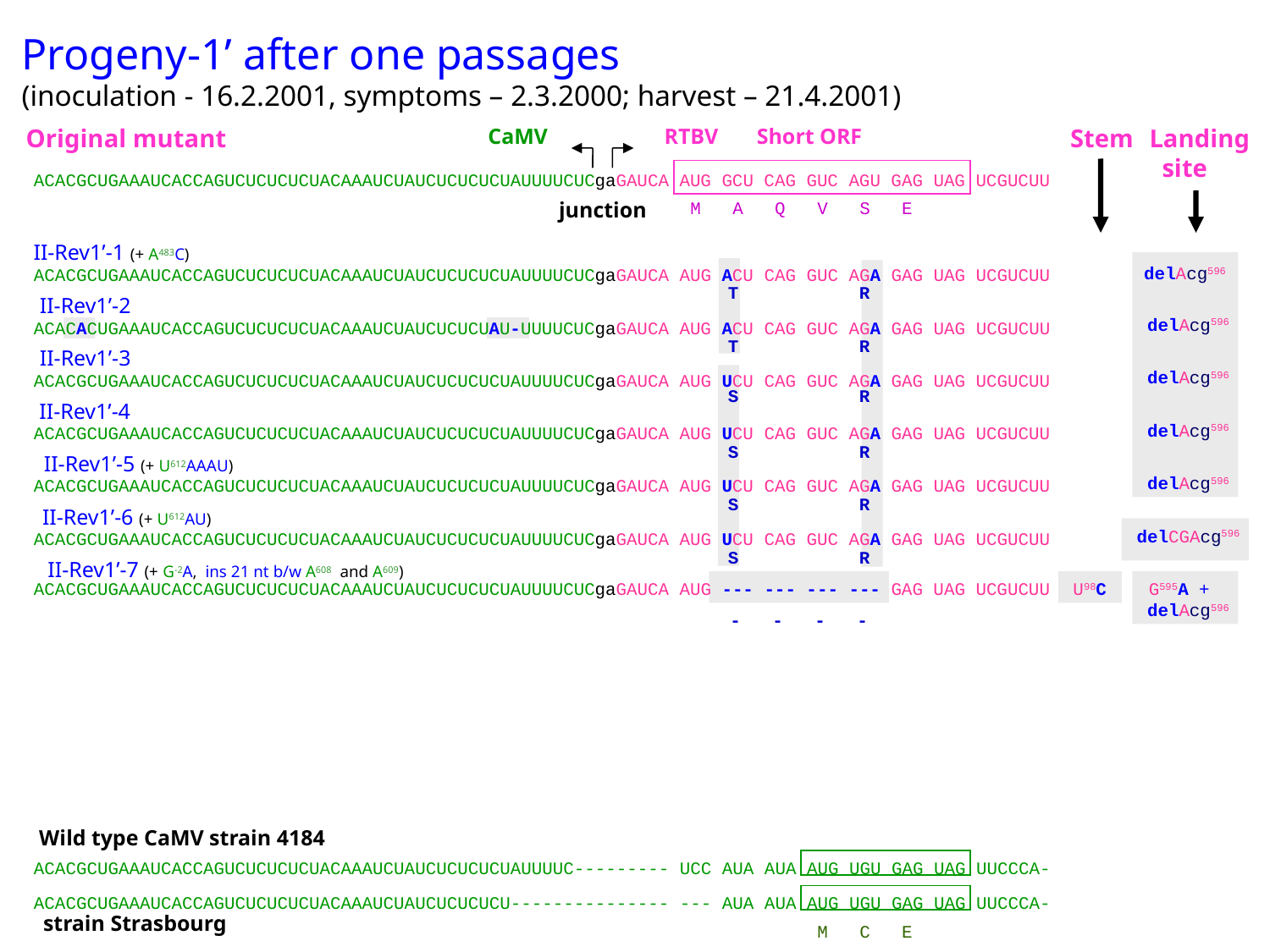

Progeny-1’ after one passages
(inoculation - 16.2.2001, symptoms – 2.3.2000; harvest – 21.4.2001)
Original mutant
CaMV
RTBV Short ORF
Stem
Landing
 site
ACACGCUGAAAUCACCAGUCUCUCUCUACAAAUCUAUCUCUCUCUAUUUUCUCgaGAUCA AUG GCU CAG GUC AGU GAG UAG UCGUCUU
junction
M A Q V S E
II-Rev1’-1 (+ A483C)
ACACGCUGAAAUCACCAGUCUCUCUCUACAAAUCUAUCUCUCUCUAUUUUCUCgaGAUCA AUG ACU CAG GUC AGA GAG UAG UCGUCUU
 delAcg596
T
R
II-Rev1’-2
ACACACUGAAAUCACCAGUCUCUCUCUACAAAUCUAUCUCUCUAU-UUUUCUCgaGAUCA AUG ACU CAG GUC AGA GAG UAG UCGUCUU
 delAcg596
T
R
II-Rev1’-3
ACACGCUGAAAUCACCAGUCUCUCUCUACAAAUCUAUCUCUCUCUAUUUUCUCgaGAUCA AUG UCU CAG GUC AGA GAG UAG UCGUCUU
 delAcg596
S
R
II-Rev1’-4
 delAcg596
ACACGCUGAAAUCACCAGUCUCUCUCUACAAAUCUAUCUCUCUCUAUUUUCUCgaGAUCA AUG UCU CAG GUC AGA GAG UAG UCGUCUU
S
R
II-Rev1’-5 (+ U612AAAU)
 delAcg596
ACACGCUGAAAUCACCAGUCUCUCUCUACAAAUCUAUCUCUCUCUAUUUUCUCgaGAUCA AUG UCU CAG GUC AGA GAG UAG UCGUCUU
S
R
II-Rev1’-6 (+ U612AU)
 delCGAcg596
ACACGCUGAAAUCACCAGUCUCUCUCUACAAAUCUAUCUCUCUCUAUUUUCUCgaGAUCA AUG UCU CAG GUC AGA GAG UAG UCGUCUU
S
R
II-Rev1’-7 (+ G-2A, ins 21 nt b/w A608 and A609)
ACACGCUGAAAUCACCAGUCUCUCUCUACAAAUCUAUCUCUCUCUAUUUUCUCgaGAUCA AUG --- --- --- --- GAG UAG UCGUCUU
U98C G595A +
 delAcg596
- - - -
Wild type CaMV strain 4184
ACACGCUGAAAUCACCAGUCUCUCUCUACAAAUCUAUCUCUCUCUAUUUUC--------- UCC AUA AUA AUG UGU GAG UAG UUCCCA-
ACACGCUGAAAUCACCAGUCUCUCUCUACAAAUCUAUCUCUCUCU--------------- --- AUA AUA AUG UGU GAG UAG UUCCCA-
strain Strasbourg
M C E

## Slide 13
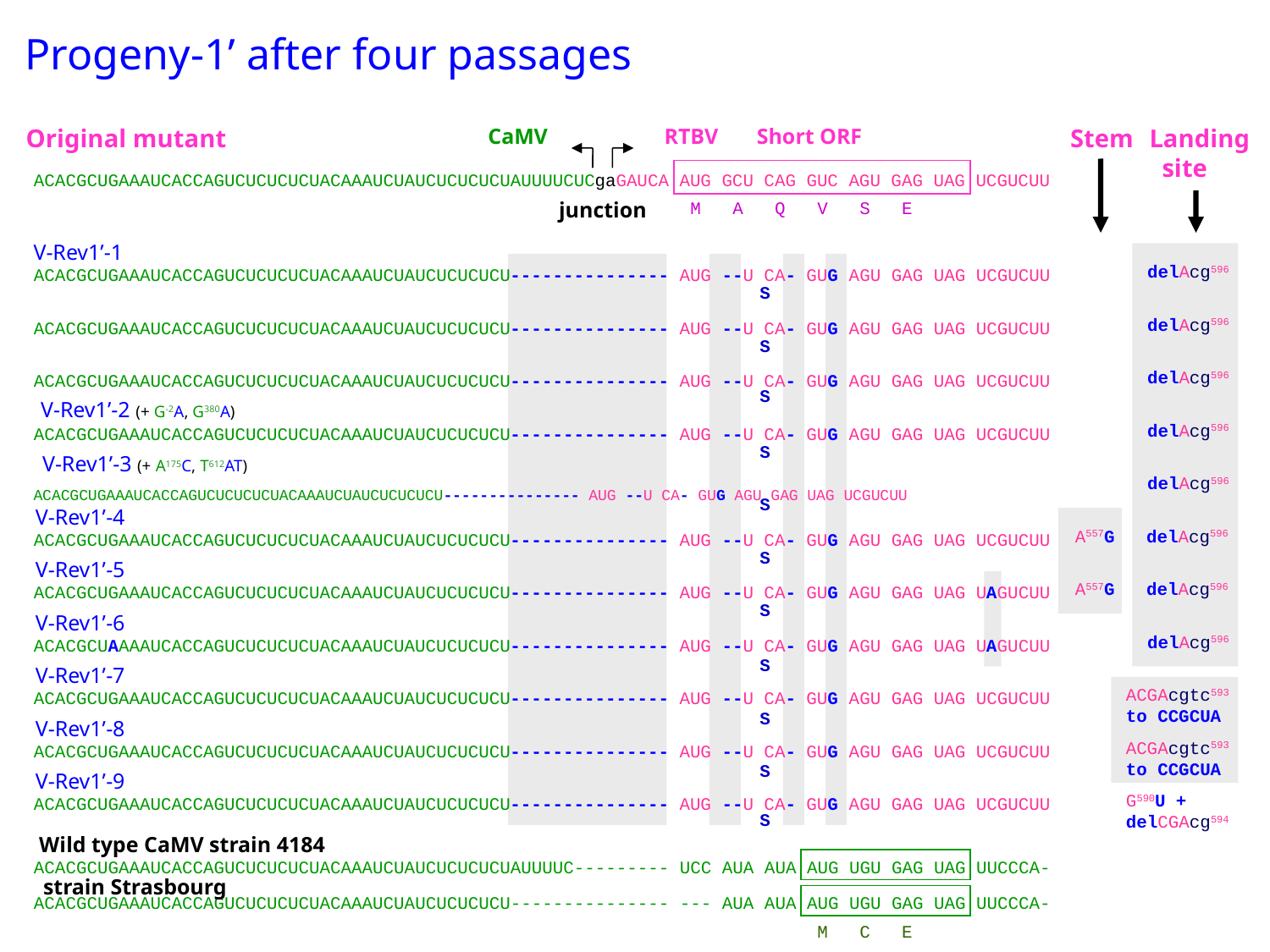

Progeny-1’ after four passages
Original mutant
CaMV
RTBV Short ORF
Stem
Landing
 site
ACACGCUGAAAUCACCAGUCUCUCUCUACAAAUCUAUCUCUCUCUAUUUUCUCgaGAUCA AUG GCU CAG GUC AGU GAG UAG UCGUCUU
junction
M A Q V S E
V-Rev1’-1
ACACGCUGAAAUCACCAGUCUCUCUCUACAAAUCUAUCUCUCUCU--------------- AUG --U CA- GUG AGU GAG UAG UCGUCUU
 delAcg596
S
ACACGCUGAAAUCACCAGUCUCUCUCUACAAAUCUAUCUCUCUCU--------------- AUG --U CA- GUG AGU GAG UAG UCGUCUU
 delAcg596
S
ACACGCUGAAAUCACCAGUCUCUCUCUACAAAUCUAUCUCUCUCU--------------- AUG --U CA- GUG AGU GAG UAG UCGUCUU
 delAcg596
S
V-Rev1’-2 (+ G-2A, G380A)
ACACGCUGAAAUCACCAGUCUCUCUCUACAAAUCUAUCUCUCUCU--------------- AUG --U CA- GUG AGU GAG UAG UCGUCUU
 delAcg596
S
V-Rev1’-3 (+ A175C, T612AT)
 delAcg596
ACACGCUGAAAUCACCAGUCUCUCUCUACAAAUCUAUCUCUCUCU--------------- AUG --U CA- GUG AGU GAG UAG UCGUCUU
S
V-Rev1’-4
ACACGCUGAAAUCACCAGUCUCUCUCUACAAAUCUAUCUCUCUCU--------------- AUG --U CA- GUG AGU GAG UAG UCGUCUU
A557G delAcg596
S
V-Rev1’-5
ACACGCUGAAAUCACCAGUCUCUCUCUACAAAUCUAUCUCUCUCU--------------- AUG --U CA- GUG AGU GAG UAG UAGUCUU
A557G delAcg596
S
V-Rev1’-6
ACACGCUAAAAUCACCAGUCUCUCUCUACAAAUCUAUCUCUCUCU--------------- AUG --U CA- GUG AGU GAG UAG UAGUCUU
 delAcg596
S
V-Rev1’-7
ACACGCUGAAAUCACCAGUCUCUCUCUACAAAUCUAUCUCUCUCU--------------- AUG --U CA- GUG AGU GAG UAG UCGUCUU
ACGAcgtc593
to CCGCUA
S
V-Rev1’-8
ACACGCUGAAAUCACCAGUCUCUCUCUACAAAUCUAUCUCUCUCU--------------- AUG --U CA- GUG AGU GAG UAG UCGUCUU
ACGAcgtc593
to CCGCUA
S
V-Rev1’-9
ACACGCUGAAAUCACCAGUCUCUCUCUACAAAUCUAUCUCUCUCU--------------- AUG --U CA- GUG AGU GAG UAG UCGUCUU
G590U +
delCGAcg594
S
Wild type CaMV strain 4184
ACACGCUGAAAUCACCAGUCUCUCUCUACAAAUCUAUCUCUCUCUAUUUUC--------- UCC AUA AUA AUG UGU GAG UAG UUCCCA-
strain Strasbourg
ACACGCUGAAAUCACCAGUCUCUCUCUACAAAUCUAUCUCUCUCU--------------- --- AUA AUA AUG UGU GAG UAG UUCCCA-
M C E

## Slide 14
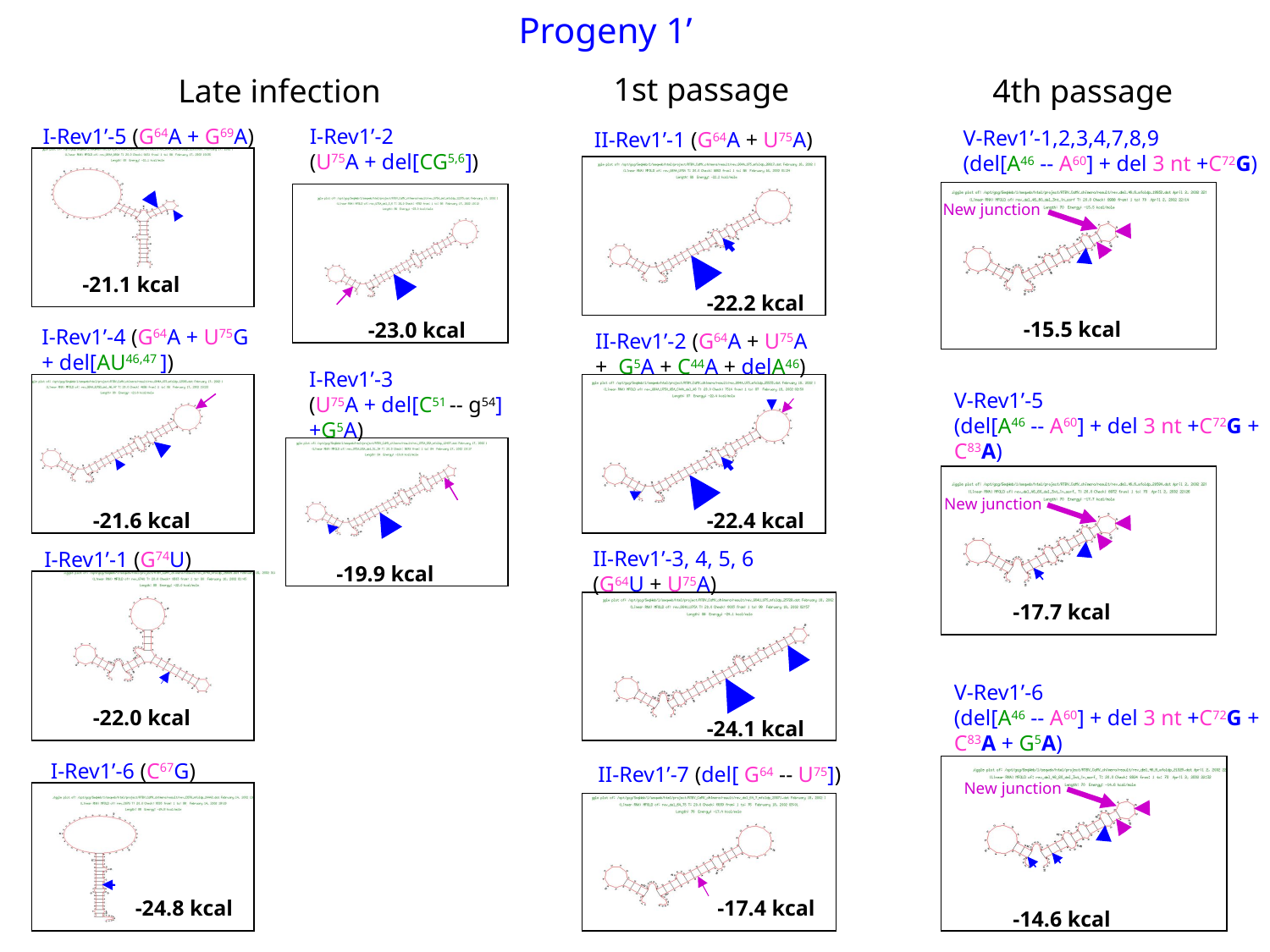

Progeny 1’
1st passage
Late infection
4th passage
I-Rev1’-5 (G64A + G69A)
I-Rev1’-2
(U75A + del[CG5,6])
V-Rev1’-1,2,3,4,7,8,9
(del[A46 -- A60] + del 3 nt +C72G)
II-Rev1’-1 (G64A + U75A)
New junction
-21.1 kcal
-22.2 kcal
-15.5 kcal
-23.0 kcal
I-Rev1’-4 (G64A + U75G
+ del[AU46,47 ])
II-Rev1’-2 (G64A + U75A
+ G5A + C44A + delA46)
I-Rev1’-3
(U75A + del[C51 -- g54]
+G5A)
V-Rev1’-5
(del[A46 -- A60] + del 3 nt +C72G +
C83A)
New junction
-21.6 kcal
-22.4 kcal
I-Rev1’-1 (G74U)
II-Rev1’-3, 4, 5, 6
(G64U + U75A)
-19.9 kcal
-17.7 kcal
V-Rev1’-6
(del[A46 -- A60] + del 3 nt +C72G +
C83A + G5A)
-22.0 kcal
-24.1 kcal
I-Rev1’-6 (C67G)
II-Rev1’-7 (del[ G64 -- U75])
New junction
-24.8 kcal
-17.4 kcal
-14.6 kcal

## Slide 15
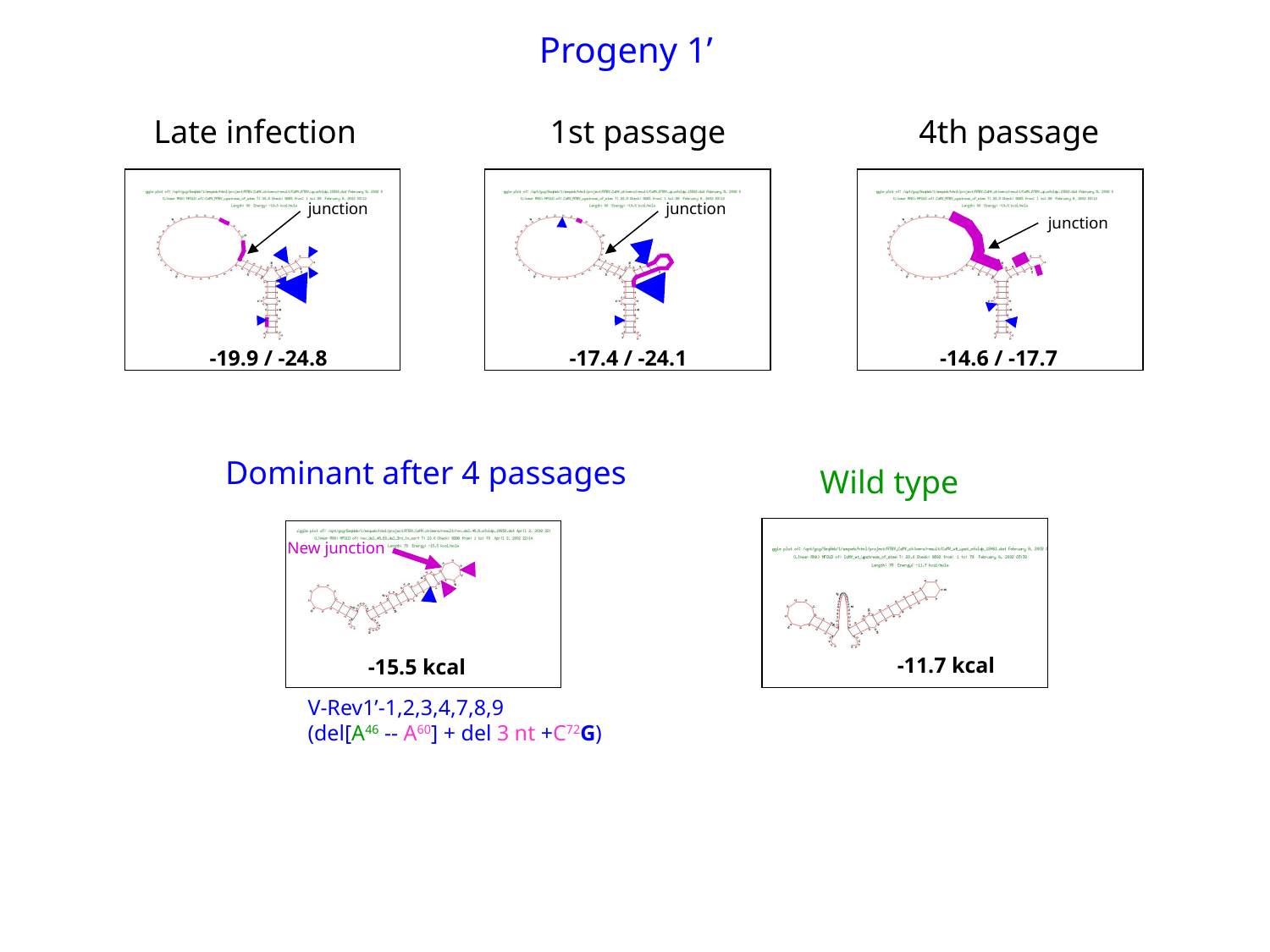

Progeny 1’
Late infection
1st passage
4th passage
junction
junction
junction
-19.9 / -24.8
-17.4 / -24.1
-14.6 / -17.7
Dominant after 4 passages
Wild type
New junction
-11.7 kcal
-15.5 kcal
V-Rev1’-1,2,3,4,7,8,9
(del[A46 -- A60] + del 3 nt +C72G)

## Slide 16
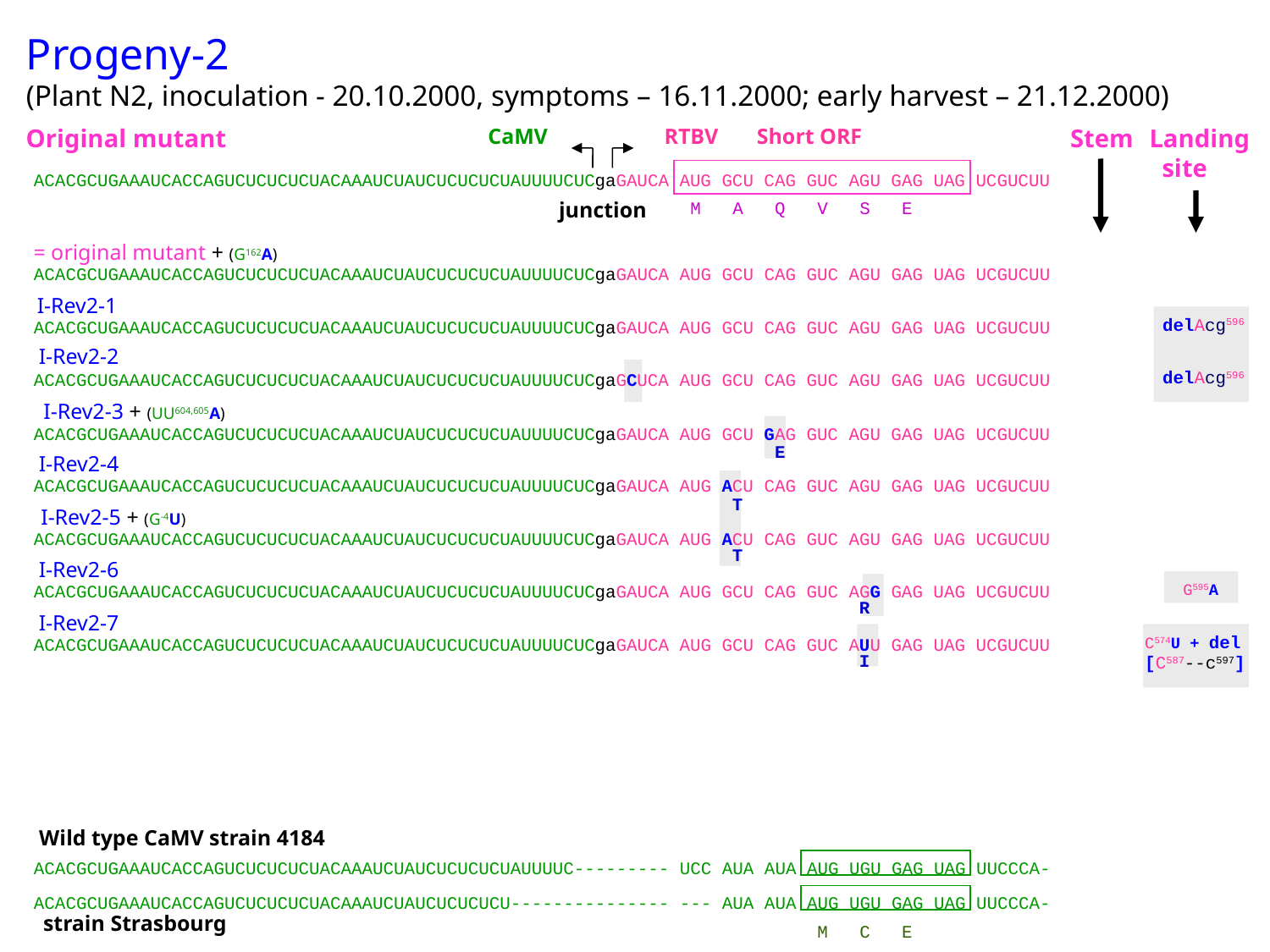

Progeny-2
(Plant N2, inoculation - 20.10.2000, symptoms – 16.11.2000; early harvest – 21.12.2000)
Original mutant
CaMV
RTBV Short ORF
Stem
Landing
 site
ACACGCUGAAAUCACCAGUCUCUCUCUACAAAUCUAUCUCUCUCUAUUUUCUCgaGAUCA AUG GCU CAG GUC AGU GAG UAG UCGUCUU
junction
M A Q V S E
= original mutant + (G162A)
ACACGCUGAAAUCACCAGUCUCUCUCUACAAAUCUAUCUCUCUCUAUUUUCUCgaGAUCA AUG GCU CAG GUC AGU GAG UAG UCGUCUU
I-Rev2-1
 delAcg596
ACACGCUGAAAUCACCAGUCUCUCUCUACAAAUCUAUCUCUCUCUAUUUUCUCgaGAUCA AUG GCU CAG GUC AGU GAG UAG UCGUCUU
I-Rev2-2
 delAcg596
ACACGCUGAAAUCACCAGUCUCUCUCUACAAAUCUAUCUCUCUCUAUUUUCUCgaGCUCA AUG GCU CAG GUC AGU GAG UAG UCGUCUU
I-Rev2-3 + (UU604,605A)
ACACGCUGAAAUCACCAGUCUCUCUCUACAAAUCUAUCUCUCUCUAUUUUCUCgaGAUCA AUG GCU GAG GUC AGU GAG UAG UCGUCUU
E
I-Rev2-4
ACACGCUGAAAUCACCAGUCUCUCUCUACAAAUCUAUCUCUCUCUAUUUUCUCgaGAUCA AUG ACU CAG GUC AGU GAG UAG UCGUCUU
T
I-Rev2-5 + (G-4U)
ACACGCUGAAAUCACCAGUCUCUCUCUACAAAUCUAUCUCUCUCUAUUUUCUCgaGAUCA AUG ACU CAG GUC AGU GAG UAG UCGUCUU
T
I-Rev2-6
 G595A
ACACGCUGAAAUCACCAGUCUCUCUCUACAAAUCUAUCUCUCUCUAUUUUCUCgaGAUCA AUG GCU CAG GUC AGG GAG UAG UCGUCUU
R
I-Rev2-7
 C574U + del
 [C587--c597]
ACACGCUGAAAUCACCAGUCUCUCUCUACAAAUCUAUCUCUCUCUAUUUUCUCgaGAUCA AUG GCU CAG GUC AUU GAG UAG UCGUCUU
I
Wild type CaMV strain 4184
ACACGCUGAAAUCACCAGUCUCUCUCUACAAAUCUAUCUCUCUCUAUUUUC--------- UCC AUA AUA AUG UGU GAG UAG UUCCCA-
ACACGCUGAAAUCACCAGUCUCUCUCUACAAAUCUAUCUCUCUCU--------------- --- AUA AUA AUG UGU GAG UAG UUCCCA-
strain Strasbourg
M C E

## Slide 17
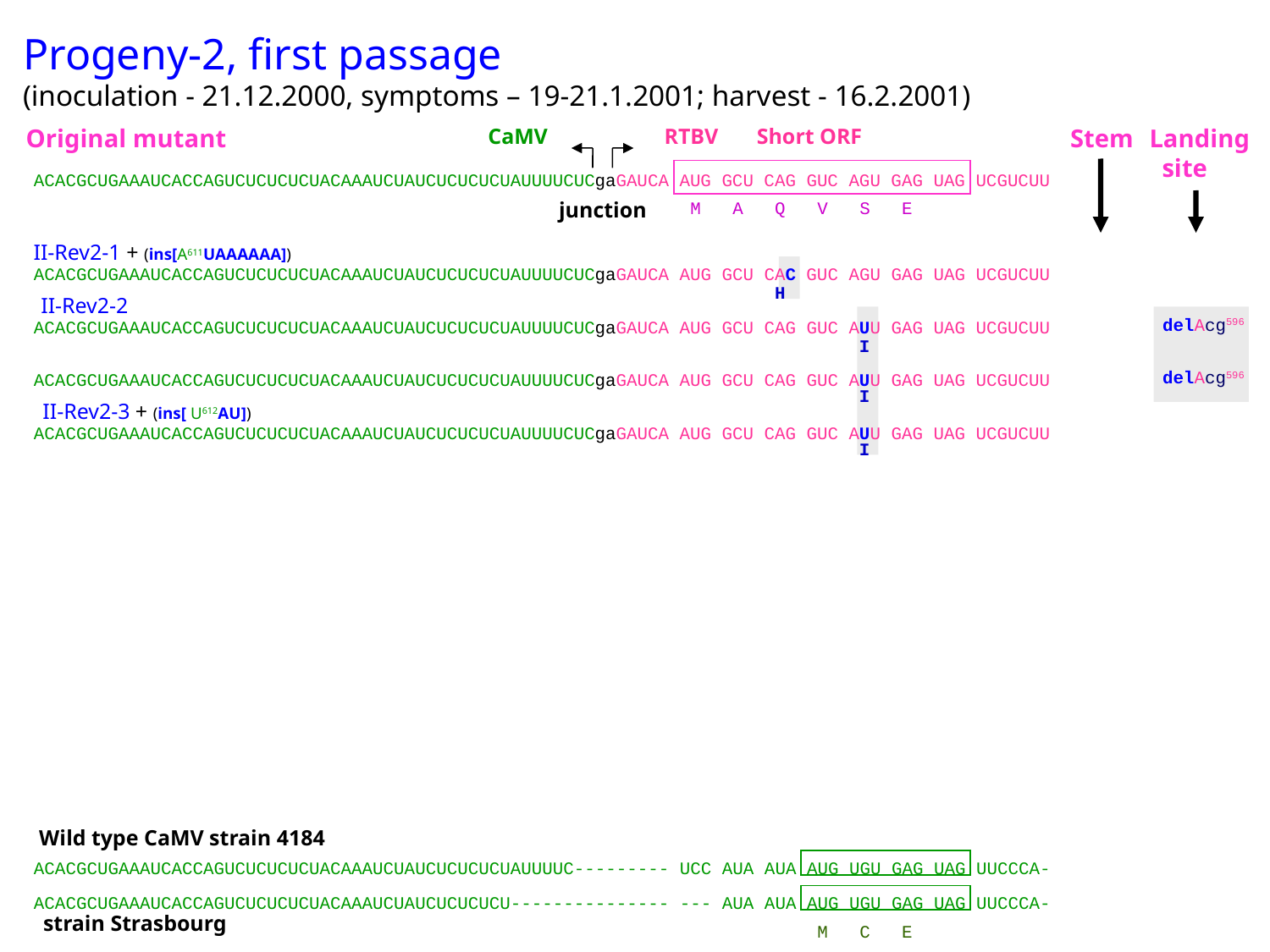

Progeny-2, first passage
(inoculation - 21.12.2000, symptoms – 19-21.1.2001; harvest - 16.2.2001)
Original mutant
CaMV
RTBV Short ORF
Stem
Landing
 site
ACACGCUGAAAUCACCAGUCUCUCUCUACAAAUCUAUCUCUCUCUAUUUUCUCgaGAUCA AUG GCU CAG GUC AGU GAG UAG UCGUCUU
junction
M A Q V S E
II-Rev2-1 + (ins[A611UAAAAAA])
ACACGCUGAAAUCACCAGUCUCUCUCUACAAAUCUAUCUCUCUCUAUUUUCUCgaGAUCA AUG GCU CAC GUC AGU GAG UAG UCGUCUU
H
II-Rev2-2
 delAcg596
ACACGCUGAAAUCACCAGUCUCUCUCUACAAAUCUAUCUCUCUCUAUUUUCUCgaGAUCA AUG GCU CAG GUC AUU GAG UAG UCGUCUU
I
 delAcg596
ACACGCUGAAAUCACCAGUCUCUCUCUACAAAUCUAUCUCUCUCUAUUUUCUCgaGAUCA AUG GCU CAG GUC AUU GAG UAG UCGUCUU
I
II-Rev2-3 + (ins[ U612AU])
ACACGCUGAAAUCACCAGUCUCUCUCUACAAAUCUAUCUCUCUCUAUUUUCUCgaGAUCA AUG GCU CAG GUC AUU GAG UAG UCGUCUU
I
Wild type CaMV strain 4184
ACACGCUGAAAUCACCAGUCUCUCUCUACAAAUCUAUCUCUCUCUAUUUUC--------- UCC AUA AUA AUG UGU GAG UAG UUCCCA-
ACACGCUGAAAUCACCAGUCUCUCUCUACAAAUCUAUCUCUCUCU--------------- --- AUA AUA AUG UGU GAG UAG UUCCCA-
strain Strasbourg
M C E

## Slide 18
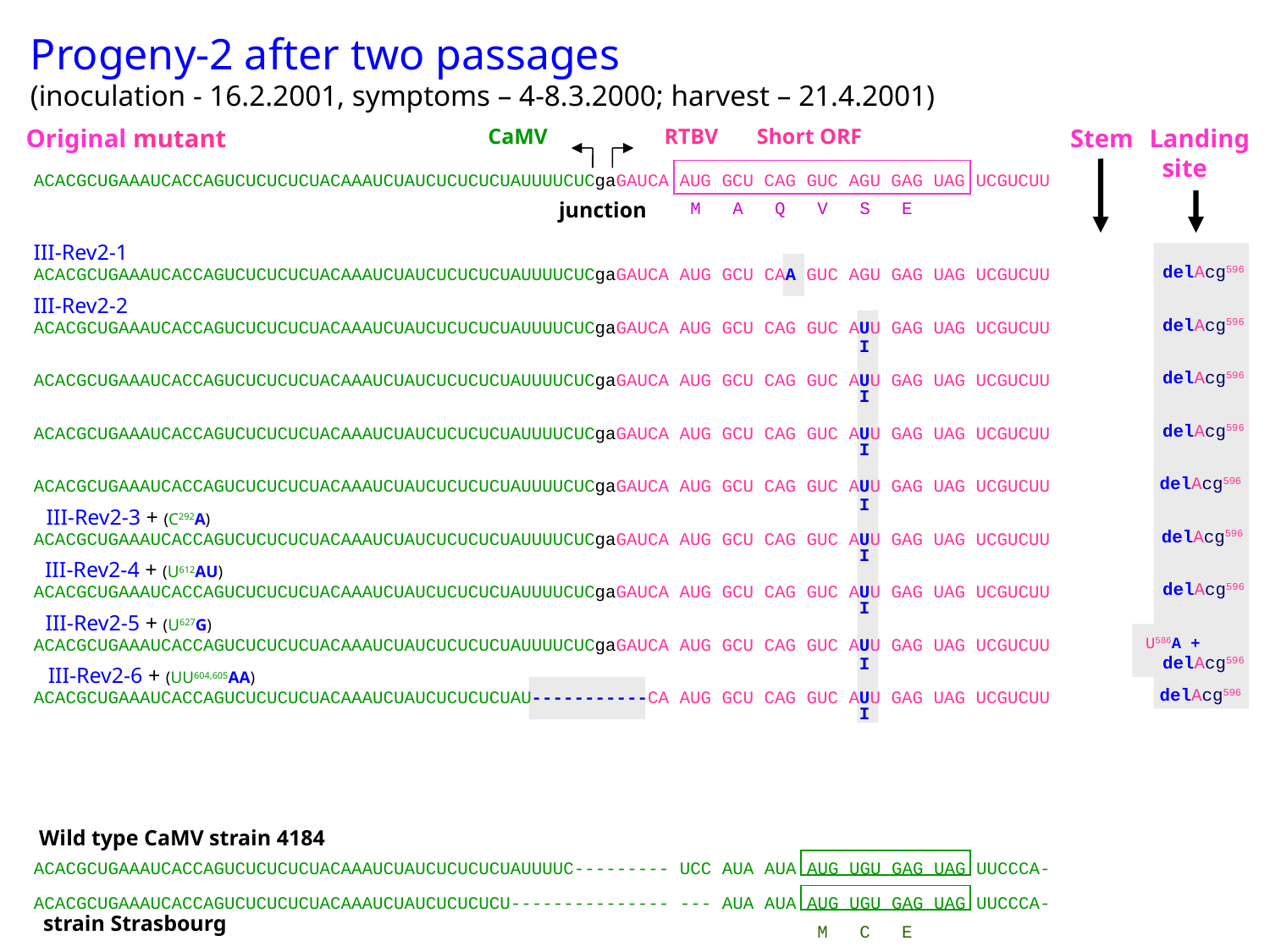

Progeny-2 after two passages
(inoculation - 16.2.2001, symptoms – 4-8.3.2000; harvest – 21.4.2001)
Original mutant
CaMV
RTBV Short ORF
Stem
Landing
 site
ACACGCUGAAAUCACCAGUCUCUCUCUACAAAUCUAUCUCUCUCUAUUUUCUCgaGAUCA AUG GCU CAG GUC AGU GAG UAG UCGUCUU
junction
M A Q V S E
III-Rev2-1
 delAcg596
ACACGCUGAAAUCACCAGUCUCUCUCUACAAAUCUAUCUCUCUCUAUUUUCUCgaGAUCA AUG GCU CAA GUC AGU GAG UAG UCGUCUU
III-Rev2-2
 delAcg596
ACACGCUGAAAUCACCAGUCUCUCUCUACAAAUCUAUCUCUCUCUAUUUUCUCgaGAUCA AUG GCU CAG GUC AUU GAG UAG UCGUCUU
I
 delAcg596
ACACGCUGAAAUCACCAGUCUCUCUCUACAAAUCUAUCUCUCUCUAUUUUCUCgaGAUCA AUG GCU CAG GUC AUU GAG UAG UCGUCUU
I
 delAcg596
ACACGCUGAAAUCACCAGUCUCUCUCUACAAAUCUAUCUCUCUCUAUUUUCUCgaGAUCA AUG GCU CAG GUC AUU GAG UAG UCGUCUU
I
 delAcg596
ACACGCUGAAAUCACCAGUCUCUCUCUACAAAUCUAUCUCUCUCUAUUUUCUCgaGAUCA AUG GCU CAG GUC AUU GAG UAG UCGUCUU
I
III-Rev2-3 + (C292A)
 delAcg596
ACACGCUGAAAUCACCAGUCUCUCUCUACAAAUCUAUCUCUCUCUAUUUUCUCgaGAUCA AUG GCU CAG GUC AUU GAG UAG UCGUCUU
I
III-Rev2-4 + (U612AU)
 delAcg596
ACACGCUGAAAUCACCAGUCUCUCUCUACAAAUCUAUCUCUCUCUAUUUUCUCgaGAUCA AUG GCU CAG GUC AUU GAG UAG UCGUCUU
I
III-Rev2-5 + (U627G)
 U586A +
 delAcg596
ACACGCUGAAAUCACCAGUCUCUCUCUACAAAUCUAUCUCUCUCUAUUUUCUCgaGAUCA AUG GCU CAG GUC AUU GAG UAG UCGUCUU
I
III-Rev2-6 + (UU604,605AA)
 delAcg596
ACACGCUGAAAUCACCAGUCUCUCUCUACAAAUCUAUCUCUCUCUAU-----------CA AUG GCU CAG GUC AUU GAG UAG UCGUCUU
I
Wild type CaMV strain 4184
ACACGCUGAAAUCACCAGUCUCUCUCUACAAAUCUAUCUCUCUCUAUUUUC--------- UCC AUA AUA AUG UGU GAG UAG UUCCCA-
ACACGCUGAAAUCACCAGUCUCUCUCUACAAAUCUAUCUCUCUCU--------------- --- AUA AUA AUG UGU GAG UAG UUCCCA-
strain Strasbourg
M C E

## Slide 19
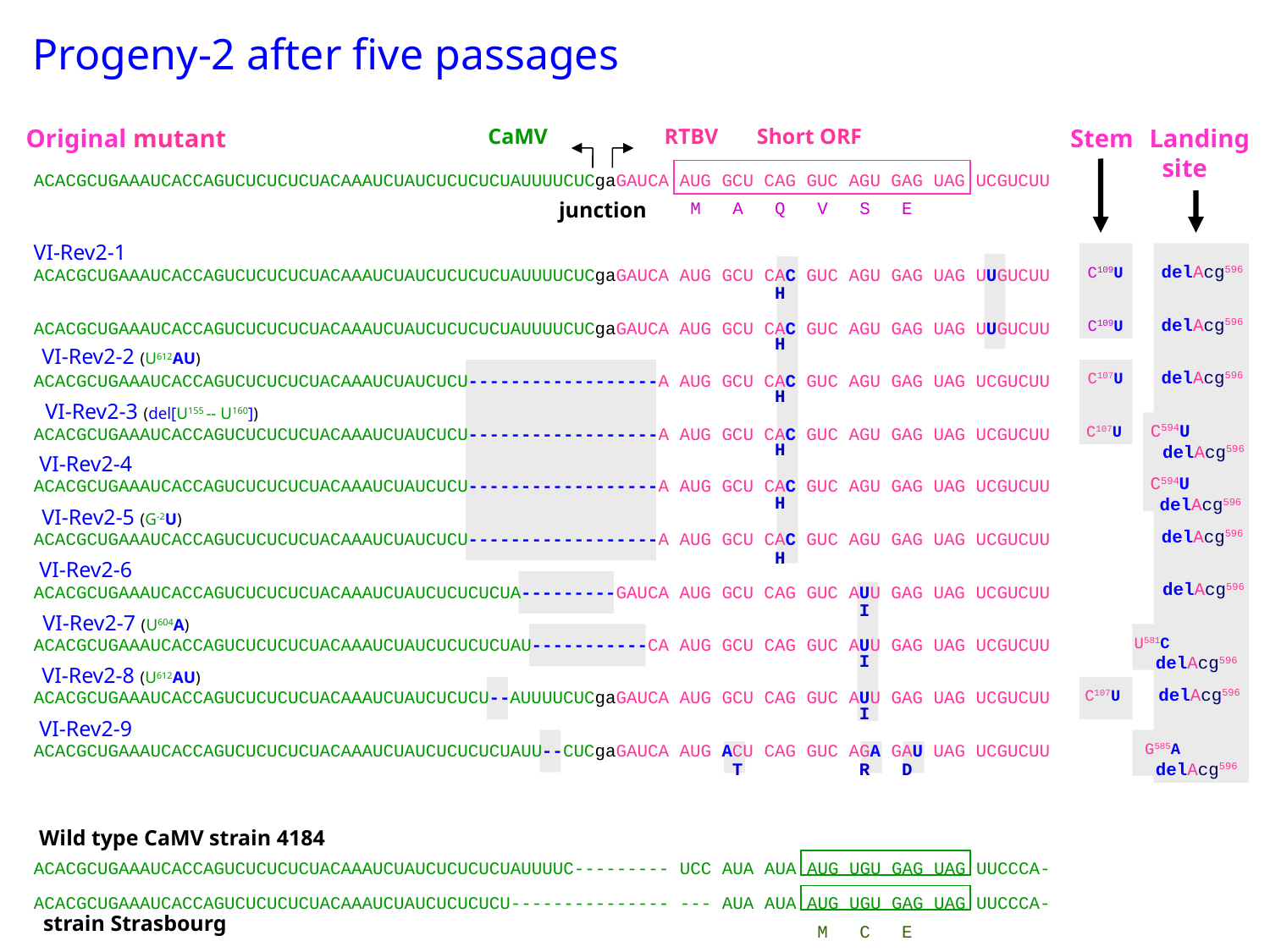

Progeny-2 after five passages
Original mutant
CaMV
RTBV Short ORF
Stem
Landing
 site
ACACGCUGAAAUCACCAGUCUCUCUCUACAAAUCUAUCUCUCUCUAUUUUCUCgaGAUCA AUG GCU CAG GUC AGU GAG UAG UCGUCUU
junction
M A Q V S E
VI-Rev2-1
ACACGCUGAAAUCACCAGUCUCUCUCUACAAAUCUAUCUCUCUCUAUUUUCUCgaGAUCA AUG GCU CAC GUC AGU GAG UAG UUGUCUU
C109U delAcg596
H
ACACGCUGAAAUCACCAGUCUCUCUCUACAAAUCUAUCUCUCUCUAUUUUCUCgaGAUCA AUG GCU CAC GUC AGU GAG UAG UUGUCUU
C109U delAcg596
H
VI-Rev2-2 (U612AU)
ACACGCUGAAAUCACCAGUCUCUCUCUACAAAUCUAUCUCU------------------A AUG GCU CAC GUC AGU GAG UAG UCGUCUU
C107U delAcg596
H
VI-Rev2-3 (del[U155 -- U160])
ACACGCUGAAAUCACCAGUCUCUCUCUACAAAUCUAUCUCU------------------A AUG GCU CAC GUC AGU GAG UAG UCGUCUU
C107U C594U
 delAcg596
H
VI-Rev2-4
 C594U
 delAcg596
ACACGCUGAAAUCACCAGUCUCUCUCUACAAAUCUAUCUCU------------------A AUG GCU CAC GUC AGU GAG UAG UCGUCUU
H
VI-Rev2-5 (G-2U)
 delAcg596
ACACGCUGAAAUCACCAGUCUCUCUCUACAAAUCUAUCUCU------------------A AUG GCU CAC GUC AGU GAG UAG UCGUCUU
H
VI-Rev2-6
ACACGCUGAAAUCACCAGUCUCUCUCUACAAAUCUAUCUCUCUCUA---------GAUCA AUG GCU CAG GUC AUU GAG UAG UCGUCUU
 delAcg596
I
VI-Rev2-7 (U604A)
 U581C
 delAcg596
ACACGCUGAAAUCACCAGUCUCUCUCUACAAAUCUAUCUCUCUCUAU-----------CA AUG GCU CAG GUC AUU GAG UAG UCGUCUU
I
VI-Rev2-8 (U612AU)
C107U delAcg596
ACACGCUGAAAUCACCAGUCUCUCUCUACAAAUCUAUCUCUCU--AUUUUCUCgaGAUCA AUG GCU CAG GUC AUU GAG UAG UCGUCUU
I
VI-Rev2-9
 G585A
 delAcg596
ACACGCUGAAAUCACCAGUCUCUCUCUACAAAUCUAUCUCUCUCUAUU--CUCgaGAUCA AUG ACU CAG GUC AGA GAU UAG UCGUCUU
T
R
D
Wild type CaMV strain 4184
ACACGCUGAAAUCACCAGUCUCUCUCUACAAAUCUAUCUCUCUCUAUUUUC--------- UCC AUA AUA AUG UGU GAG UAG UUCCCA-
ACACGCUGAAAUCACCAGUCUCUCUCUACAAAUCUAUCUCUCUCU--------------- --- AUA AUA AUG UGU GAG UAG UUCCCA-
strain Strasbourg
M C E

## Slide 20
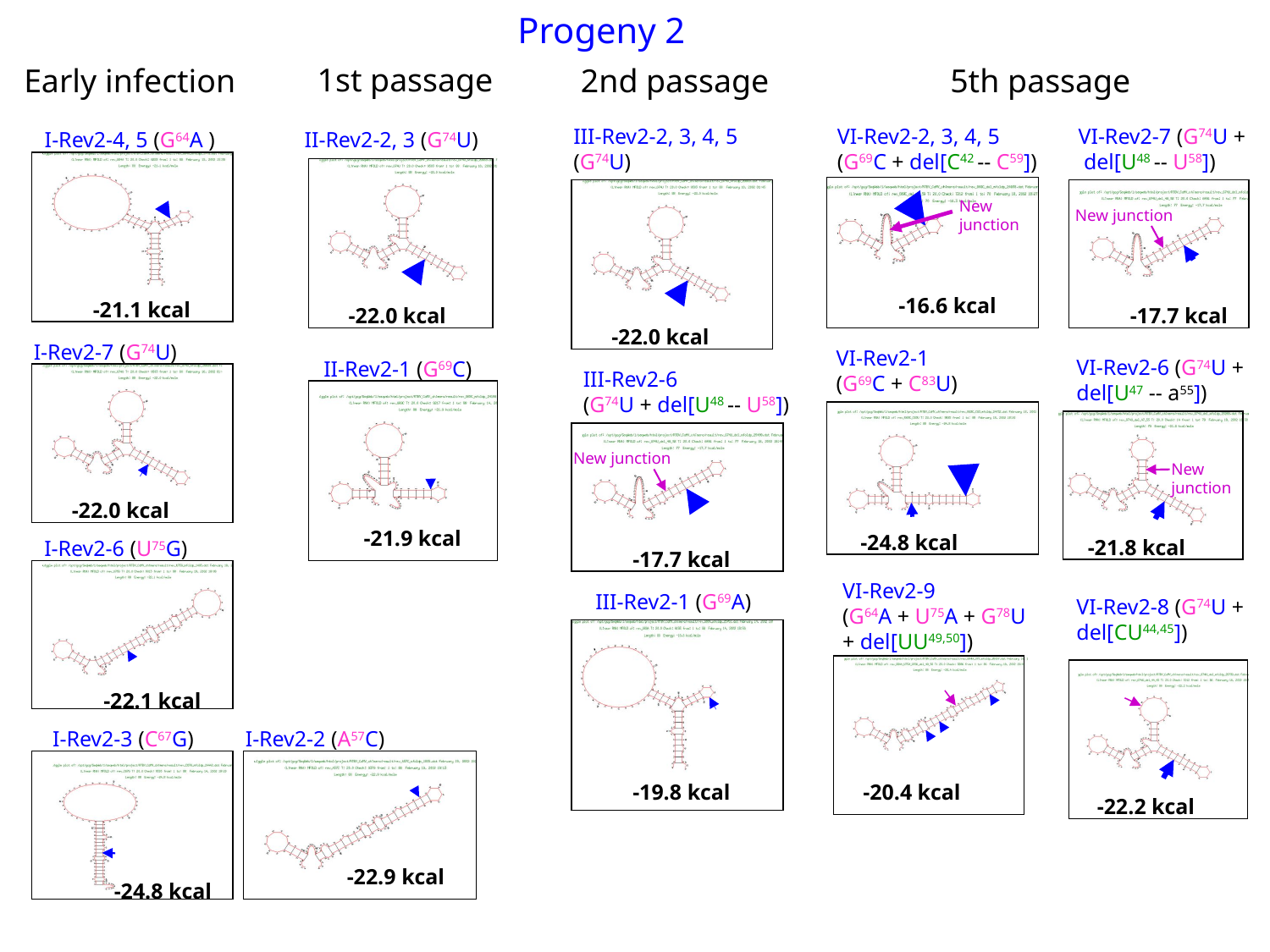

Progeny 2
1st passage
Early infection
2nd passage
5th passage
III-Rev2-2, 3, 4, 5
(G74U)
VI-Rev2-2, 3, 4, 5
(G69C + del[C42 -- C59])
VI-Rev2-7 (G74U +
 del[U48 -- U58])
I-Rev2-4, 5 (G64A )
II-Rev2-2, 3 (G74U)
New
junction
New junction
-16.6 kcal
-21.1 kcal
-22.0 kcal
-17.7 kcal
-22.0 kcal
I-Rev2-7 (G74U)
VI-Rev2-1
(G69C + C83U)
VI-Rev2-6 (G74U +
del[U47 -- a55])
II-Rev2-1 (G69C)
III-Rev2-6
(G74U + del[U48 -- U58])
New junction
New
junction
-22.0 kcal
-21.9 kcal
-24.8 kcal
-21.8 kcal
I-Rev2-6 (U75G)
-17.7 kcal
VI-Rev2-9
(G64A + U75A + G78U
+ del[UU49,50])
III-Rev2-1 (G69A)
VI-Rev2-8 (G74U +
del[CU44,45])
-22.1 kcal
I-Rev2-3 (C67G)
I-Rev2-2 (A57C)
-19.8 kcal
-20.4 kcal
-22.2 kcal
-22.9 kcal
-24.8 kcal

## Slide 21
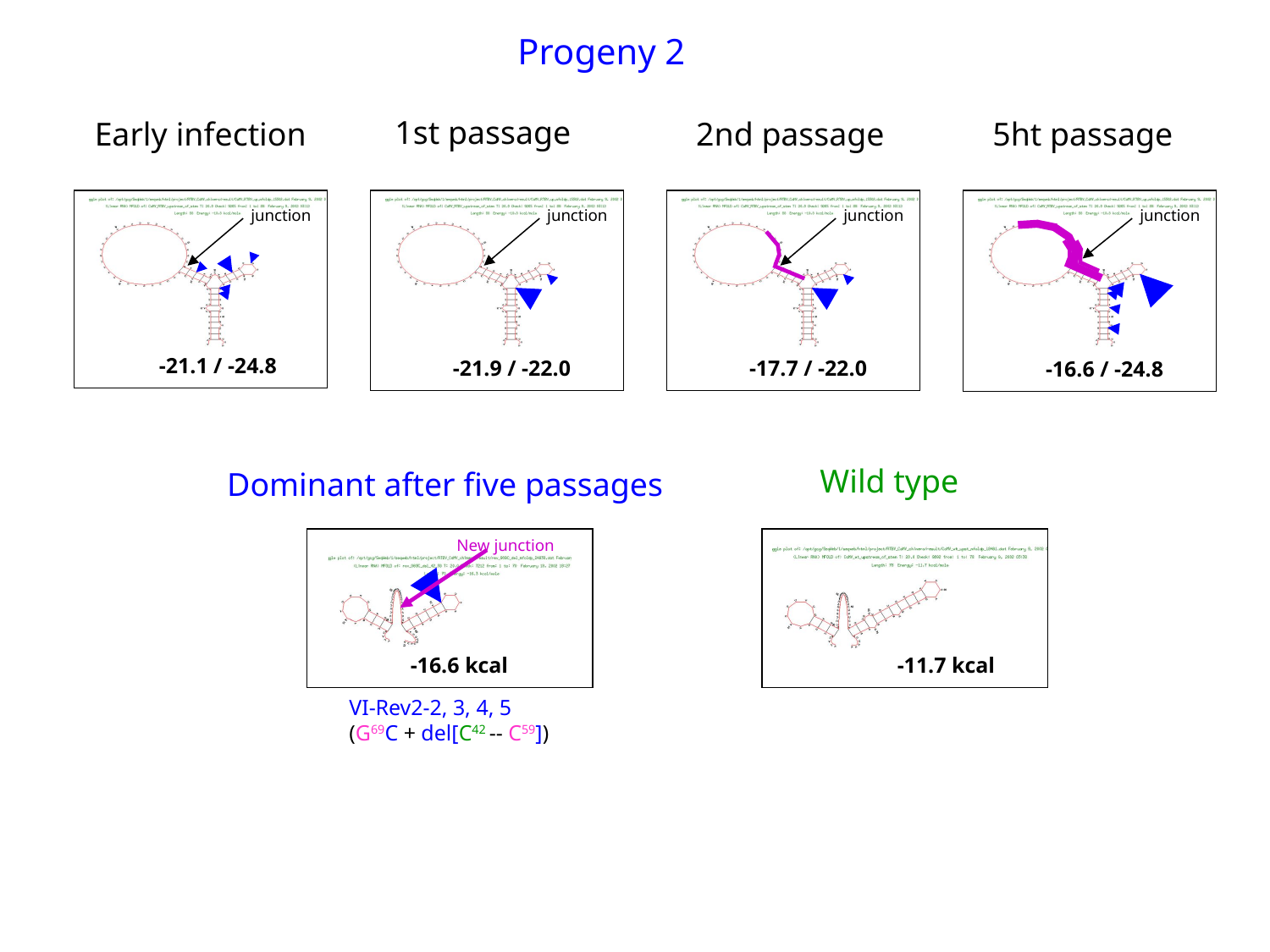

Progeny 2
1st passage
Early infection
2nd passage
5ht passage
junction
junction
junction
junction
-21.1 / -24.8
-21.9 / -22.0
-17.7 / -22.0
-16.6 / -24.8
Wild type
Dominant after five passages
New junction
-16.6 kcal
-11.7 kcal
VI-Rev2-2, 3, 4, 5
(G69C + del[C42 -- C59])

## Slide 22
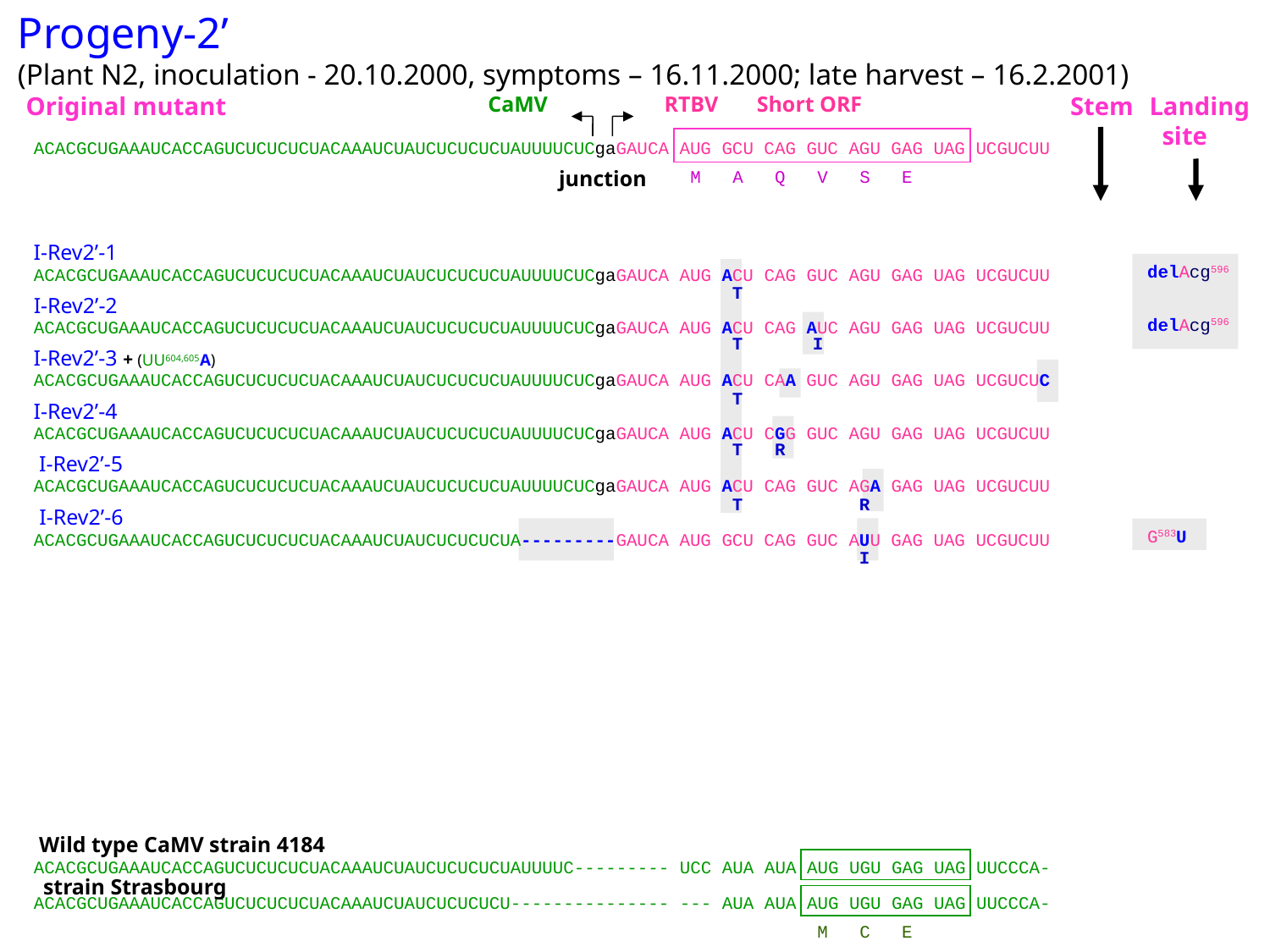

Progeny-2’
(Plant N2, inoculation - 20.10.2000, symptoms – 16.11.2000; late harvest – 16.2.2001)
Original mutant
CaMV
RTBV Short ORF
Stem
Landing
 site
ACACGCUGAAAUCACCAGUCUCUCUCUACAAAUCUAUCUCUCUCUAUUUUCUCgaGAUCA AUG GCU CAG GUC AGU GAG UAG UCGUCUU
junction
M A Q V S E
I-Rev2’-1
ACACGCUGAAAUCACCAGUCUCUCUCUACAAAUCUAUCUCUCUCUAUUUUCUCgaGAUCA AUG ACU CAG GUC AGU GAG UAG UCGUCUU
 delAcg596
T
I-Rev2’-2
 delAcg596
ACACGCUGAAAUCACCAGUCUCUCUCUACAAAUCUAUCUCUCUCUAUUUUCUCgaGAUCA AUG ACU CAG AUC AGU GAG UAG UCGUCUU
T
I
I-Rev2’-3 + (UU604,605A)
ACACGCUGAAAUCACCAGUCUCUCUCUACAAAUCUAUCUCUCUCUAUUUUCUCgaGAUCA AUG ACU CAA GUC AGU GAG UAG UCGUCUC
T
I-Rev2’-4
ACACGCUGAAAUCACCAGUCUCUCUCUACAAAUCUAUCUCUCUCUAUUUUCUCgaGAUCA AUG ACU CGG GUC AGU GAG UAG UCGUCUU
T
R
I-Rev2’-5
ACACGCUGAAAUCACCAGUCUCUCUCUACAAAUCUAUCUCUCUCUAUUUUCUCgaGAUCA AUG ACU CAG GUC AGA GAG UAG UCGUCUU
T
R
I-Rev2’-6
ACACGCUGAAAUCACCAGUCUCUCUCUACAAAUCUAUCUCUCUCUA---------GAUCA AUG GCU CAG GUC AUU GAG UAG UCGUCUU
 G583U
I
Wild type CaMV strain 4184
ACACGCUGAAAUCACCAGUCUCUCUCUACAAAUCUAUCUCUCUCUAUUUUC--------- UCC AUA AUA AUG UGU GAG UAG UUCCCA-
strain Strasbourg
ACACGCUGAAAUCACCAGUCUCUCUCUACAAAUCUAUCUCUCUCU--------------- --- AUA AUA AUG UGU GAG UAG UUCCCA-
M C E

## Slide 23
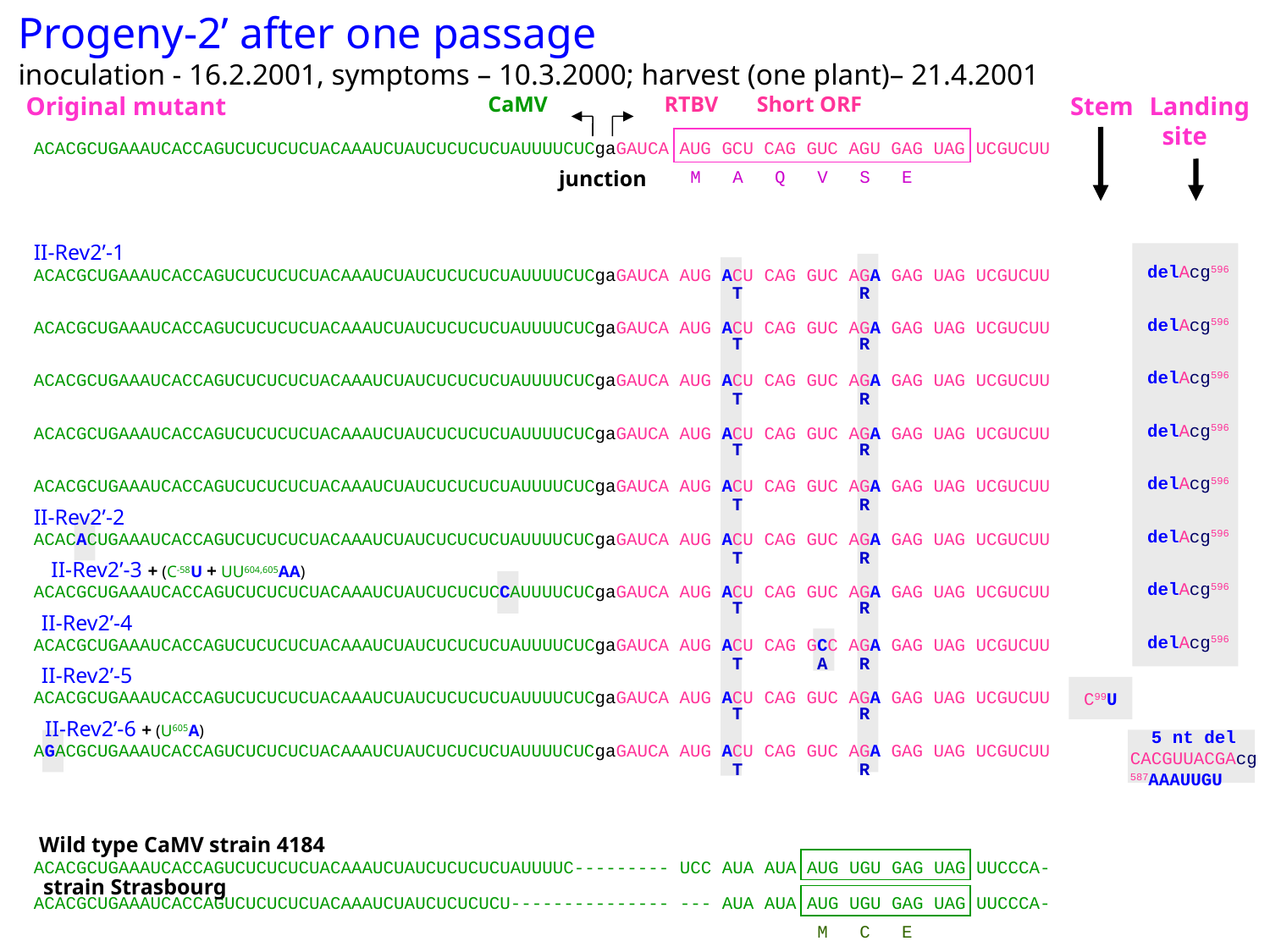

Progeny-2’ after one passage
inoculation - 16.2.2001, symptoms – 10.3.2000; harvest (one plant)– 21.4.2001
Original mutant
CaMV
RTBV Short ORF
Stem
Landing
 site
ACACGCUGAAAUCACCAGUCUCUCUCUACAAAUCUAUCUCUCUCUAUUUUCUCgaGAUCA AUG GCU CAG GUC AGU GAG UAG UCGUCUU
junction
M A Q V S E
II-Rev2’-1
ACACGCUGAAAUCACCAGUCUCUCUCUACAAAUCUAUCUCUCUCUAUUUUCUCgaGAUCA AUG ACU CAG GUC AGA GAG UAG UCGUCUU
 delAcg596
T
R
 delAcg596
ACACGCUGAAAUCACCAGUCUCUCUCUACAAAUCUAUCUCUCUCUAUUUUCUCgaGAUCA AUG ACU CAG GUC AGA GAG UAG UCGUCUU
T
R
 delAcg596
ACACGCUGAAAUCACCAGUCUCUCUCUACAAAUCUAUCUCUCUCUAUUUUCUCgaGAUCA AUG ACU CAG GUC AGA GAG UAG UCGUCUU
T
R
 delAcg596
ACACGCUGAAAUCACCAGUCUCUCUCUACAAAUCUAUCUCUCUCUAUUUUCUCgaGAUCA AUG ACU CAG GUC AGA GAG UAG UCGUCUU
T
R
 delAcg596
ACACGCUGAAAUCACCAGUCUCUCUCUACAAAUCUAUCUCUCUCUAUUUUCUCgaGAUCA AUG ACU CAG GUC AGA GAG UAG UCGUCUU
T
R
II-Rev2’-2
 delAcg596
ACACACUGAAAUCACCAGUCUCUCUCUACAAAUCUAUCUCUCUCUAUUUUCUCgaGAUCA AUG ACU CAG GUC AGA GAG UAG UCGUCUU
T
R
II-Rev2’-3 + (C-58U + UU604,605AA)
 delAcg596
ACACGCUGAAAUCACCAGUCUCUCUCUACAAAUCUAUCUCUCUCCAUUUUCUCgaGAUCA AUG ACU CAG GUC AGA GAG UAG UCGUCUU
T
R
II-Rev2’-4
 delAcg596
ACACGCUGAAAUCACCAGUCUCUCUCUACAAAUCUAUCUCUCUCUAUUUUCUCgaGAUCA AUG ACU CAG GCC AGA GAG UAG UCGUCUU
T
A
R
II-Rev2’-5
C99U
ACACGCUGAAAUCACCAGUCUCUCUCUACAAAUCUAUCUCUCUCUAUUUUCUCgaGAUCA AUG ACU CAG GUC AGA GAG UAG UCGUCUU
T
R
II-Rev2’-6 + (U605A)
 5 nt del
 CACGUUACGAcg
 587AAAUUGU
AGACGCUGAAAUCACCAGUCUCUCUCUACAAAUCUAUCUCUCUCUAUUUUCUCgaGAUCA AUG ACU CAG GUC AGA GAG UAG UCGUCUU
T
R
Wild type CaMV strain 4184
ACACGCUGAAAUCACCAGUCUCUCUCUACAAAUCUAUCUCUCUCUAUUUUC--------- UCC AUA AUA AUG UGU GAG UAG UUCCCA-
strain Strasbourg
ACACGCUGAAAUCACCAGUCUCUCUCUACAAAUCUAUCUCUCUCU--------------- --- AUA AUA AUG UGU GAG UAG UUCCCA-
M C E

## Slide 24
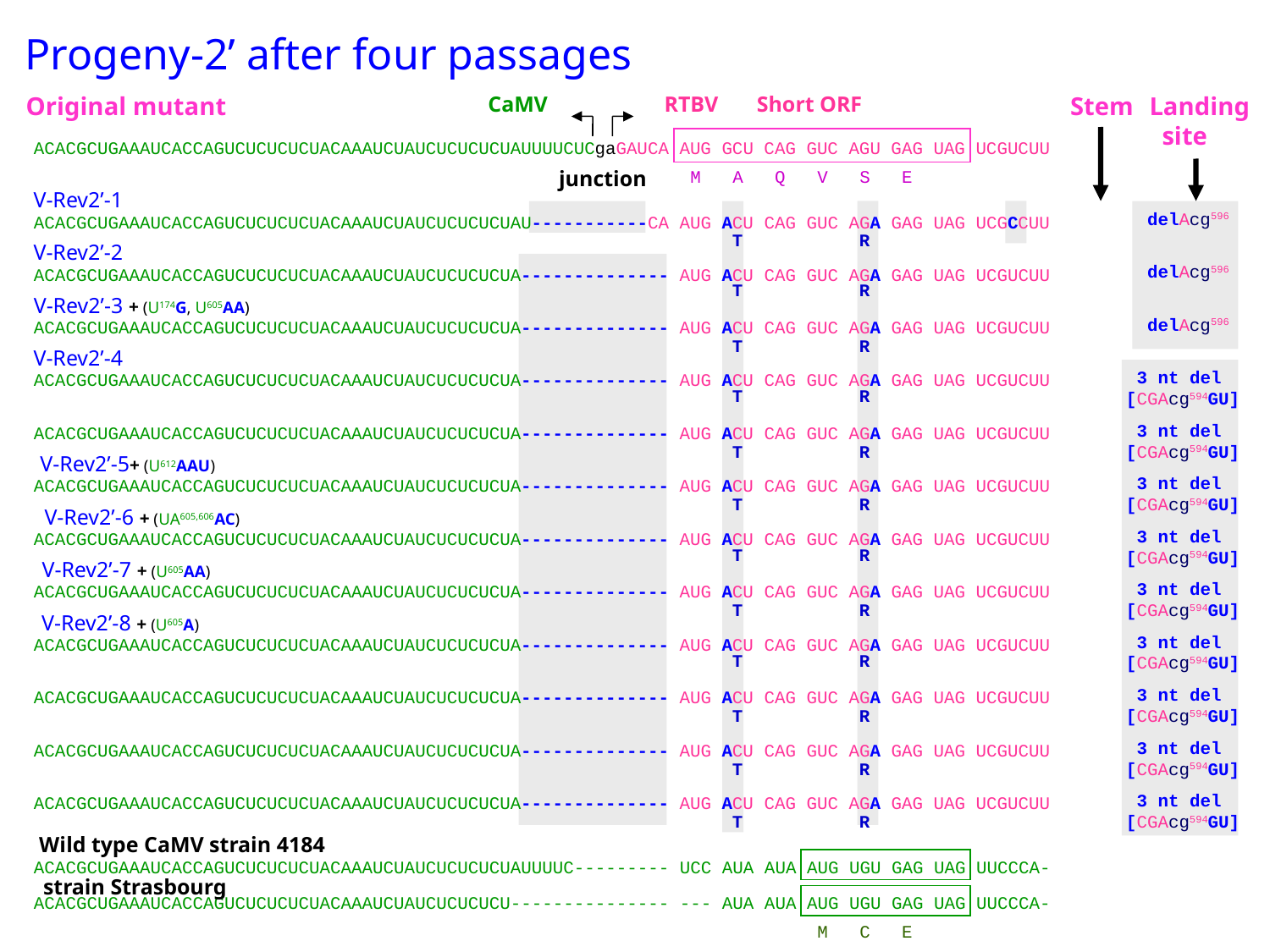

Progeny-2’ after four passages
Original mutant
CaMV
RTBV Short ORF
Stem
Landing
 site
ACACGCUGAAAUCACCAGUCUCUCUCUACAAAUCUAUCUCUCUCUAUUUUCUCgaGAUCA AUG GCU CAG GUC AGU GAG UAG UCGUCUU
junction
M A Q V S E
V-Rev2’-1
ACACGCUGAAAUCACCAGUCUCUCUCUACAAAUCUAUCUCUCUCUAU-----------CA AUG ACU CAG GUC AGA GAG UAG UCGCCUU
 delAcg596
T
R
V-Rev2’-2
ACACGCUGAAAUCACCAGUCUCUCUCUACAAAUCUAUCUCUCUCUA-------------- AUG ACU CAG GUC AGA GAG UAG UCGUCUU
 delAcg596
T
R
V-Rev2’-3 + (U174G, U605AA)
 delAcg596
ACACGCUGAAAUCACCAGUCUCUCUCUACAAAUCUAUCUCUCUCUA-------------- AUG ACU CAG GUC AGA GAG UAG UCGUCUU
T
R
V-Rev2’-4
 3 nt del
 [CGAcg594GU]
ACACGCUGAAAUCACCAGUCUCUCUCUACAAAUCUAUCUCUCUCUA-------------- AUG ACU CAG GUC AGA GAG UAG UCGUCUU
T
R
 3 nt del
 [CGAcg594GU]
ACACGCUGAAAUCACCAGUCUCUCUCUACAAAUCUAUCUCUCUCUA-------------- AUG ACU CAG GUC AGA GAG UAG UCGUCUU
T
R
V-Rev2’-5+ (U612AAU)
 3 nt del
 [CGAcg594GU]
ACACGCUGAAAUCACCAGUCUCUCUCUACAAAUCUAUCUCUCUCUA-------------- AUG ACU CAG GUC AGA GAG UAG UCGUCUU
T
R
V-Rev2’-6 + (UA605,606AC)
 3 nt del
 [CGAcg594GU]
ACACGCUGAAAUCACCAGUCUCUCUCUACAAAUCUAUCUCUCUCUA-------------- AUG ACU CAG GUC AGA GAG UAG UCGUCUU
T
R
V-Rev2’-7 + (U605AA)
 3 nt del
 [CGAcg594GU]
ACACGCUGAAAUCACCAGUCUCUCUCUACAAAUCUAUCUCUCUCUA-------------- AUG ACU CAG GUC AGA GAG UAG UCGUCUU
T
R
V-Rev2’-8 + (U605A)
 3 nt del
 [CGAcg594GU]
ACACGCUGAAAUCACCAGUCUCUCUCUACAAAUCUAUCUCUCUCUA-------------- AUG ACU CAG GUC AGA GAG UAG UCGUCUU
T
R
 3 nt del
 [CGAcg594GU]
ACACGCUGAAAUCACCAGUCUCUCUCUACAAAUCUAUCUCUCUCUA-------------- AUG ACU CAG GUC AGA GAG UAG UCGUCUU
T
R
 3 nt del
 [CGAcg594GU]
ACACGCUGAAAUCACCAGUCUCUCUCUACAAAUCUAUCUCUCUCUA-------------- AUG ACU CAG GUC AGA GAG UAG UCGUCUU
T
R
 3 nt del
 [CGAcg594GU]
ACACGCUGAAAUCACCAGUCUCUCUCUACAAAUCUAUCUCUCUCUA-------------- AUG ACU CAG GUC AGA GAG UAG UCGUCUU
T
R
Wild type CaMV strain 4184
ACACGCUGAAAUCACCAGUCUCUCUCUACAAAUCUAUCUCUCUCUAUUUUC--------- UCC AUA AUA AUG UGU GAG UAG UUCCCA-
strain Strasbourg
ACACGCUGAAAUCACCAGUCUCUCUCUACAAAUCUAUCUCUCUCU--------------- --- AUA AUA AUG UGU GAG UAG UUCCCA-
M C E

## Slide 25
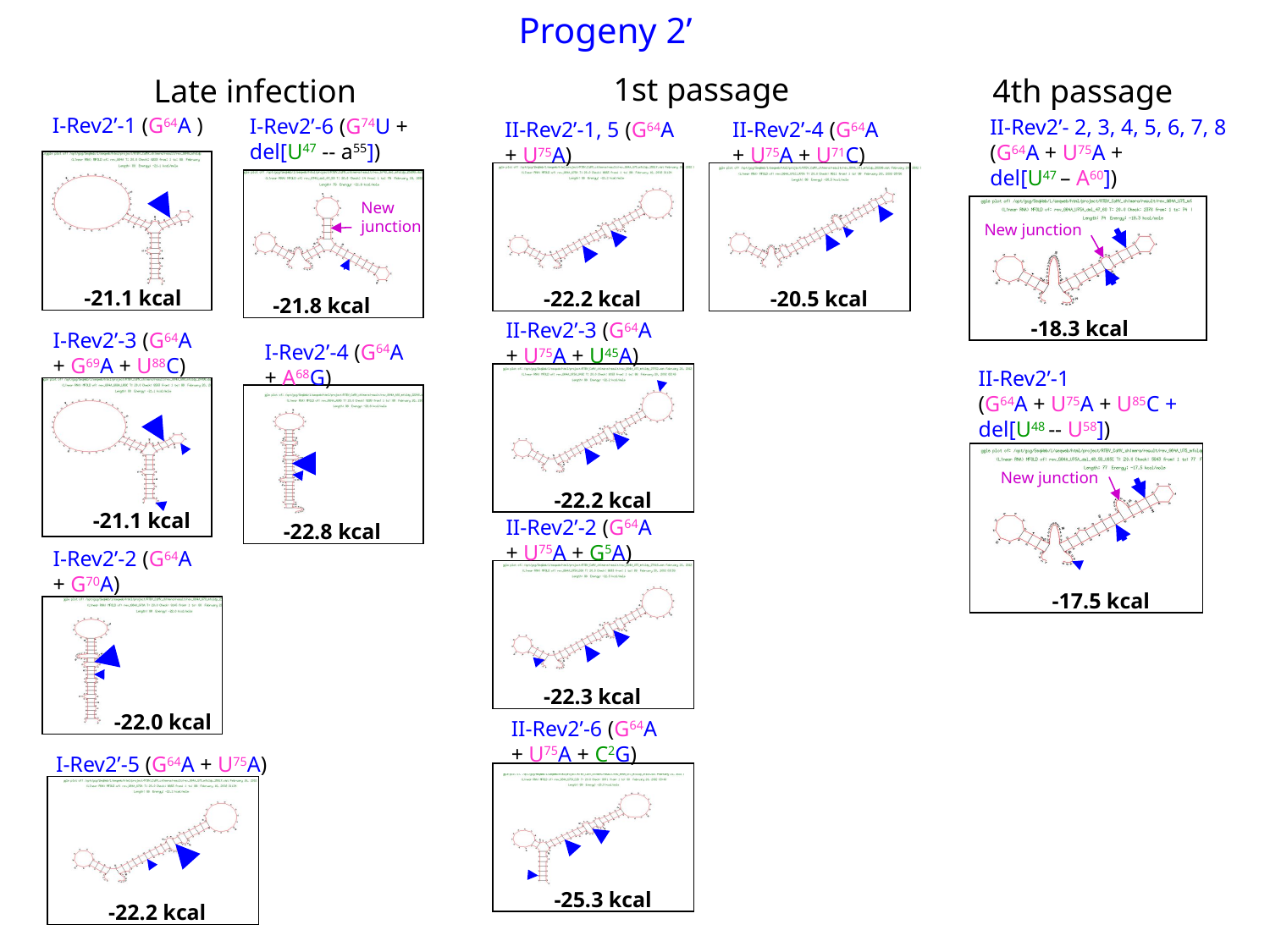

Progeny 2’
1st passage
Late infection
4th passage
I-Rev2’-1 (G64A )
I-Rev2’-6 (G74U +
del[U47 -- a55])
II-Rev2’- 2, 3, 4, 5, 6, 7, 8
(G64A + U75A +
del[U47 – A60])
II-Rev2’-1, 5 (G64A
+ U75A)
II-Rev2’-4 (G64A
+ U75A + U71C)
New
junction
New junction
-21.1 kcal
-22.2 kcal
-20.5 kcal
-21.8 kcal
-18.3 kcal
II-Rev2’-3 (G64A
+ U75A + U45A)
I-Rev2’-3 (G64A
+ G69A + U88C)
I-Rev2’-4 (G64A
+ A68G)
II-Rev2’-1
(G64A + U75A + U85C +
del[U48 -- U58])
New junction
-22.2 kcal
-21.1 kcal
II-Rev2’-2 (G64A
+ U75A + G5A)
-22.8 kcal
I-Rev2’-2 (G64A
+ G70A)
-17.5 kcal
-22.3 kcal
-22.0 kcal
II-Rev2’-6 (G64A
+ U75A + C2G)
I-Rev2’-5 (G64A + U75A)
-25.3 kcal
-22.2 kcal

## Slide 26
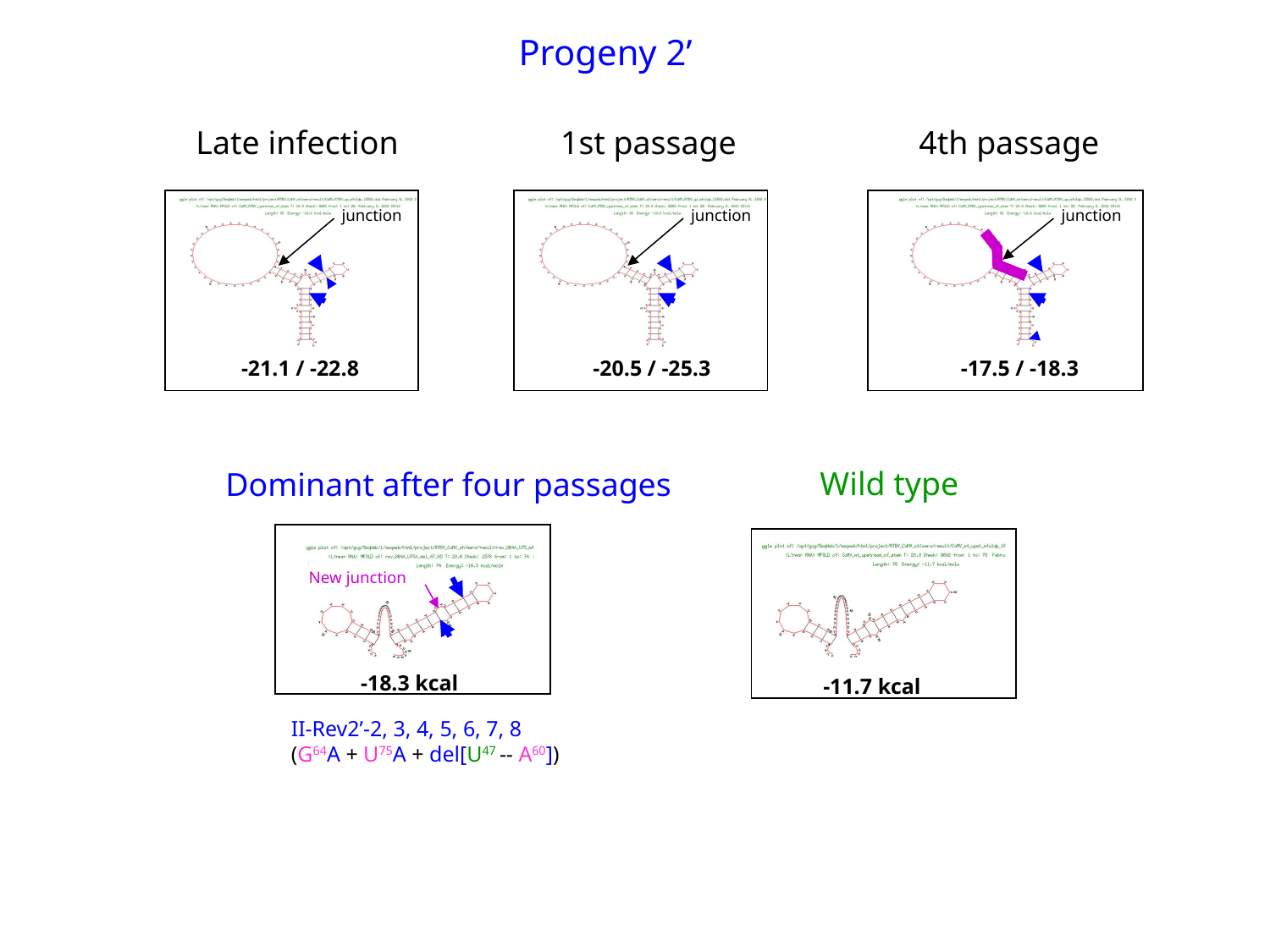

Progeny 2’
Late infection
1st passage
4th passage
junction
junction
junction
-21.1 / -22.8
-20.5 / -25.3
-17.5 / -18.3
Wild type
Dominant after four passages
New junction
-18.3 kcal
-11.7 kcal
II-Rev2’-2, 3, 4, 5, 6, 7, 8
(G64A + U75A + del[U47 -- A60])

## Slide 27
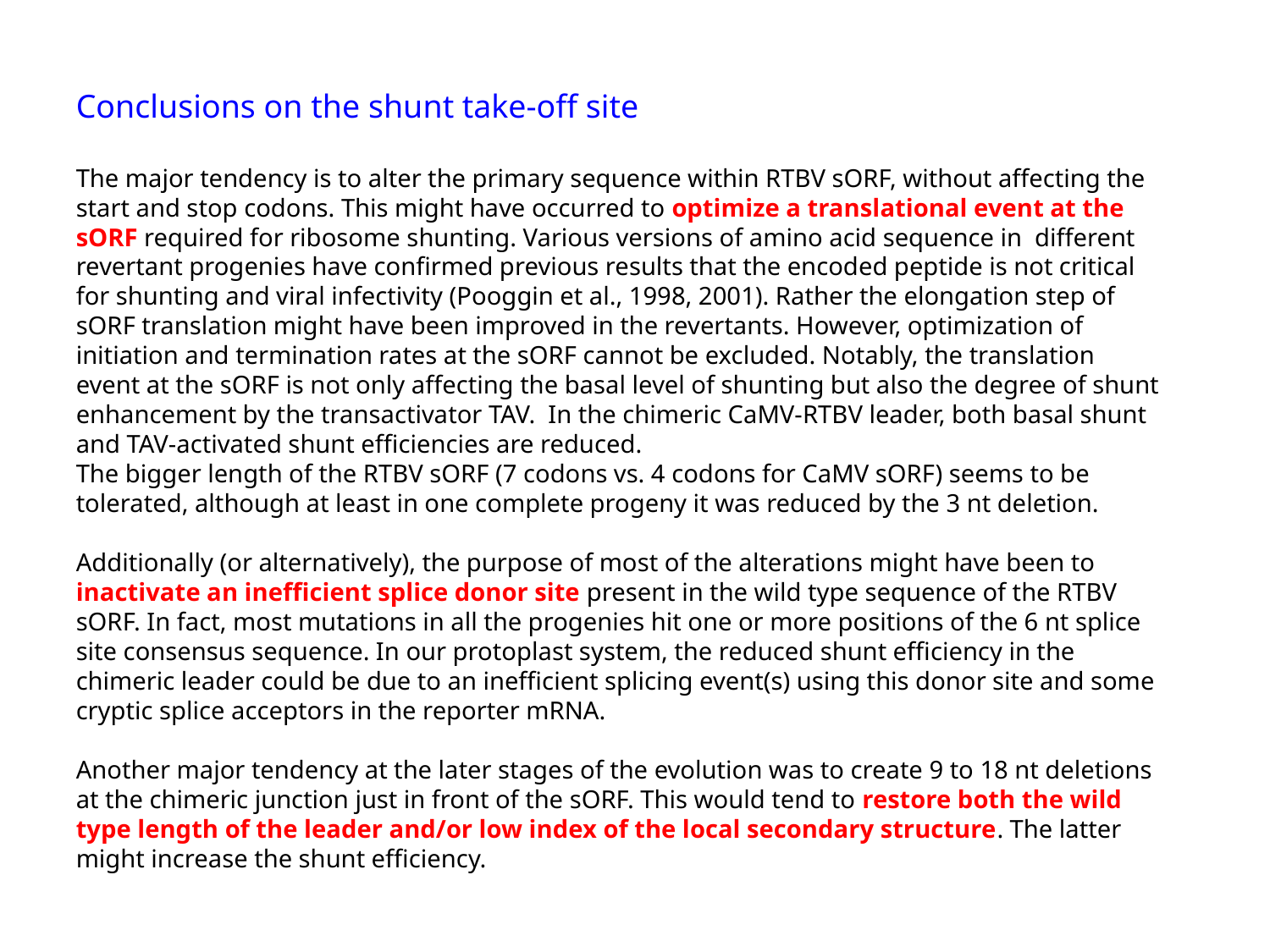

Conclusions on the shunt take-off site
The major tendency is to alter the primary sequence within RTBV sORF, without affecting the start and stop codons. This might have occurred to optimize a translational event at the sORF required for ribosome shunting. Various versions of amino acid sequence in different revertant progenies have confirmed previous results that the encoded peptide is not critical for shunting and viral infectivity (Pooggin et al., 1998, 2001). Rather the elongation step of sORF translation might have been improved in the revertants. However, optimization of initiation and termination rates at the sORF cannot be excluded. Notably, the translation event at the sORF is not only affecting the basal level of shunting but also the degree of shunt enhancement by the transactivator TAV. In the chimeric CaMV-RTBV leader, both basal shunt and TAV-activated shunt efficiencies are reduced.
The bigger length of the RTBV sORF (7 codons vs. 4 codons for CaMV sORF) seems to be tolerated, although at least in one complete progeny it was reduced by the 3 nt deletion.
Additionally (or alternatively), the purpose of most of the alterations might have been to inactivate an inefficient splice donor site present in the wild type sequence of the RTBV sORF. In fact, most mutations in all the progenies hit one or more positions of the 6 nt splice site consensus sequence. In our protoplast system, the reduced shunt efficiency in the chimeric leader could be due to an inefficient splicing event(s) using this donor site and some cryptic splice acceptors in the reporter mRNA.
Another major tendency at the later stages of the evolution was to create 9 to 18 nt deletions at the chimeric junction just in front of the sORF. This would tend to restore both the wild type length of the leader and/or low index of the local secondary structure. The latter might increase the shunt efficiency.

## Slide 28
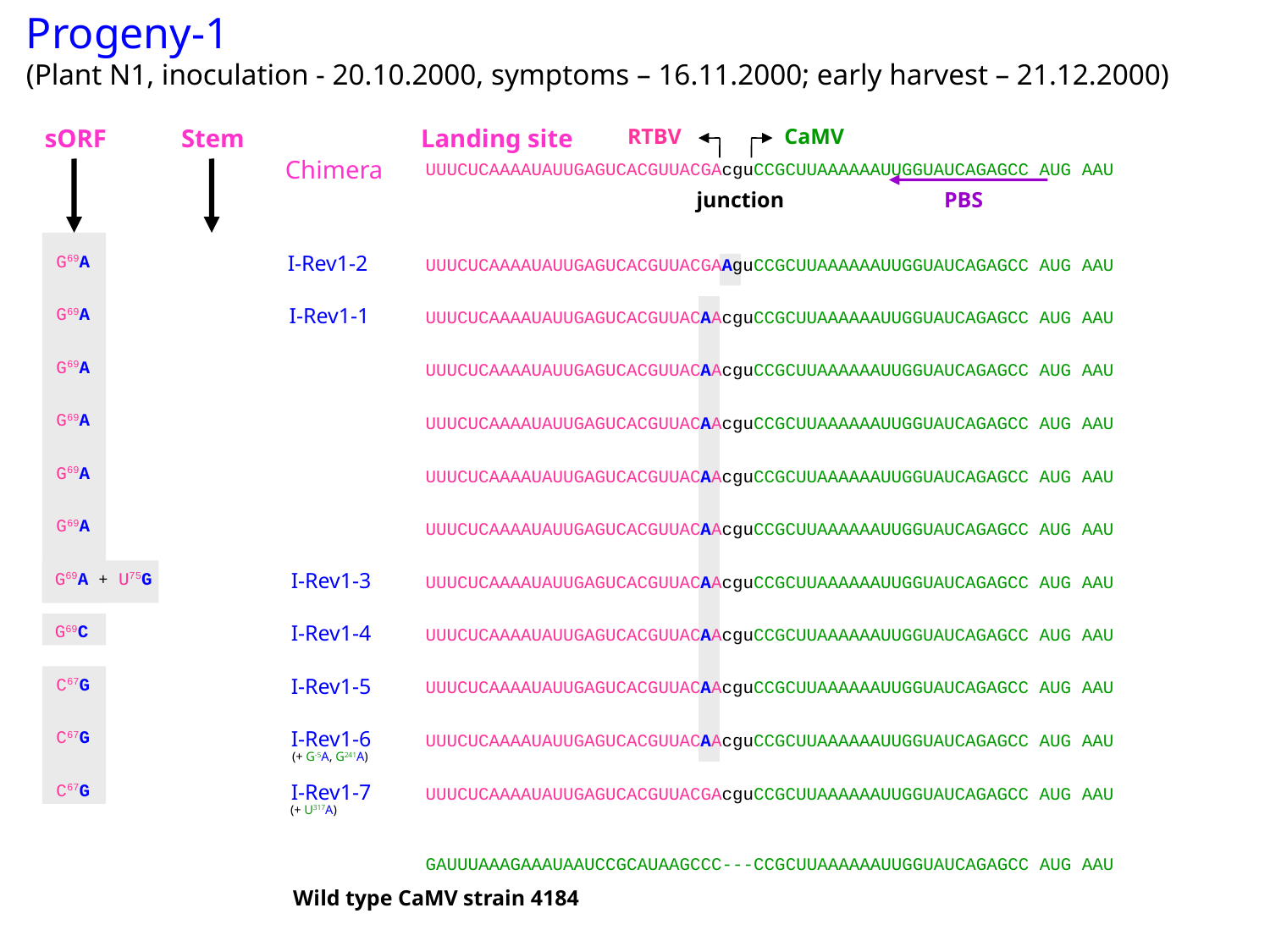

Progeny-1
(Plant N1, inoculation - 20.10.2000, symptoms – 16.11.2000; early harvest – 21.12.2000)
sORF
Stem
Landing site
RTBV
CaMV
Chimera
UUUCUCAAAAUAUUGAGUCACGUUACGAcguCCGCUUAAAAAAUUGGUAUCAGAGCC AUG AAU
PBS
junction
G69A
I-Rev1-2
UUUCUCAAAAUAUUGAGUCACGUUACGAAguCCGCUUAAAAAAUUGGUAUCAGAGCC AUG AAU
G69A
I-Rev1-1
UUUCUCAAAAUAUUGAGUCACGUUACAAcguCCGCUUAAAAAAUUGGUAUCAGAGCC AUG AAU
G69A
UUUCUCAAAAUAUUGAGUCACGUUACAAcguCCGCUUAAAAAAUUGGUAUCAGAGCC AUG AAU
G69A
UUUCUCAAAAUAUUGAGUCACGUUACAAcguCCGCUUAAAAAAUUGGUAUCAGAGCC AUG AAU
G69A
UUUCUCAAAAUAUUGAGUCACGUUACAAcguCCGCUUAAAAAAUUGGUAUCAGAGCC AUG AAU
G69A
UUUCUCAAAAUAUUGAGUCACGUUACAAcguCCGCUUAAAAAAUUGGUAUCAGAGCC AUG AAU
G69A + U75G
I-Rev1-3
UUUCUCAAAAUAUUGAGUCACGUUACAAcguCCGCUUAAAAAAUUGGUAUCAGAGCC AUG AAU
G69C
I-Rev1-4
UUUCUCAAAAUAUUGAGUCACGUUACAAcguCCGCUUAAAAAAUUGGUAUCAGAGCC AUG AAU
C67G
I-Rev1-5
UUUCUCAAAAUAUUGAGUCACGUUACAAcguCCGCUUAAAAAAUUGGUAUCAGAGCC AUG AAU
C67G
I-Rev1-6
UUUCUCAAAAUAUUGAGUCACGUUACAAcguCCGCUUAAAAAAUUGGUAUCAGAGCC AUG AAU
(+ G-5A, G241A)
C67G
I-Rev1-7
UUUCUCAAAAUAUUGAGUCACGUUACGAcguCCGCUUAAAAAAUUGGUAUCAGAGCC AUG AAU
(+ U317A)
GAUUUAAAGAAAUAAUCCGCAUAAGCCC---CCGCUUAAAAAAUUGGUAUCAGAGCC AUG AAU
Wild type CaMV strain 4184

## Slide 29
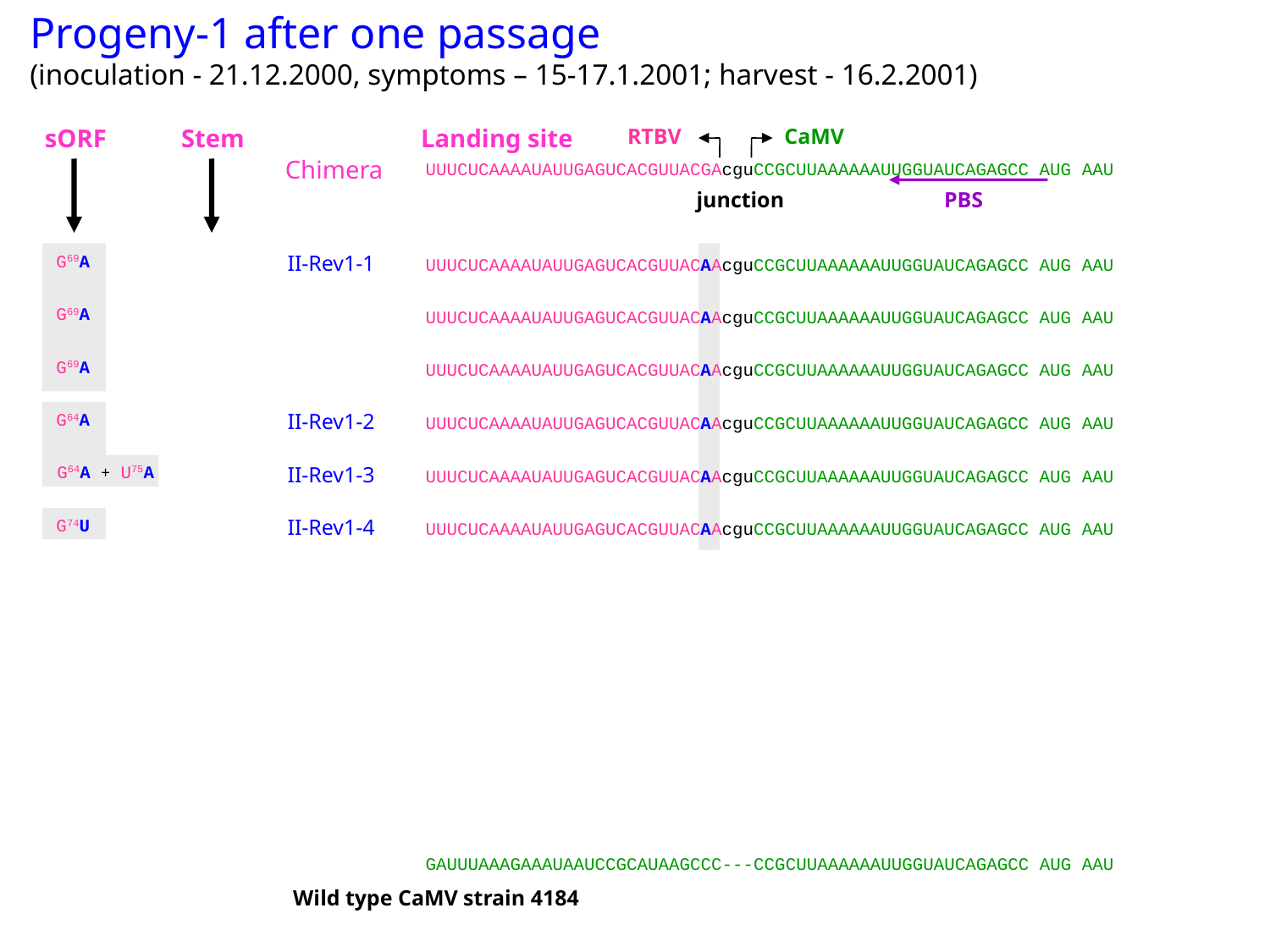

Progeny-1 after one passage
(inoculation - 21.12.2000, symptoms – 15-17.1.2001; harvest - 16.2.2001)
sORF
Stem
Landing site
RTBV
CaMV
Chimera
UUUCUCAAAAUAUUGAGUCACGUUACGAcguCCGCUUAAAAAAUUGGUAUCAGAGCC AUG AAU
PBS
junction
G69A
II-Rev1-1
UUUCUCAAAAUAUUGAGUCACGUUACAAcguCCGCUUAAAAAAUUGGUAUCAGAGCC AUG AAU
G69A
UUUCUCAAAAUAUUGAGUCACGUUACAAcguCCGCUUAAAAAAUUGGUAUCAGAGCC AUG AAU
G69A
UUUCUCAAAAUAUUGAGUCACGUUACAAcguCCGCUUAAAAAAUUGGUAUCAGAGCC AUG AAU
G64A
II-Rev1-2
UUUCUCAAAAUAUUGAGUCACGUUACAAcguCCGCUUAAAAAAUUGGUAUCAGAGCC AUG AAU
G64A + U75A
II-Rev1-3
UUUCUCAAAAUAUUGAGUCACGUUACAAcguCCGCUUAAAAAAUUGGUAUCAGAGCC AUG AAU
G74U
II-Rev1-4
UUUCUCAAAAUAUUGAGUCACGUUACAAcguCCGCUUAAAAAAUUGGUAUCAGAGCC AUG AAU
GAUUUAAAGAAAUAAUCCGCAUAAGCCC---CCGCUUAAAAAAUUGGUAUCAGAGCC AUG AAU
Wild type CaMV strain 4184

## Slide 30
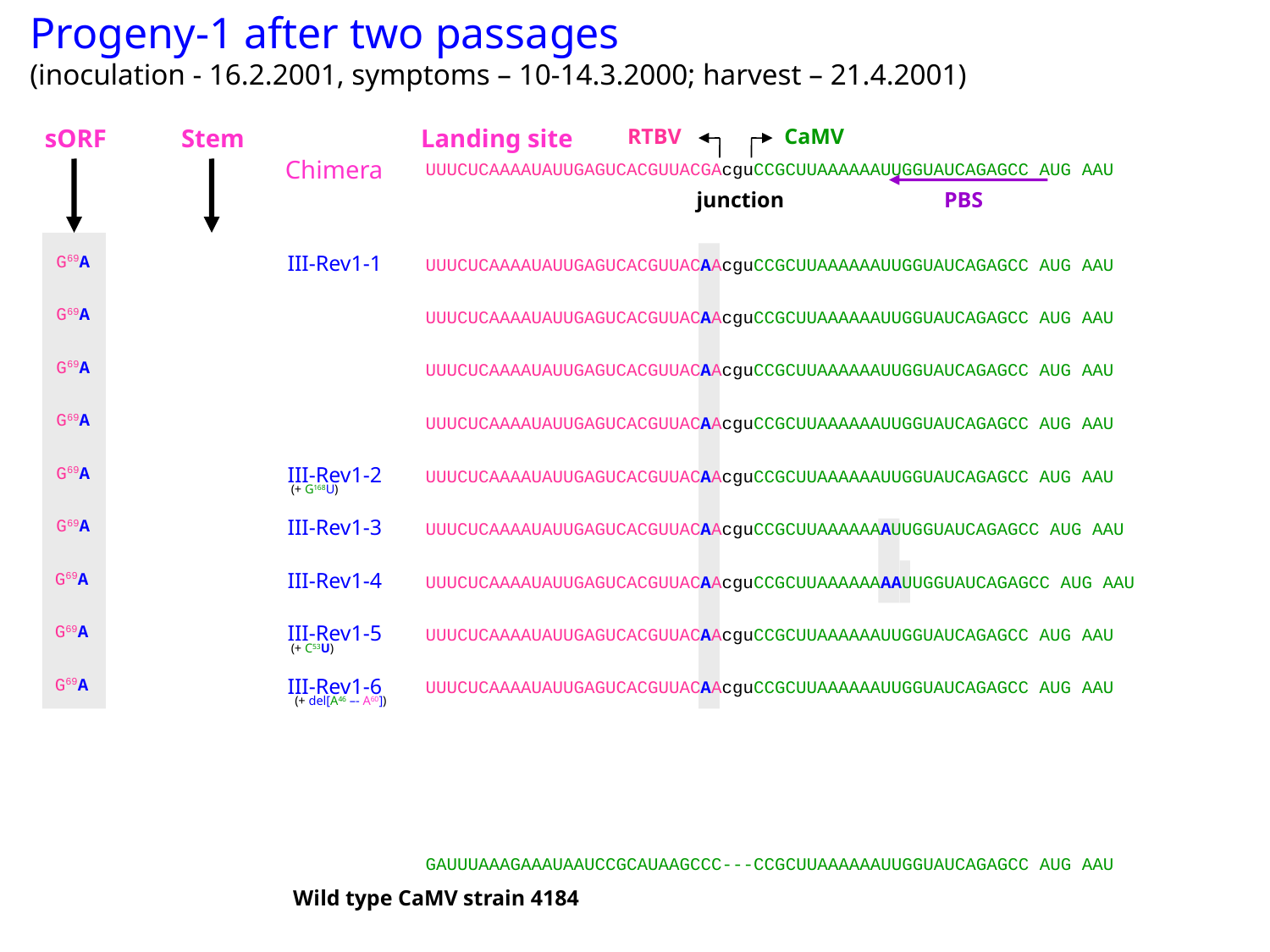

Progeny-1 after two passages
(inoculation - 16.2.2001, symptoms – 10-14.3.2000; harvest – 21.4.2001)
sORF
Stem
Landing site
RTBV
CaMV
Chimera
UUUCUCAAAAUAUUGAGUCACGUUACGAcguCCGCUUAAAAAAUUGGUAUCAGAGCC AUG AAU
PBS
junction
G69A
III-Rev1-1
UUUCUCAAAAUAUUGAGUCACGUUACAAcguCCGCUUAAAAAAUUGGUAUCAGAGCC AUG AAU
G69A
UUUCUCAAAAUAUUGAGUCACGUUACAAcguCCGCUUAAAAAAUUGGUAUCAGAGCC AUG AAU
G69A
UUUCUCAAAAUAUUGAGUCACGUUACAAcguCCGCUUAAAAAAUUGGUAUCAGAGCC AUG AAU
G69A
UUUCUCAAAAUAUUGAGUCACGUUACAAcguCCGCUUAAAAAAUUGGUAUCAGAGCC AUG AAU
G69A
III-Rev1-2
UUUCUCAAAAUAUUGAGUCACGUUACAAcguCCGCUUAAAAAAUUGGUAUCAGAGCC AUG AAU
(+ G168U)
G69A
III-Rev1-3
UUUCUCAAAAUAUUGAGUCACGUUACAAcguCCGCUUAAAAAAAUUGGUAUCAGAGCC AUG AAU
G69A
III-Rev1-4
UUUCUCAAAAUAUUGAGUCACGUUACAAcguCCGCUUAAAAAAAAUUGGUAUCAGAGCC AUG AAU
G69A
III-Rev1-5
UUUCUCAAAAUAUUGAGUCACGUUACAAcguCCGCUUAAAAAAUUGGUAUCAGAGCC AUG AAU
(+ C53U)
G69A
III-Rev1-6
UUUCUCAAAAUAUUGAGUCACGUUACAAcguCCGCUUAAAAAAUUGGUAUCAGAGCC AUG AAU
(+ del[A46 –- A60])
GAUUUAAAGAAAUAAUCCGCAUAAGCCC---CCGCUUAAAAAAUUGGUAUCAGAGCC AUG AAU
Wild type CaMV strain 4184

## Slide 31
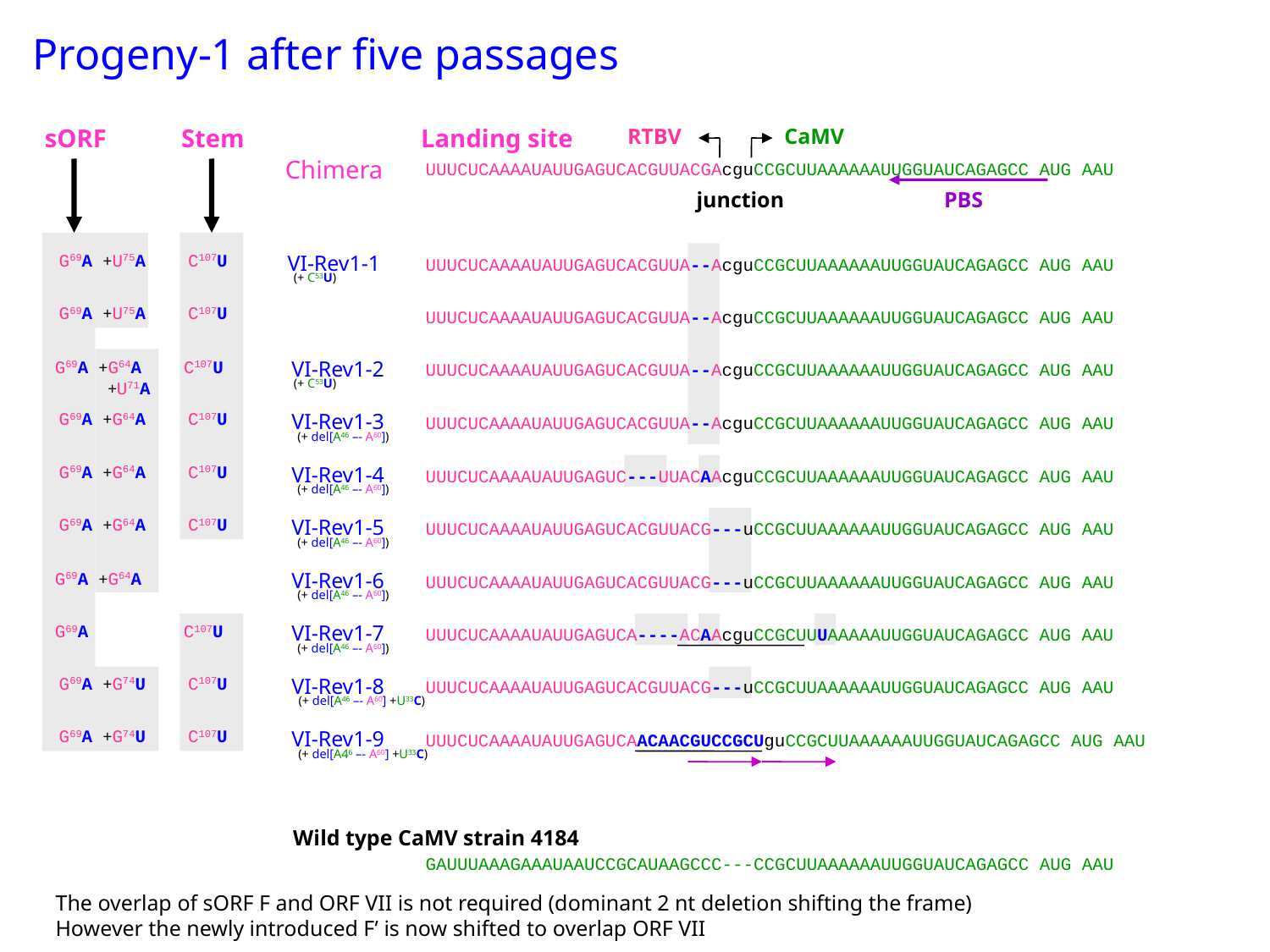

Progeny-1 after five passages
sORF
Stem
Landing site
RTBV
CaMV
Chimera
UUUCUCAAAAUAUUGAGUCACGUUACGAcguCCGCUUAAAAAAUUGGUAUCAGAGCC AUG AAU
PBS
junction
G69A +U75A C107U
VI-Rev1-1
UUUCUCAAAAUAUUGAGUCACGUUA--AcguCCGCUUAAAAAAUUGGUAUCAGAGCC AUG AAU
(+ C53U)
G69A +U75A C107U
UUUCUCAAAAUAUUGAGUCACGUUA--AcguCCGCUUAAAAAAUUGGUAUCAGAGCC AUG AAU
 G69A +G64A C107U
 +U71A
VI-Rev1-2
UUUCUCAAAAUAUUGAGUCACGUUA--AcguCCGCUUAAAAAAUUGGUAUCAGAGCC AUG AAU
(+ C53U)
G69A +G64A C107U
VI-Rev1-3
UUUCUCAAAAUAUUGAGUCACGUUA--AcguCCGCUUAAAAAAUUGGUAUCAGAGCC AUG AAU
(+ del[A46 –- A60])
G69A +G64A C107U
VI-Rev1-4
UUUCUCAAAAUAUUGAGUC---UUACAAcguCCGCUUAAAAAAUUGGUAUCAGAGCC AUG AAU
(+ del[A46 –- A60])
G69A +G64A C107U
VI-Rev1-5
UUUCUCAAAAUAUUGAGUCACGUUACG---uCCGCUUAAAAAAUUGGUAUCAGAGCC AUG AAU
(+ del[A46 –- A60])
G69A +G64A
VI-Rev1-6
UUUCUCAAAAUAUUGAGUCACGUUACG---uCCGCUUAAAAAAUUGGUAUCAGAGCC AUG AAU
(+ del[A46 –- A60])
G69A C107U
VI-Rev1-7
UUUCUCAAAAUAUUGAGUCA----ACAAcguCCGCUUUAAAAAUUGGUAUCAGAGCC AUG AAU
(+ del[A46 –- A60])
G69A +G74U C107U
VI-Rev1-8
UUUCUCAAAAUAUUGAGUCACGUUACG---uCCGCUUAAAAAAUUGGUAUCAGAGCC AUG AAU
(+ del[A46 –- A60] +U33C)
G69A +G74U C107U
VI-Rev1-9
UUUCUCAAAAUAUUGAGUCAACAACGUCCGCUguCCGCUUAAAAAAUUGGUAUCAGAGCC AUG AAU
(+ del[A46 –- A60] +U33C)
Wild type CaMV strain 4184
GAUUUAAAGAAAUAAUCCGCAUAAGCCC---CCGCUUAAAAAAUUGGUAUCAGAGCC AUG AAU
The overlap of sORF F and ORF VII is not required (dominant 2 nt deletion shifting the frame)
However the newly introduced F’ is now shifted to overlap ORF VII

## Slide 32
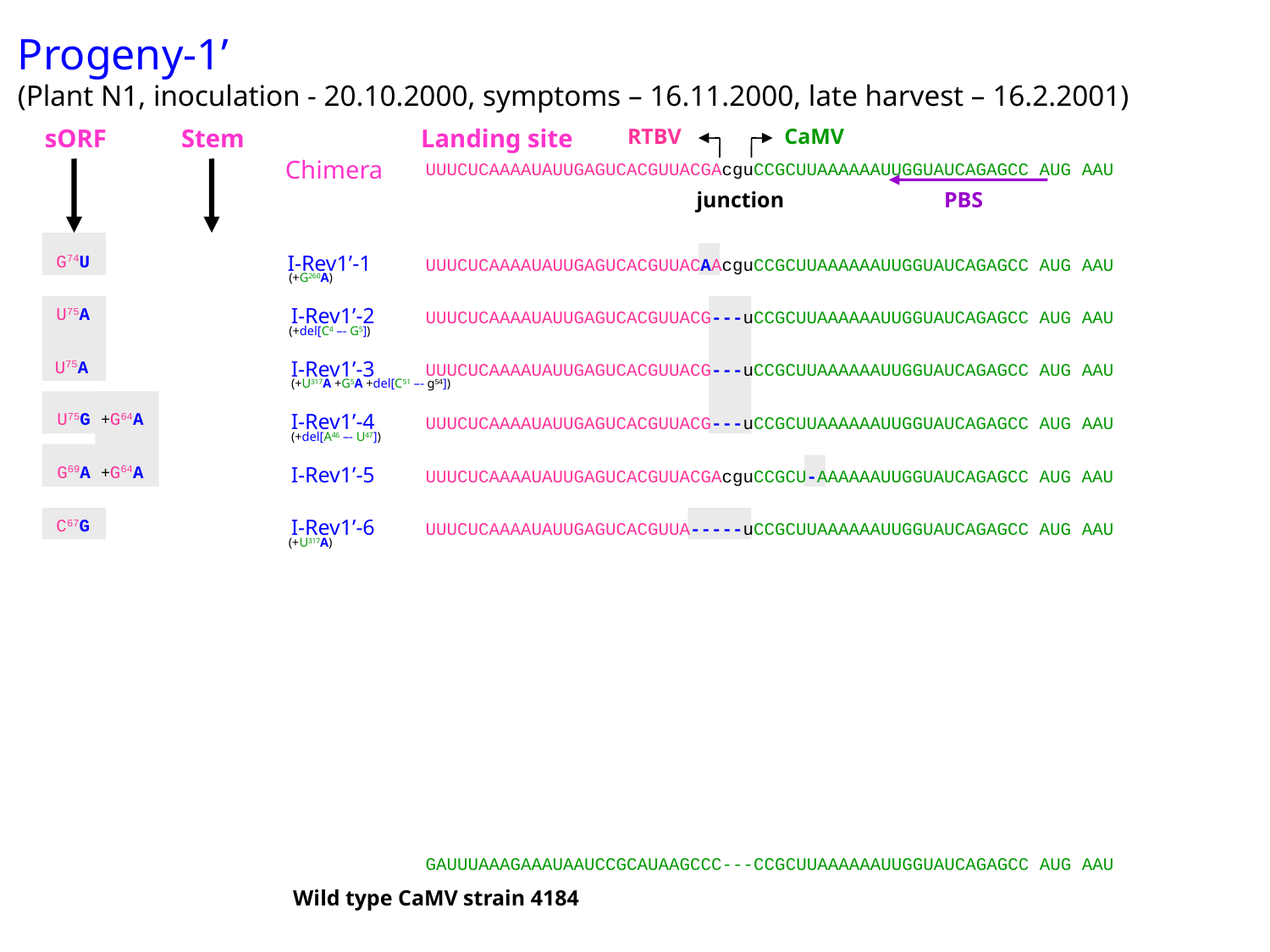

Progeny-1’
(Plant N1, inoculation - 20.10.2000, symptoms – 16.11.2000, late harvest – 16.2.2001)
sORF
Stem
Landing site
RTBV
CaMV
Chimera
UUUCUCAAAAUAUUGAGUCACGUUACGAcguCCGCUUAAAAAAUUGGUAUCAGAGCC AUG AAU
PBS
junction
G74U
I-Rev1’-1
UUUCUCAAAAUAUUGAGUCACGUUACAAcguCCGCUUAAAAAAUUGGUAUCAGAGCC AUG AAU
(+G260A)
U75A
I-Rev1’-2
UUUCUCAAAAUAUUGAGUCACGUUACG---uCCGCUUAAAAAAUUGGUAUCAGAGCC AUG AAU
(+del[C4 –- G5])
 U75A
I-Rev1’-3
UUUCUCAAAAUAUUGAGUCACGUUACG---uCCGCUUAAAAAAUUGGUAUCAGAGCC AUG AAU
(+U317A +G5A +del[C51 –- g54])
U75G +G64A
I-Rev1’-4
UUUCUCAAAAUAUUGAGUCACGUUACG---uCCGCUUAAAAAAUUGGUAUCAGAGCC AUG AAU
(+del[A46 –- U47])
G69A +G64A
I-Rev1’-5
UUUCUCAAAAUAUUGAGUCACGUUACGAcguCCGCU-AAAAAAUUGGUAUCAGAGCC AUG AAU
C67G
I-Rev1’-6
UUUCUCAAAAUAUUGAGUCACGUUA-----uCCGCUUAAAAAAUUGGUAUCAGAGCC AUG AAU
(+U317A)
GAUUUAAAGAAAUAAUCCGCAUAAGCCC---CCGCUUAAAAAAUUGGUAUCAGAGCC AUG AAU
Wild type CaMV strain 4184

## Slide 33
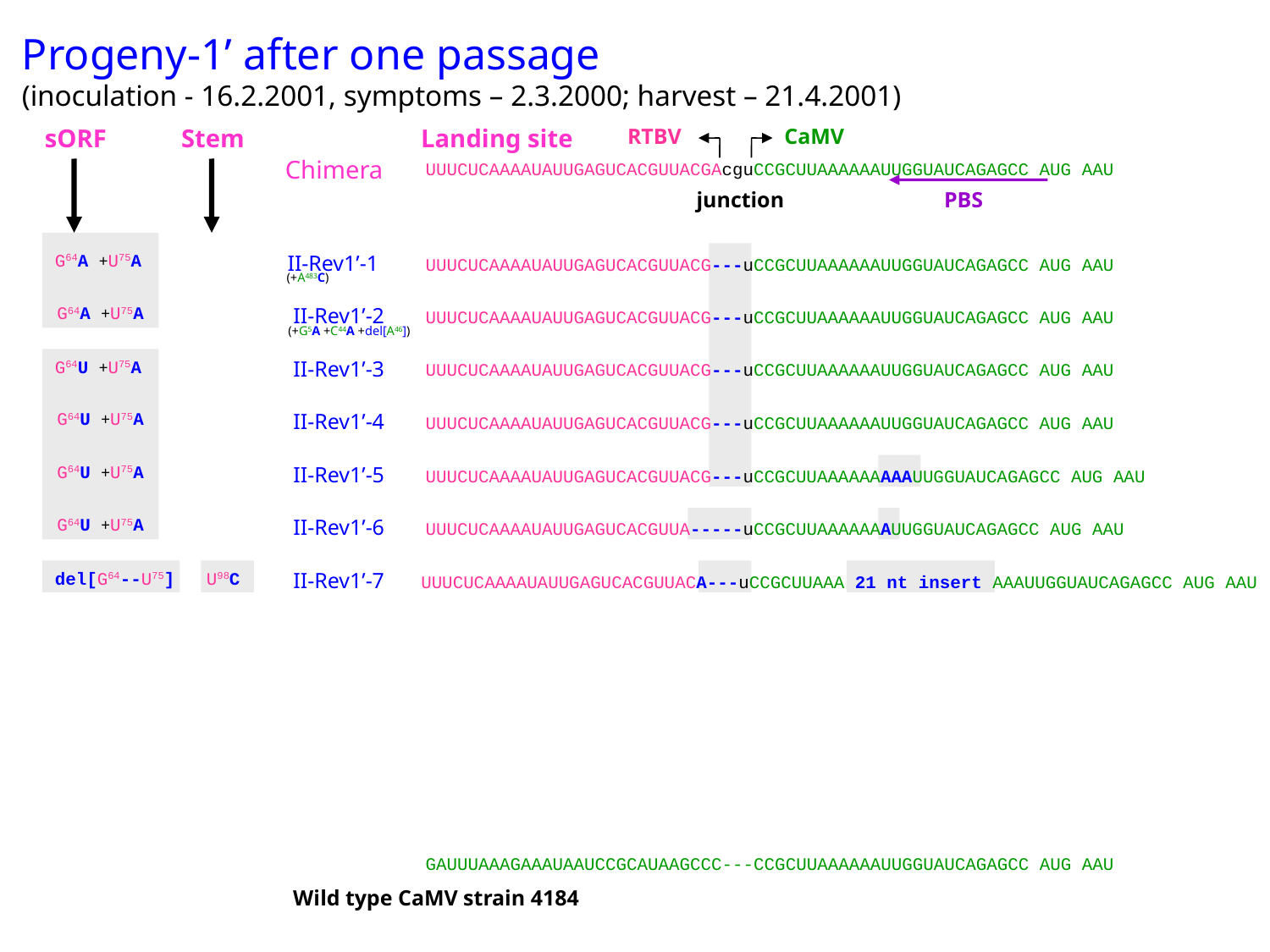

Progeny-1’ after one passage
(inoculation - 16.2.2001, symptoms – 2.3.2000; harvest – 21.4.2001)
sORF
Stem
Landing site
RTBV
CaMV
Chimera
UUUCUCAAAAUAUUGAGUCACGUUACGAcguCCGCUUAAAAAAUUGGUAUCAGAGCC AUG AAU
PBS
junction
G64A +U75A
II-Rev1’-1
UUUCUCAAAAUAUUGAGUCACGUUACG---uCCGCUUAAAAAAUUGGUAUCAGAGCC AUG AAU
(+A483C)
G64A +U75A
II-Rev1’-2
UUUCUCAAAAUAUUGAGUCACGUUACG---uCCGCUUAAAAAAUUGGUAUCAGAGCC AUG AAU
(+G5A +C44A +del[A46])
 G64U +U75A
II-Rev1’-3
UUUCUCAAAAUAUUGAGUCACGUUACG---uCCGCUUAAAAAAUUGGUAUCAGAGCC AUG AAU
G64U +U75A
II-Rev1’-4
UUUCUCAAAAUAUUGAGUCACGUUACG---uCCGCUUAAAAAAUUGGUAUCAGAGCC AUG AAU
G64U +U75A
II-Rev1’-5
UUUCUCAAAAUAUUGAGUCACGUUACG---uCCGCUUAAAAAAAAAUUGGUAUCAGAGCC AUG AAU
G64U +U75A
II-Rev1’-6
UUUCUCAAAAUAUUGAGUCACGUUA-----uCCGCUUAAAAAAAUUGGUAUCAGAGCC AUG AAU
del[G64--U75] U98C
II-Rev1’-7
UUUCUCAAAAUAUUGAGUCACGUUACA---uCCGCUUAAA 21 nt insert AAAUUGGUAUCAGAGCC AUG AAU
GAUUUAAAGAAAUAAUCCGCAUAAGCCC---CCGCUUAAAAAAUUGGUAUCAGAGCC AUG AAU
Wild type CaMV strain 4184

## Slide 34
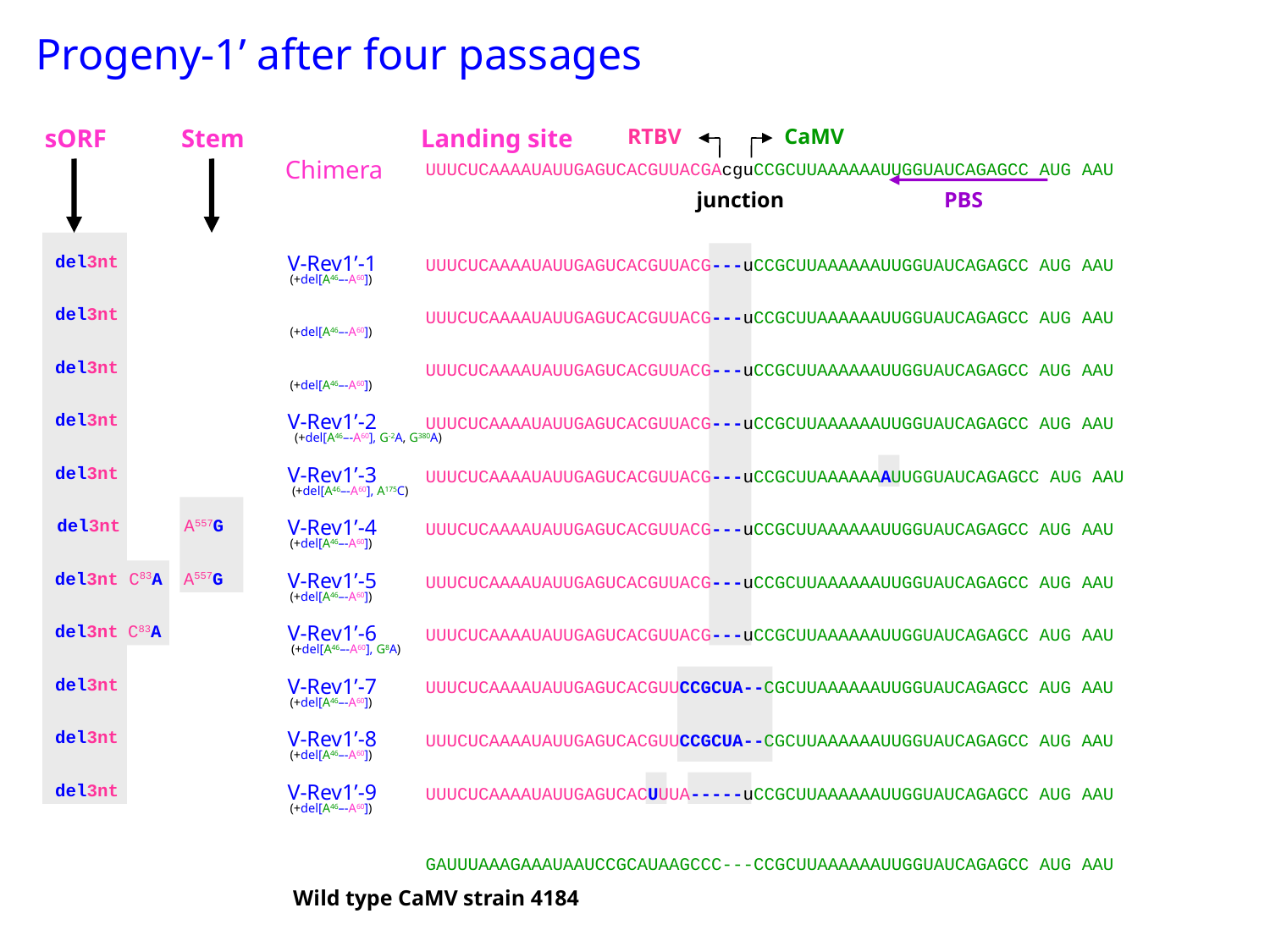

Progeny-1’ after four passages
sORF
Stem
Landing site
RTBV
CaMV
Chimera
UUUCUCAAAAUAUUGAGUCACGUUACGAcguCCGCUUAAAAAAUUGGUAUCAGAGCC AUG AAU
PBS
junction
del3nt
V-Rev1’-1
UUUCUCAAAAUAUUGAGUCACGUUACG---uCCGCUUAAAAAAUUGGUAUCAGAGCC AUG AAU
(+del[A46–-A60])
del3nt
UUUCUCAAAAUAUUGAGUCACGUUACG---uCCGCUUAAAAAAUUGGUAUCAGAGCC AUG AAU
(+del[A46–-A60])
del3nt
UUUCUCAAAAUAUUGAGUCACGUUACG---uCCGCUUAAAAAAUUGGUAUCAGAGCC AUG AAU
(+del[A46–-A60])
del3nt
V-Rev1’-2
UUUCUCAAAAUAUUGAGUCACGUUACG---uCCGCUUAAAAAAUUGGUAUCAGAGCC AUG AAU
(+del[A46–-A60], G-2A, G380A)
del3nt
V-Rev1’-3
UUUCUCAAAAUAUUGAGUCACGUUACG---uCCGCUUAAAAAAAUUGGUAUCAGAGCC AUG AAU
(+del[A46–-A60], A175C)
del3nt A557G
V-Rev1’-4
UUUCUCAAAAUAUUGAGUCACGUUACG---uCCGCUUAAAAAAUUGGUAUCAGAGCC AUG AAU
(+del[A46–-A60])
del3nt C83A A557G
V-Rev1’-5
UUUCUCAAAAUAUUGAGUCACGUUACG---uCCGCUUAAAAAAUUGGUAUCAGAGCC AUG AAU
(+del[A46–-A60])
del3nt C83A
V-Rev1’-6
UUUCUCAAAAUAUUGAGUCACGUUACG---uCCGCUUAAAAAAUUGGUAUCAGAGCC AUG AAU
(+del[A46–-A60], G8A)
del3nt
V-Rev1’-7
UUUCUCAAAAUAUUGAGUCACGUUCCGCUA--CGCUUAAAAAAUUGGUAUCAGAGCC AUG AAU
(+del[A46–-A60])
del3nt
V-Rev1’-8
UUUCUCAAAAUAUUGAGUCACGUUCCGCUA--CGCUUAAAAAAUUGGUAUCAGAGCC AUG AAU
(+del[A46–-A60])
del3nt
V-Rev1’-9
UUUCUCAAAAUAUUGAGUCACUUUA-----uCCGCUUAAAAAAUUGGUAUCAGAGCC AUG AAU
(+del[A46–-A60])
GAUUUAAAGAAAUAAUCCGCAUAAGCCC---CCGCUUAAAAAAUUGGUAUCAGAGCC AUG AAU
Wild type CaMV strain 4184

## Slide 35
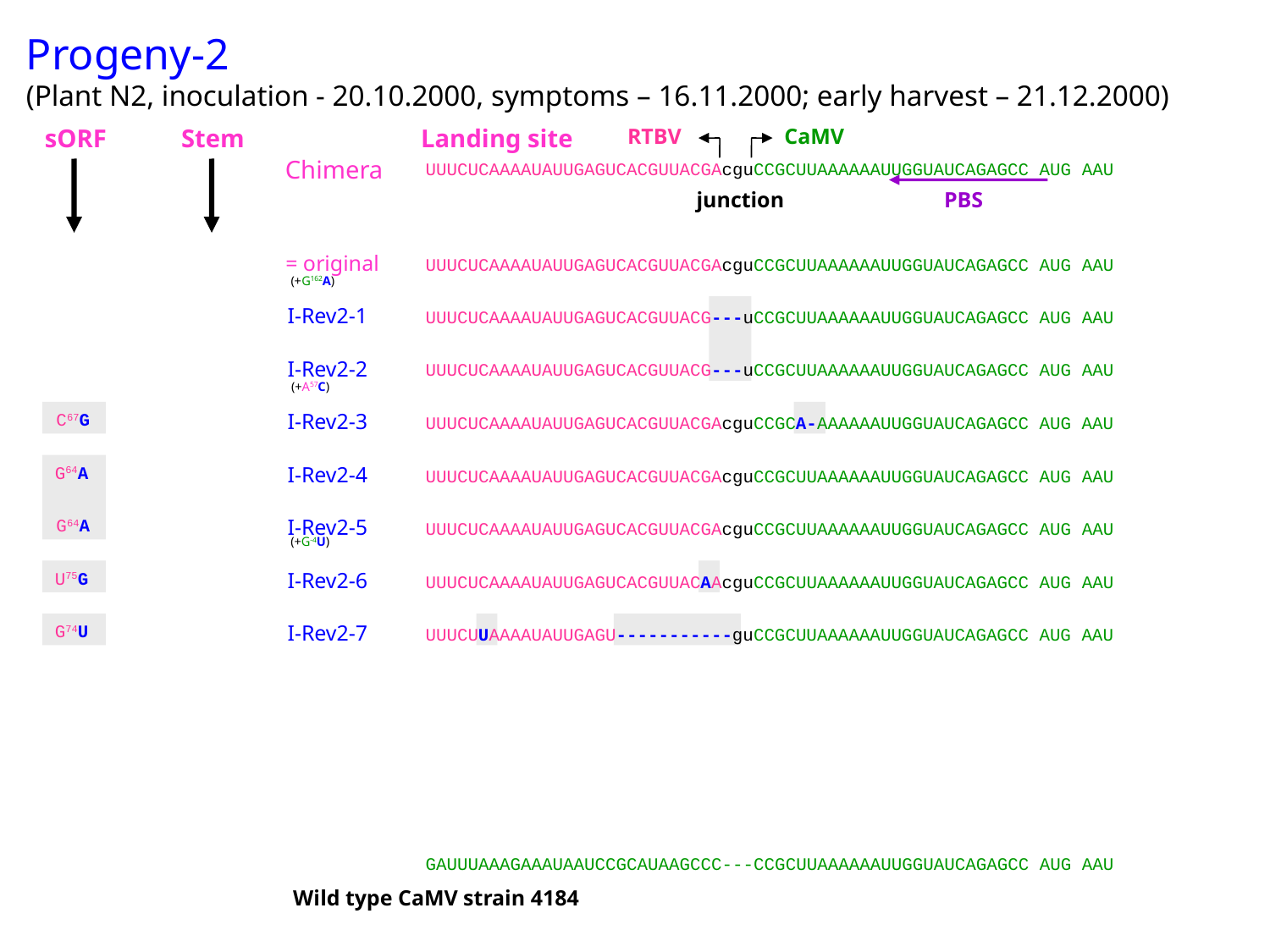

Progeny-2
(Plant N2, inoculation - 20.10.2000, symptoms – 16.11.2000; early harvest – 21.12.2000)
sORF
Stem
Landing site
RTBV
CaMV
Chimera
UUUCUCAAAAUAUUGAGUCACGUUACGAcguCCGCUUAAAAAAUUGGUAUCAGAGCC AUG AAU
PBS
junction
= original
UUUCUCAAAAUAUUGAGUCACGUUACGAcguCCGCUUAAAAAAUUGGUAUCAGAGCC AUG AAU
(+G162A)
I-Rev2-1
UUUCUCAAAAUAUUGAGUCACGUUACG---uCCGCUUAAAAAAUUGGUAUCAGAGCC AUG AAU
I-Rev2-2
UUUCUCAAAAUAUUGAGUCACGUUACG---uCCGCUUAAAAAAUUGGUAUCAGAGCC AUG AAU
(+A57C)
C67G
I-Rev2-3
UUUCUCAAAAUAUUGAGUCACGUUACGAcguCCGCA-AAAAAAUUGGUAUCAGAGCC AUG AAU
G64A
I-Rev2-4
UUUCUCAAAAUAUUGAGUCACGUUACGAcguCCGCUUAAAAAAUUGGUAUCAGAGCC AUG AAU
G64A
I-Rev2-5
UUUCUCAAAAUAUUGAGUCACGUUACGAcguCCGCUUAAAAAAUUGGUAUCAGAGCC AUG AAU
(+G-4U)
U75G
I-Rev2-6
UUUCUCAAAAUAUUGAGUCACGUUACAAcguCCGCUUAAAAAAUUGGUAUCAGAGCC AUG AAU
G74U
I-Rev2-7
UUUCUUAAAAUAUUGAGU-----------guCCGCUUAAAAAAUUGGUAUCAGAGCC AUG AAU
GAUUUAAAGAAAUAAUCCGCAUAAGCCC---CCGCUUAAAAAAUUGGUAUCAGAGCC AUG AAU
Wild type CaMV strain 4184

## Slide 36
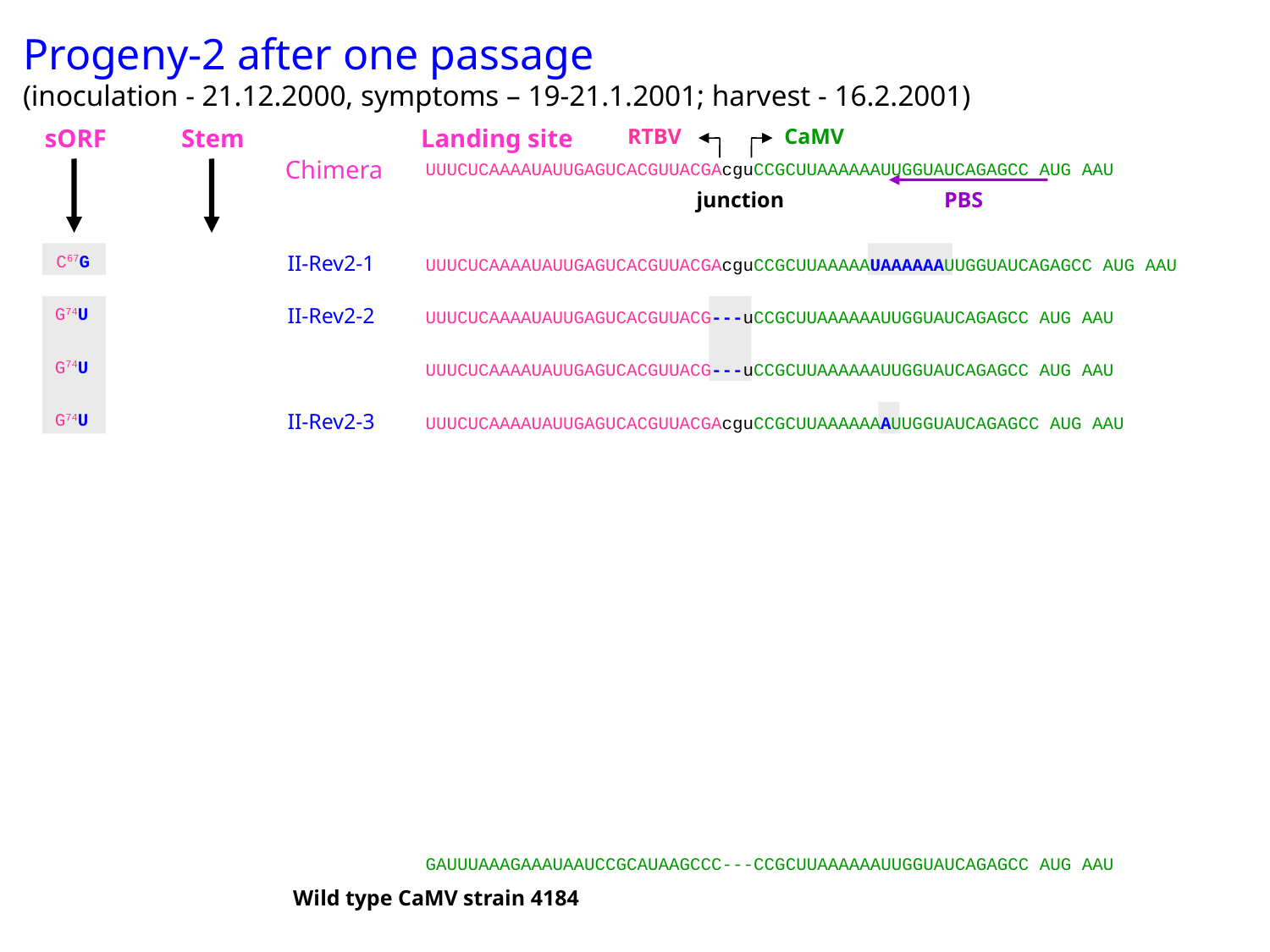

Progeny-2 after one passage
(inoculation - 21.12.2000, symptoms – 19-21.1.2001; harvest - 16.2.2001)
sORF
Stem
Landing site
RTBV
CaMV
Chimera
UUUCUCAAAAUAUUGAGUCACGUUACGAcguCCGCUUAAAAAAUUGGUAUCAGAGCC AUG AAU
PBS
junction
C67G
II-Rev2-1
UUUCUCAAAAUAUUGAGUCACGUUACGAcguCCGCUUAAAAAUAAAAAAUUGGUAUCAGAGCC AUG AAU
G74U
II-Rev2-2
UUUCUCAAAAUAUUGAGUCACGUUACG---uCCGCUUAAAAAAUUGGUAUCAGAGCC AUG AAU
G74U
UUUCUCAAAAUAUUGAGUCACGUUACG---uCCGCUUAAAAAAUUGGUAUCAGAGCC AUG AAU
G74U
II-Rev2-3
UUUCUCAAAAUAUUGAGUCACGUUACGAcguCCGCUUAAAAAAAUUGGUAUCAGAGCC AUG AAU
GAUUUAAAGAAAUAAUCCGCAUAAGCCC---CCGCUUAAAAAAUUGGUAUCAGAGCC AUG AAU
Wild type CaMV strain 4184

## Slide 37
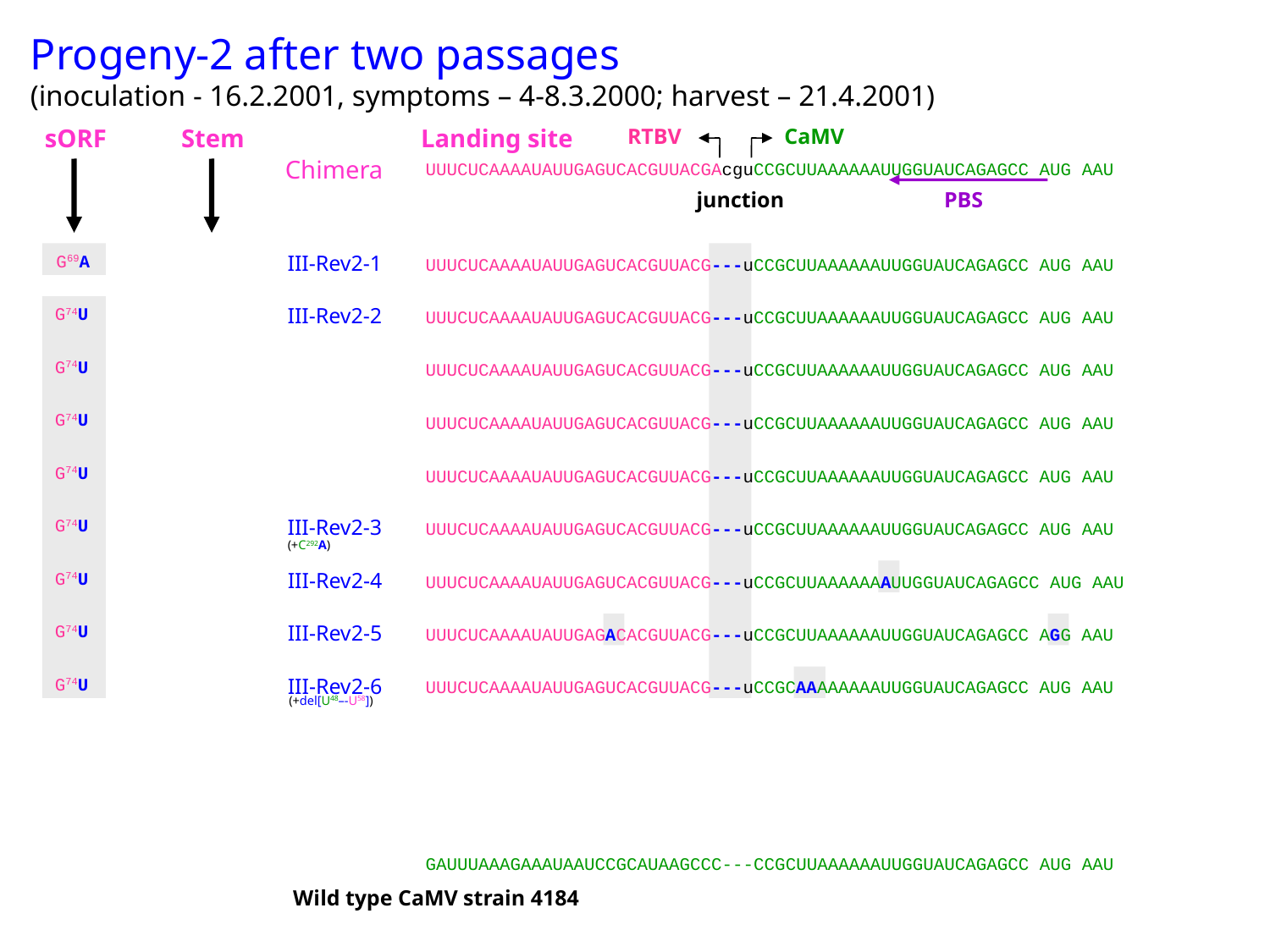

Progeny-2 after two passages
(inoculation - 16.2.2001, symptoms – 4-8.3.2000; harvest – 21.4.2001)
sORF
Stem
Landing site
RTBV
CaMV
Chimera
UUUCUCAAAAUAUUGAGUCACGUUACGAcguCCGCUUAAAAAAUUGGUAUCAGAGCC AUG AAU
PBS
junction
G69A
III-Rev2-1
UUUCUCAAAAUAUUGAGUCACGUUACG---uCCGCUUAAAAAAUUGGUAUCAGAGCC AUG AAU
G74U
III-Rev2-2
UUUCUCAAAAUAUUGAGUCACGUUACG---uCCGCUUAAAAAAUUGGUAUCAGAGCC AUG AAU
G74U
UUUCUCAAAAUAUUGAGUCACGUUACG---uCCGCUUAAAAAAUUGGUAUCAGAGCC AUG AAU
G74U
UUUCUCAAAAUAUUGAGUCACGUUACG---uCCGCUUAAAAAAUUGGUAUCAGAGCC AUG AAU
G74U
UUUCUCAAAAUAUUGAGUCACGUUACG---uCCGCUUAAAAAAUUGGUAUCAGAGCC AUG AAU
G74U
III-Rev2-3
UUUCUCAAAAUAUUGAGUCACGUUACG---uCCGCUUAAAAAAUUGGUAUCAGAGCC AUG AAU
(+C292A)
G74U
III-Rev2-4
UUUCUCAAAAUAUUGAGUCACGUUACG---uCCGCUUAAAAAAAUUGGUAUCAGAGCC AUG AAU
G74U
III-Rev2-5
UUUCUCAAAAUAUUGAGACACGUUACG---uCCGCUUAAAAAAUUGGUAUCAGAGCC AGG AAU
G74U
III-Rev2-6
UUUCUCAAAAUAUUGAGUCACGUUACG---uCCGCAAAAAAAAUUGGUAUCAGAGCC AUG AAU
(+del[U48–-U58])
GAUUUAAAGAAAUAAUCCGCAUAAGCCC---CCGCUUAAAAAAUUGGUAUCAGAGCC AUG AAU
Wild type CaMV strain 4184

## Slide 38
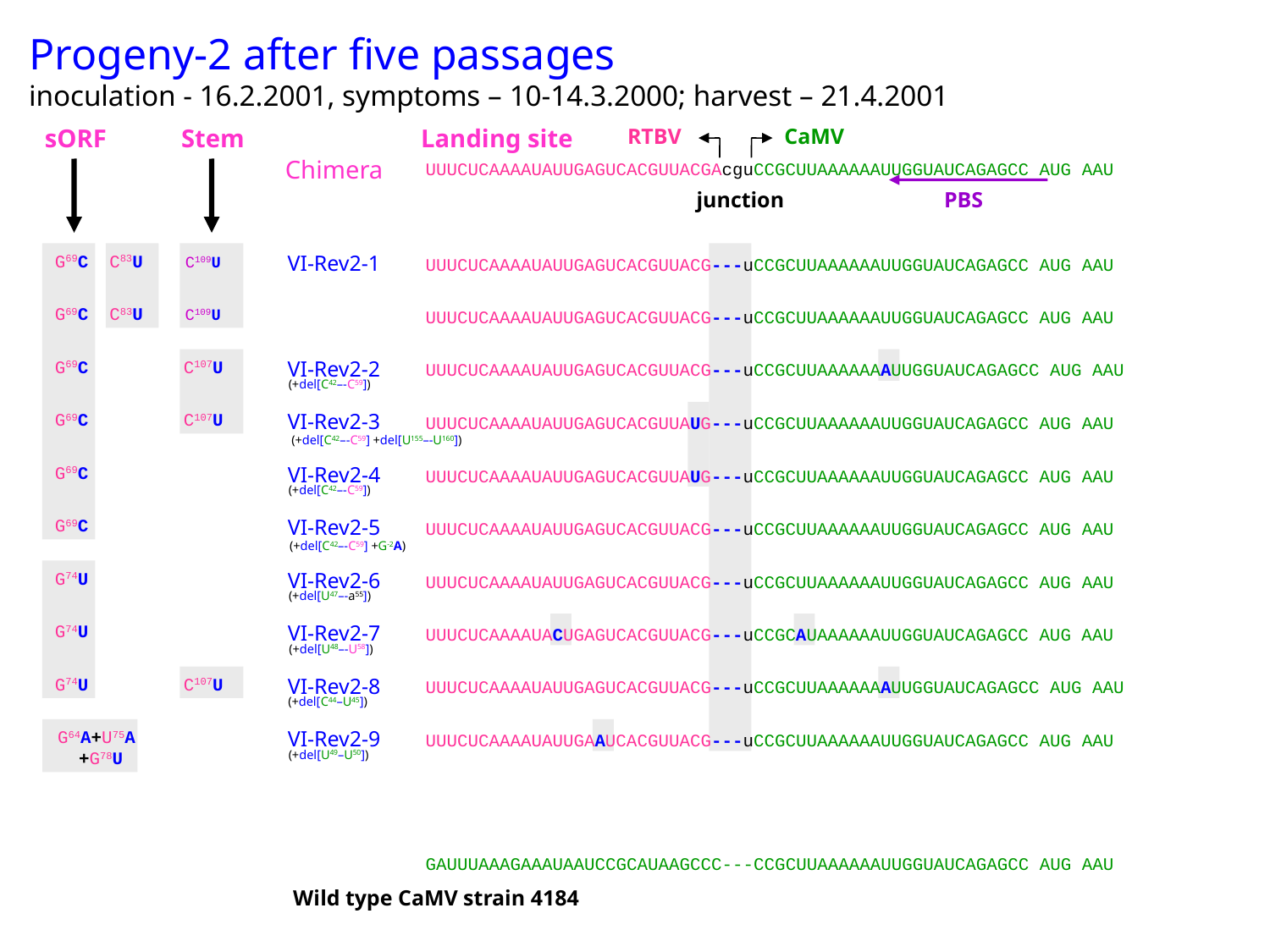

Progeny-2 after five passages
inoculation - 16.2.2001, symptoms – 10-14.3.2000; harvest – 21.4.2001
sORF
Stem
Landing site
RTBV
CaMV
Chimera
UUUCUCAAAAUAUUGAGUCACGUUACGAcguCCGCUUAAAAAAUUGGUAUCAGAGCC AUG AAU
PBS
junction
G69C C83U C109U
VI-Rev2-1
UUUCUCAAAAUAUUGAGUCACGUUACG---uCCGCUUAAAAAAUUGGUAUCAGAGCC AUG AAU
G69C C83U C109U
UUUCUCAAAAUAUUGAGUCACGUUACG---uCCGCUUAAAAAAUUGGUAUCAGAGCC AUG AAU
G69C C107U
VI-Rev2-2
UUUCUCAAAAUAUUGAGUCACGUUACG---uCCGCUUAAAAAAAUUGGUAUCAGAGCC AUG AAU
(+del[C42–-C59])
G69C C107U
VI-Rev2-3
UUUCUCAAAAUAUUGAGUCACGUUAUG---uCCGCUUAAAAAAUUGGUAUCAGAGCC AUG AAU
(+del[C42–-C59] +del[U155–-U160])
G69C
VI-Rev2-4
UUUCUCAAAAUAUUGAGUCACGUUAUG---uCCGCUUAAAAAAUUGGUAUCAGAGCC AUG AAU
(+del[C42–-C59])
G69C
VI-Rev2-5
UUUCUCAAAAUAUUGAGUCACGUUACG---uCCGCUUAAAAAAUUGGUAUCAGAGCC AUG AAU
(+del[C42–-C59] +G-2A)
G74U
VI-Rev2-6
UUUCUCAAAAUAUUGAGUCACGUUACG---uCCGCUUAAAAAAUUGGUAUCAGAGCC AUG AAU
(+del[U47–-a55])
G74U
VI-Rev2-7
UUUCUCAAAAUACUGAGUCACGUUACG---uCCGCAUAAAAAAUUGGUAUCAGAGCC AUG AAU
(+del[U48–-U58])
G74U C107U
VI-Rev2-8
UUUCUCAAAAUAUUGAGUCACGUUACG---uCCGCUUAAAAAAAUUGGUAUCAGAGCC AUG AAU
(+del[C44–U45])
G64A+U75A
 +G78U
VI-Rev2-9
UUUCUCAAAAUAUUGAAUCACGUUACG---uCCGCUUAAAAAAUUGGUAUCAGAGCC AUG AAU
(+del[U49–U50])
GAUUUAAAGAAAUAAUCCGCAUAAGCCC---CCGCUUAAAAAAUUGGUAUCAGAGCC AUG AAU
Wild type CaMV strain 4184

## Slide 39
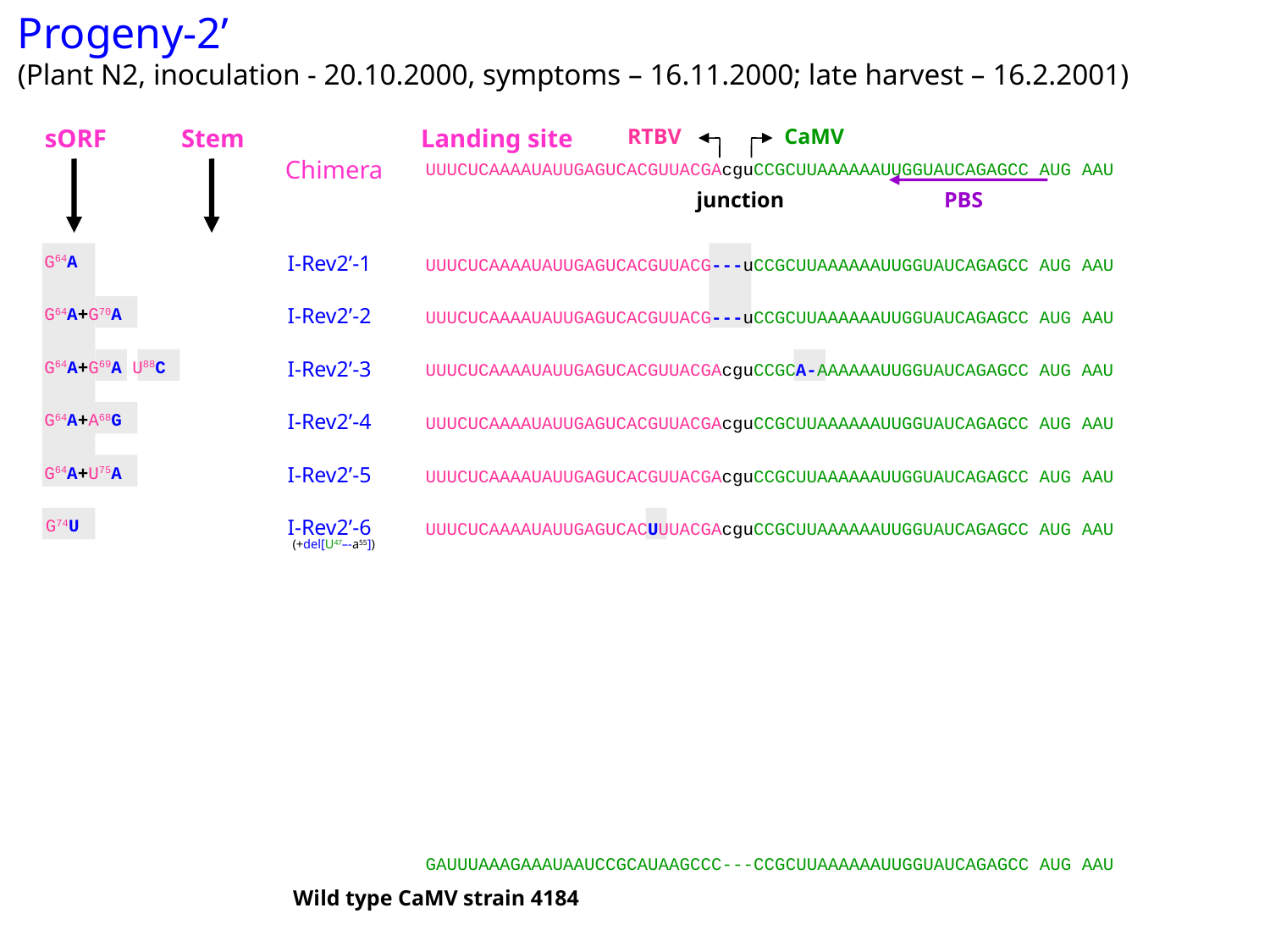

Progeny-2’
(Plant N2, inoculation - 20.10.2000, symptoms – 16.11.2000; late harvest – 16.2.2001)
sORF
Stem
Landing site
RTBV
CaMV
Chimera
UUUCUCAAAAUAUUGAGUCACGUUACGAcguCCGCUUAAAAAAUUGGUAUCAGAGCC AUG AAU
PBS
junction
G64A
I-Rev2’-1
UUUCUCAAAAUAUUGAGUCACGUUACG---uCCGCUUAAAAAAUUGGUAUCAGAGCC AUG AAU
G64A+G70A
I-Rev2’-2
UUUCUCAAAAUAUUGAGUCACGUUACG---uCCGCUUAAAAAAUUGGUAUCAGAGCC AUG AAU
G64A+G69A U88C
I-Rev2’-3
UUUCUCAAAAUAUUGAGUCACGUUACGAcguCCGCA-AAAAAAUUGGUAUCAGAGCC AUG AAU
G64A+A68G
I-Rev2’-4
UUUCUCAAAAUAUUGAGUCACGUUACGAcguCCGCUUAAAAAAUUGGUAUCAGAGCC AUG AAU
G64A+U75A
I-Rev2’-5
UUUCUCAAAAUAUUGAGUCACGUUACGAcguCCGCUUAAAAAAUUGGUAUCAGAGCC AUG AAU
G74U
I-Rev2’-6
UUUCUCAAAAUAUUGAGUCACUUUACGAcguCCGCUUAAAAAAUUGGUAUCAGAGCC AUG AAU
(+del[U47–-a55])
GAUUUAAAGAAAUAAUCCGCAUAAGCCC---CCGCUUAAAAAAUUGGUAUCAGAGCC AUG AAU
Wild type CaMV strain 4184

## Slide 40
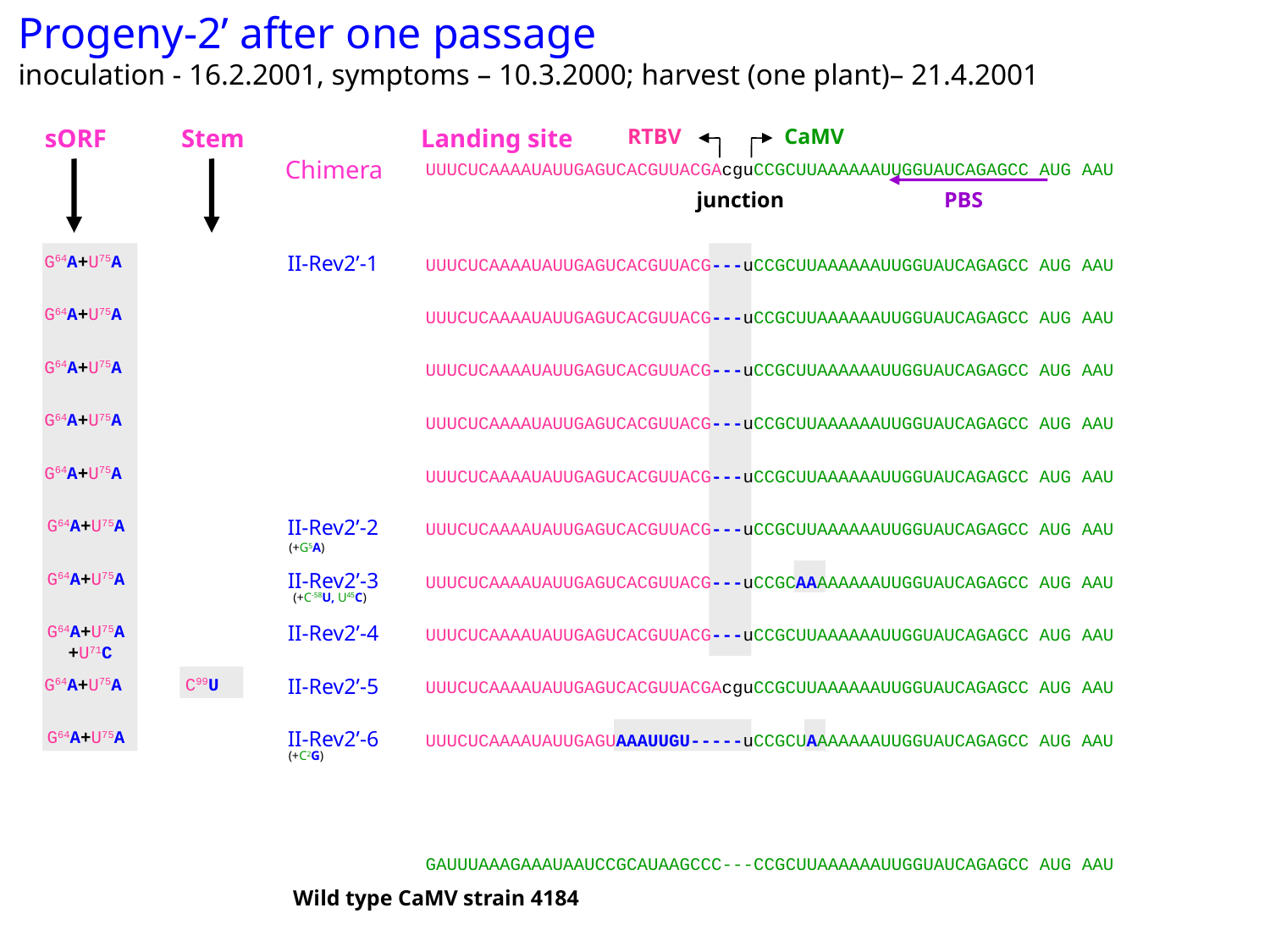

Progeny-2’ after one passage
inoculation - 16.2.2001, symptoms – 10.3.2000; harvest (one plant)– 21.4.2001
sORF
Stem
Landing site
RTBV
CaMV
Chimera
UUUCUCAAAAUAUUGAGUCACGUUACGAcguCCGCUUAAAAAAUUGGUAUCAGAGCC AUG AAU
PBS
junction
G64A+U75A
II-Rev2’-1
UUUCUCAAAAUAUUGAGUCACGUUACG---uCCGCUUAAAAAAUUGGUAUCAGAGCC AUG AAU
G64A+U75A
UUUCUCAAAAUAUUGAGUCACGUUACG---uCCGCUUAAAAAAUUGGUAUCAGAGCC AUG AAU
G64A+U75A
UUUCUCAAAAUAUUGAGUCACGUUACG---uCCGCUUAAAAAAUUGGUAUCAGAGCC AUG AAU
G64A+U75A
UUUCUCAAAAUAUUGAGUCACGUUACG---uCCGCUUAAAAAAUUGGUAUCAGAGCC AUG AAU
G64A+U75A
UUUCUCAAAAUAUUGAGUCACGUUACG---uCCGCUUAAAAAAUUGGUAUCAGAGCC AUG AAU
G64A+U75A
II-Rev2’-2
UUUCUCAAAAUAUUGAGUCACGUUACG---uCCGCUUAAAAAAUUGGUAUCAGAGCC AUG AAU
(+G5A)
G64A+U75A
II-Rev2’-3
UUUCUCAAAAUAUUGAGUCACGUUACG---uCCGCAAAAAAAAUUGGUAUCAGAGCC AUG AAU
(+C-58U, U45C)
G64A+U75A
 +U71C
II-Rev2’-4
UUUCUCAAAAUAUUGAGUCACGUUACG---uCCGCUUAAAAAAUUGGUAUCAGAGCC AUG AAU
G64A+U75A C99U
II-Rev2’-5
UUUCUCAAAAUAUUGAGUCACGUUACGAcguCCGCUUAAAAAAUUGGUAUCAGAGCC AUG AAU
G64A+U75A
II-Rev2’-6
UUUCUCAAAAUAUUGAGUAAAUUGU-----uCCGCUAAAAAAAUUGGUAUCAGAGCC AUG AAU
(+C2G)
GAUUUAAAGAAAUAAUCCGCAUAAGCCC---CCGCUUAAAAAAUUGGUAUCAGAGCC AUG AAU
Wild type CaMV strain 4184

## Slide 41
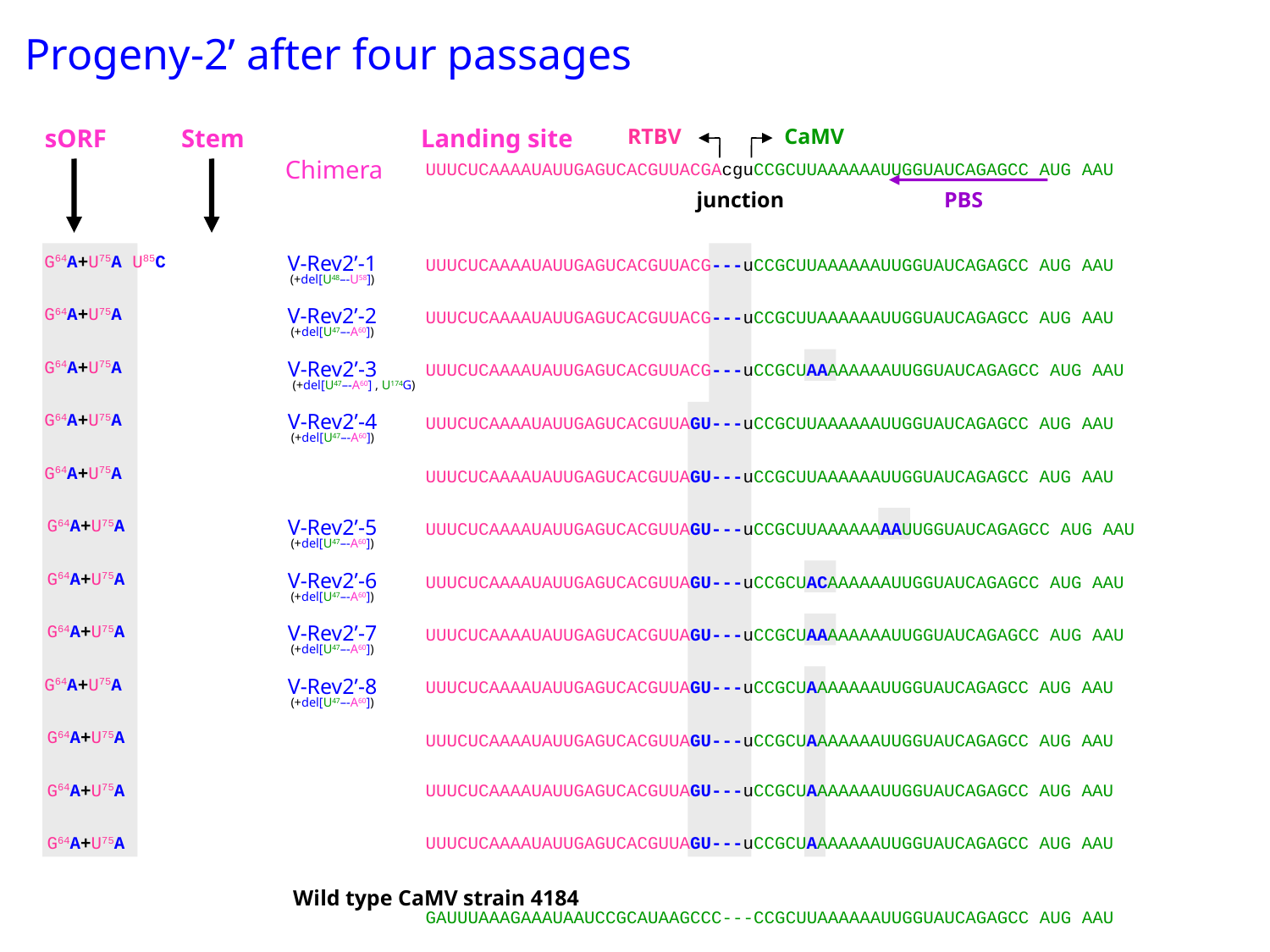

Progeny-2’ after four passages
sORF
Stem
Landing site
RTBV
CaMV
Chimera
UUUCUCAAAAUAUUGAGUCACGUUACGAcguCCGCUUAAAAAAUUGGUAUCAGAGCC AUG AAU
PBS
junction
G64A+U75A U85C
V-Rev2’-1
UUUCUCAAAAUAUUGAGUCACGUUACG---uCCGCUUAAAAAAUUGGUAUCAGAGCC AUG AAU
(+del[U48–-U58])
G64A+U75A
V-Rev2’-2
UUUCUCAAAAUAUUGAGUCACGUUACG---uCCGCUUAAAAAAUUGGUAUCAGAGCC AUG AAU
(+del[U47–-A60])
G64A+U75A
V-Rev2’-3
UUUCUCAAAAUAUUGAGUCACGUUACG---uCCGCUAAAAAAAAUUGGUAUCAGAGCC AUG AAU
(+del[U47–-A60] , U174G)
G64A+U75A
V-Rev2’-4
UUUCUCAAAAUAUUGAGUCACGUUAGU---uCCGCUUAAAAAAUUGGUAUCAGAGCC AUG AAU
(+del[U47–-A60])
G64A+U75A
UUUCUCAAAAUAUUGAGUCACGUUAGU---uCCGCUUAAAAAAUUGGUAUCAGAGCC AUG AAU
G64A+U75A
V-Rev2’-5
UUUCUCAAAAUAUUGAGUCACGUUAGU---uCCGCUUAAAAAAAAUUGGUAUCAGAGCC AUG AAU
(+del[U47–-A60])
G64A+U75A
V-Rev2’-6
UUUCUCAAAAUAUUGAGUCACGUUAGU---uCCGCUACAAAAAAUUGGUAUCAGAGCC AUG AAU
(+del[U47–-A60])
G64A+U75A
V-Rev2’-7
UUUCUCAAAAUAUUGAGUCACGUUAGU---uCCGCUAAAAAAAAUUGGUAUCAGAGCC AUG AAU
(+del[U47–-A60])
G64A+U75A
V-Rev2’-8
UUUCUCAAAAUAUUGAGUCACGUUAGU---uCCGCUAAAAAAAUUGGUAUCAGAGCC AUG AAU
(+del[U47–-A60])
G64A+U75A
UUUCUCAAAAUAUUGAGUCACGUUAGU---uCCGCUAAAAAAAUUGGUAUCAGAGCC AUG AAU
G64A+U75A
UUUCUCAAAAUAUUGAGUCACGUUAGU---uCCGCUAAAAAAAUUGGUAUCAGAGCC AUG AAU
G64A+U75A
UUUCUCAAAAUAUUGAGUCACGUUAGU---uCCGCUAAAAAAAUUGGUAUCAGAGCC AUG AAU
Wild type CaMV strain 4184
GAUUUAAAGAAAUAAUCCGCAUAAGCCC---CCGCUUAAAAAAUUGGUAUCAGAGCC AUG AAU

## Slide 42
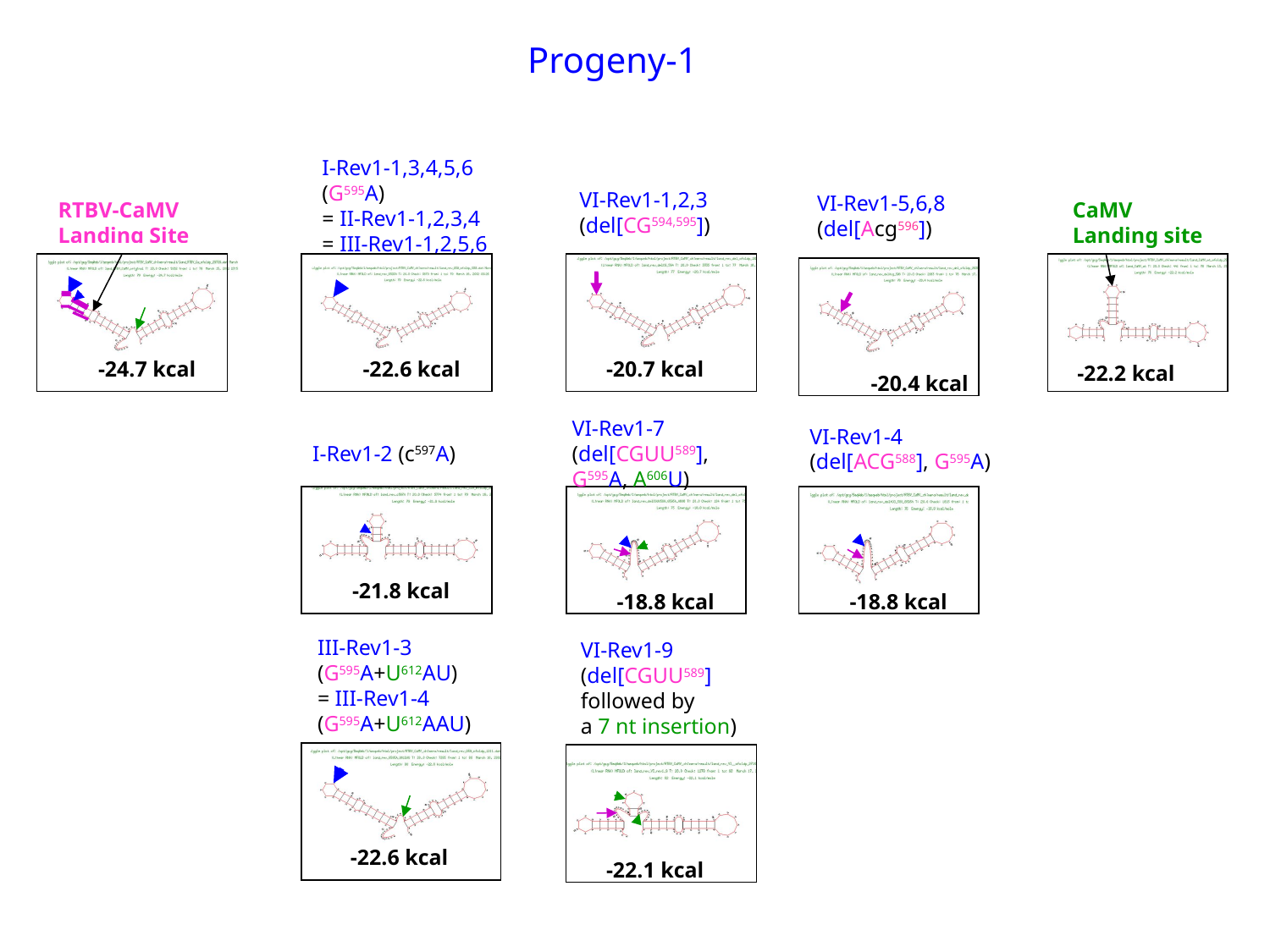

Progeny-1
I-Rev1-1,3,4,5,6
(G595A)
= II-Rev1-1,2,3,4
= III-Rev1-1,2,5,6
VI-Rev1-1,2,3
(del[CG594,595])
VI-Rev1-5,6,8
(del[Acg596])
RTBV-CaMV
Landing Site
CaMV
Landing site
-24.7 kcal
-22.6 kcal
-20.7 kcal
-22.2 kcal
-20.4 kcal
VI-Rev1-7
(del[CGUU589],
G595A, A606U)
VI-Rev1-4
(del[ACG588], G595A)
I-Rev1-2 (c597A)
-21.8 kcal
-18.8 kcal
-18.8 kcal
III-Rev1-3
(G595A+U612AU)
= III-Rev1-4
(G595A+U612AAU)
VI-Rev1-9
(del[CGUU589]
followed by
a 7 nt insertion)
-22.6 kcal
-22.1 kcal

## Slide 43
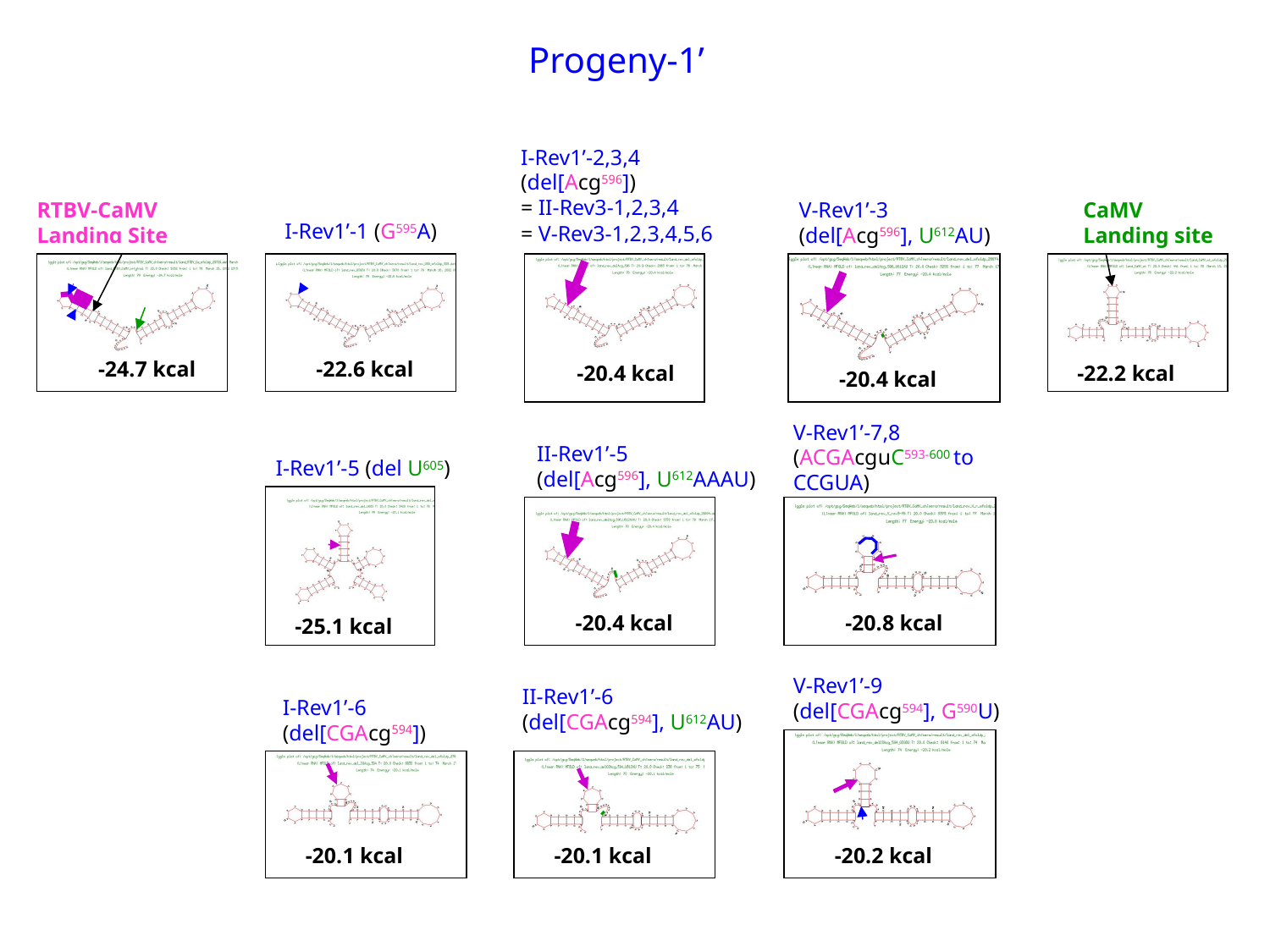

Progeny-1’
I-Rev1’-2,3,4
(del[Acg596])
= II-Rev3-1,2,3,4
= V-Rev3-1,2,3,4,5,6
RTBV-CaMV
Landing Site
V-Rev1’-3
(del[Acg596], U612AU)
CaMV
Landing site
I-Rev1’-1 (G595A)
-24.7 kcal
-22.6 kcal
-20.4 kcal
-22.2 kcal
-20.4 kcal
V-Rev1’-7,8
(ACGAcguC593-600 to
CCGUA)
II-Rev1’-5
(del[Acg596], U612AAAU)
I-Rev1’-5 (del U605)
-20.4 kcal
-20.8 kcal
-25.1 kcal
V-Rev1’-9
(del[CGAcg594], G590U)
II-Rev1’-6
(del[CGAcg594], U612AU)
I-Rev1’-6
(del[CGAcg594])
-20.1 kcal
-20.1 kcal
-20.2 kcal

## Slide 44
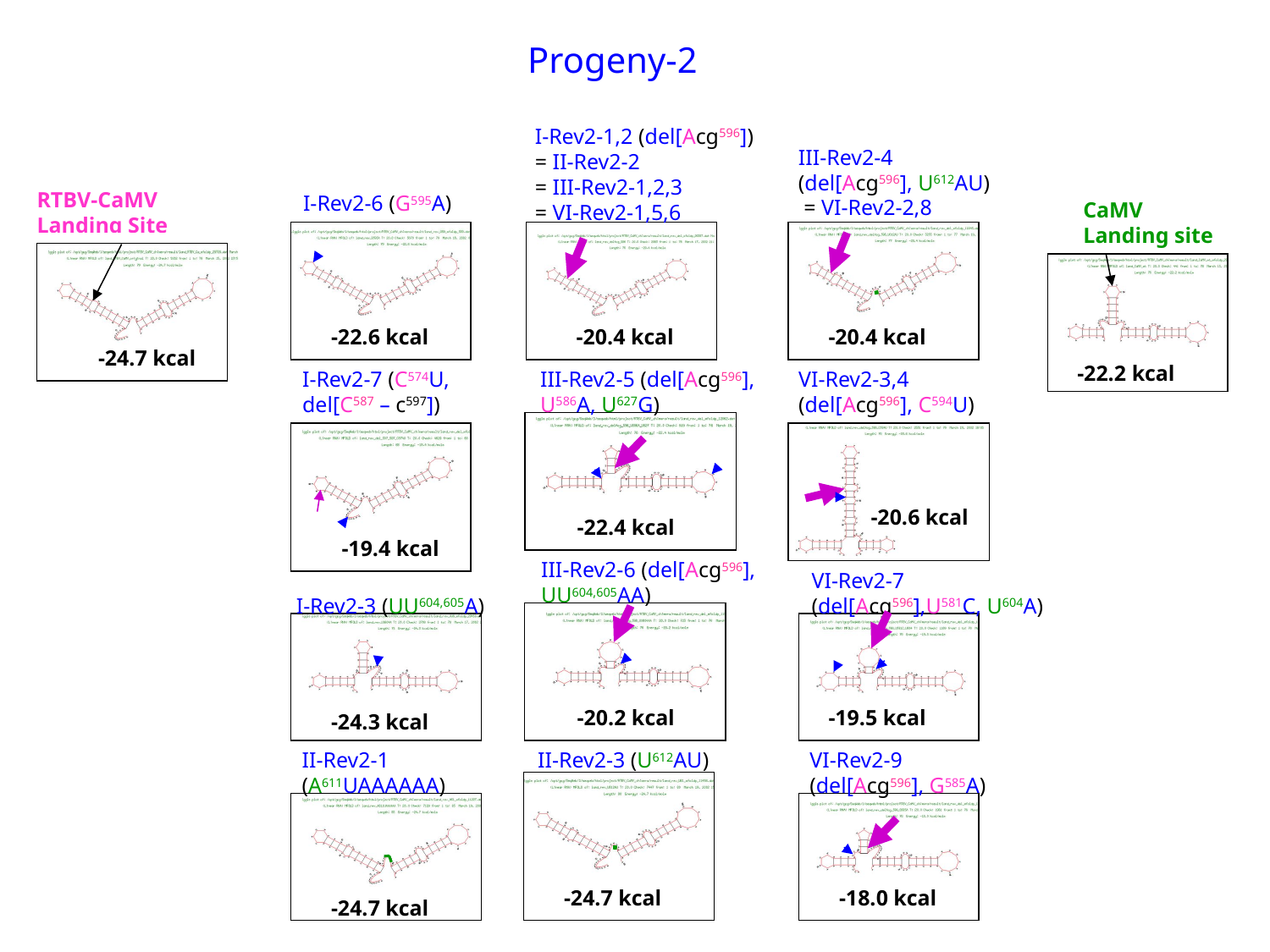

Progeny-2
I-Rev2-1,2 (del[Acg596])
= II-Rev2-2
= III-Rev2-1,2,3
= VI-Rev2-1,5,6
III-Rev2-4
(del[Acg596], U612AU)
 = VI-Rev2-2,8
RTBV-CaMV
Landing Site
I-Rev2-6 (G595A)
CaMV
Landing site
-22.6 kcal
-20.4 kcal
-20.4 kcal
-24.7 kcal
-22.2 kcal
I-Rev2-7 (C574U,
del[C587 – c597])
III-Rev2-5 (del[Acg596],
U586A, U627G)
VI-Rev2-3,4
(del[Acg596], C594U)
-20.6 kcal
-22.4 kcal
-19.4 kcal
III-Rev2-6 (del[Acg596],
UU604,605AA)
VI-Rev2-7
(del[Acg596],U581C, U604A)
I-Rev2-3 (UU604,605A)
-20.2 kcal
-19.5 kcal
-24.3 kcal
II-Rev2-1
(A611UAAAAAA)
II-Rev2-3 (U612AU)
VI-Rev2-9
(del[Acg596], G585A)
-24.7 kcal
-18.0 kcal
-24.7 kcal

## Slide 45
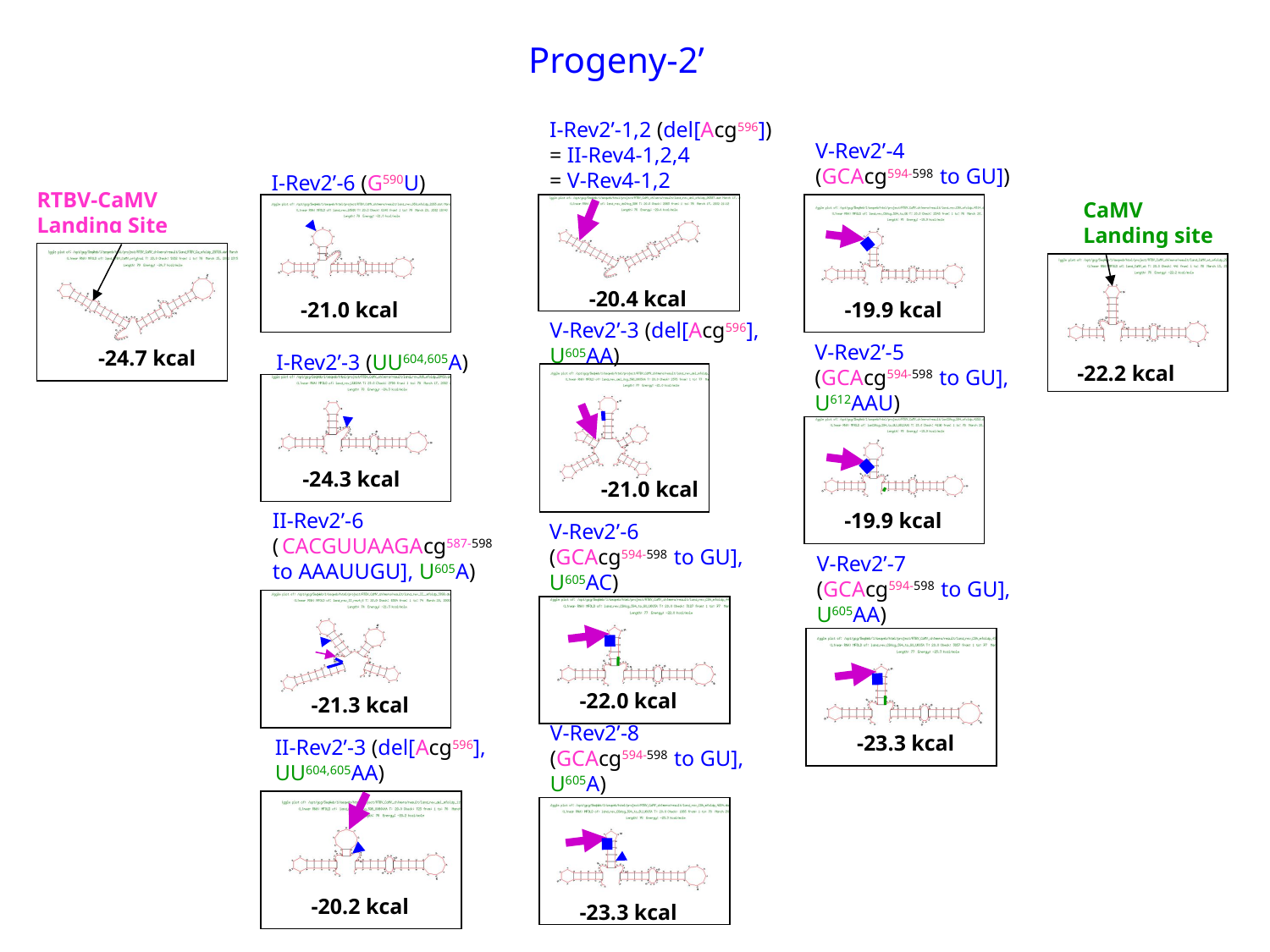

Progeny-2’
I-Rev2’-1,2 (del[Acg596])
= II-Rev4-1,2,4
= V-Rev4-1,2
V-Rev2’-4
(GCAcg594-598 to GU])
I-Rev2’-6 (G590U)
RTBV-CaMV
Landing Site
CaMV
Landing site
-20.4 kcal
-21.0 kcal
-19.9 kcal
V-Rev2’-3 (del[Acg596],
U605AA)
V-Rev2’-5
(GCAcg594-598 to GU],
U612AAU)
-24.7 kcal
I-Rev2’-3 (UU604,605A)
-22.2 kcal
-24.3 kcal
-21.0 kcal
II-Rev2’-6
( CACGUUAAGAcg587-598
to AAAUUGU], U605A)
-19.9 kcal
V-Rev2’-6
(GCAcg594-598 to GU],
U605AC)
V-Rev2’-7
(GCAcg594-598 to GU],
U605AA)
-22.0 kcal
-21.3 kcal
V-Rev2’-8
(GCAcg594-598 to GU],
U605A)
-23.3 kcal
II-Rev2’-3 (del[Acg596],
UU604,605AA)
-20.2 kcal
-23.3 kcal

## Slide 46
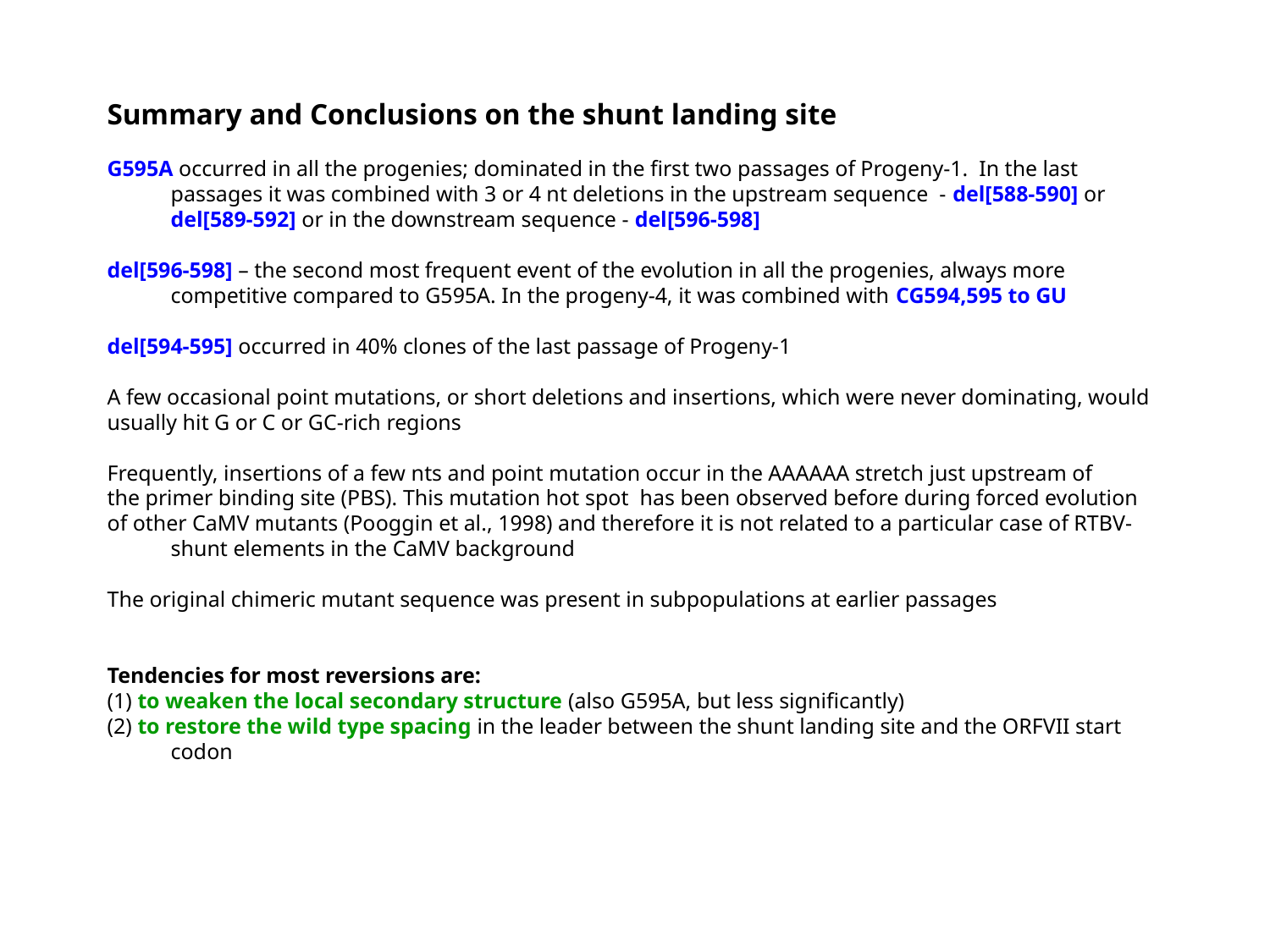

Summary and Conclusions on the shunt landing site
G595A occurred in all the progenies; dominated in the first two passages of Progeny-1. In the last passages it was combined with 3 or 4 nt deletions in the upstream sequence - del[588-590] or del[589-592] or in the downstream sequence - del[596-598]
del[596-598] – the second most frequent event of the evolution in all the progenies, always more competitive compared to G595A. In the progeny-4, it was combined with CG594,595 to GU
del[594-595] occurred in 40% clones of the last passage of Progeny-1
A few occasional point mutations, or short deletions and insertions, which were never dominating, would
usually hit G or C or GC-rich regions
Frequently, insertions of a few nts and point mutation occur in the AAAAAA stretch just upstream of
the primer binding site (PBS). This mutation hot spot has been observed before during forced evolution
of other CaMV mutants (Pooggin et al., 1998) and therefore it is not related to a particular case of RTBV-shunt elements in the CaMV background
The original chimeric mutant sequence was present in subpopulations at earlier passages
Tendencies for most reversions are:
(1) to weaken the local secondary structure (also G595A, but less significantly)
(2) to restore the wild type spacing in the leader between the shunt landing site and the ORFVII start codon

## Slide 47
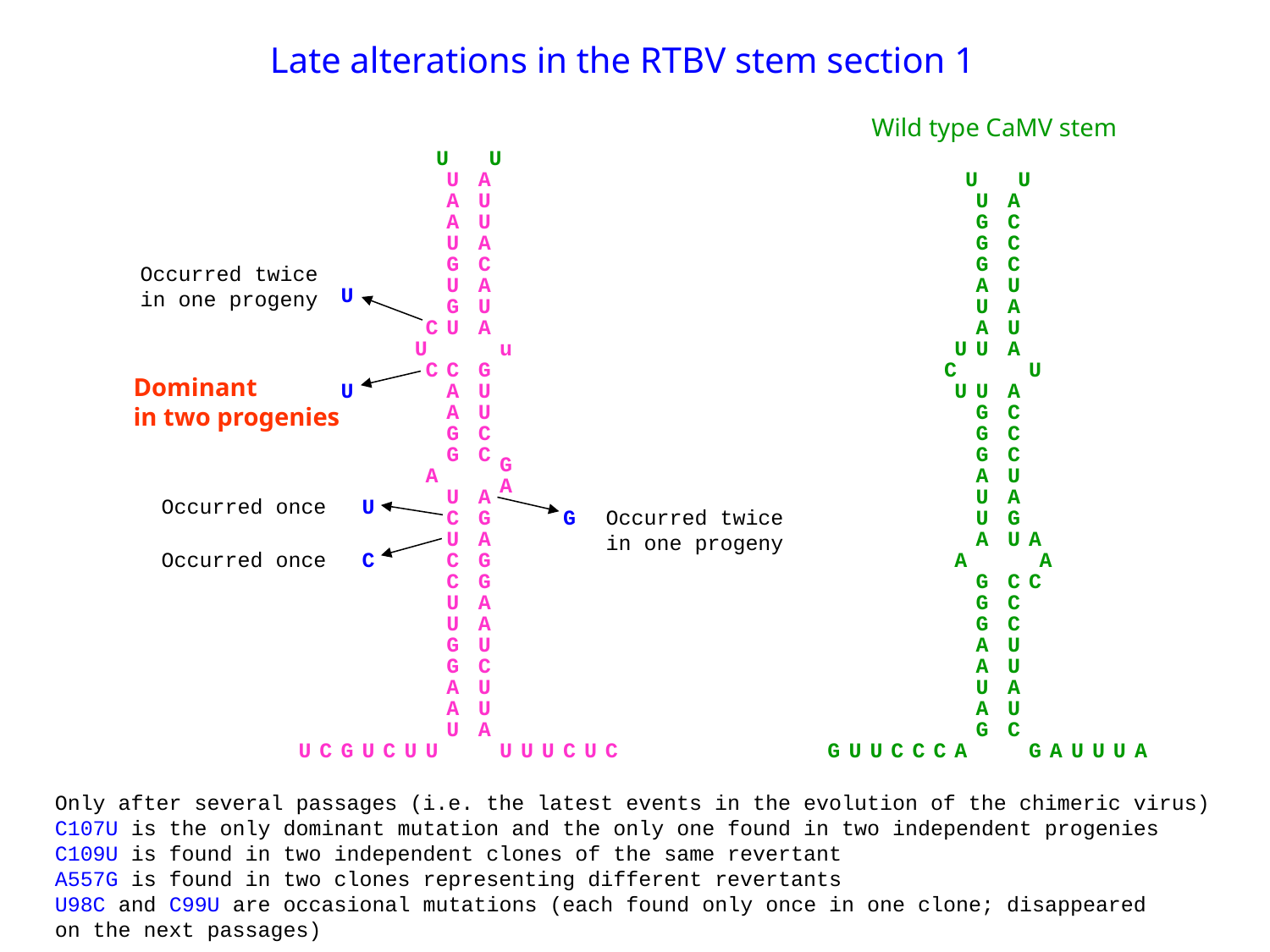

Late alterations in the RTBV stem section 1
Wild type CaMV stem
U
U
U
A
U
U
A
U
U
A
A
U
G
C
U
A
G
C
G
C
G
C
Occurred twice
in one progeny
U
A
A
U
U
G
U
U
A
C
U
A
A
U
U
u
U
U
A
C
C
G
C
U
Dominant
in two progenies
U
A
U
U
U
A
A
U
G
C
G
C
G
C
G
C
G
C
G
A
A
U
A
U
A
U
A
Occurred once
U
C
G
G
Occurred twice
in one progeny
U
G
U
A
A
U
A
Occurred once
C
C
G
A
A
C
G
G
C
C
U
A
G
C
U
A
G
C
G
U
A
U
G
C
A
U
A
U
U
A
A
U
A
U
U
A
G
C
U
C
G
U
C
U
U
U
U
U
C
U
C
G
U
U
C
C
C
A
G
A
U
U
U
A
Only after several passages (i.e. the latest events in the evolution of the chimeric virus)
C107U is the only dominant mutation and the only one found in two independent progenies
C109U is found in two independent clones of the same revertant
A557G is found in two clones representing different revertants
U98C and C99U are occasional mutations (each found only once in one clone; disappeared
on the next passages)

## Slide 48
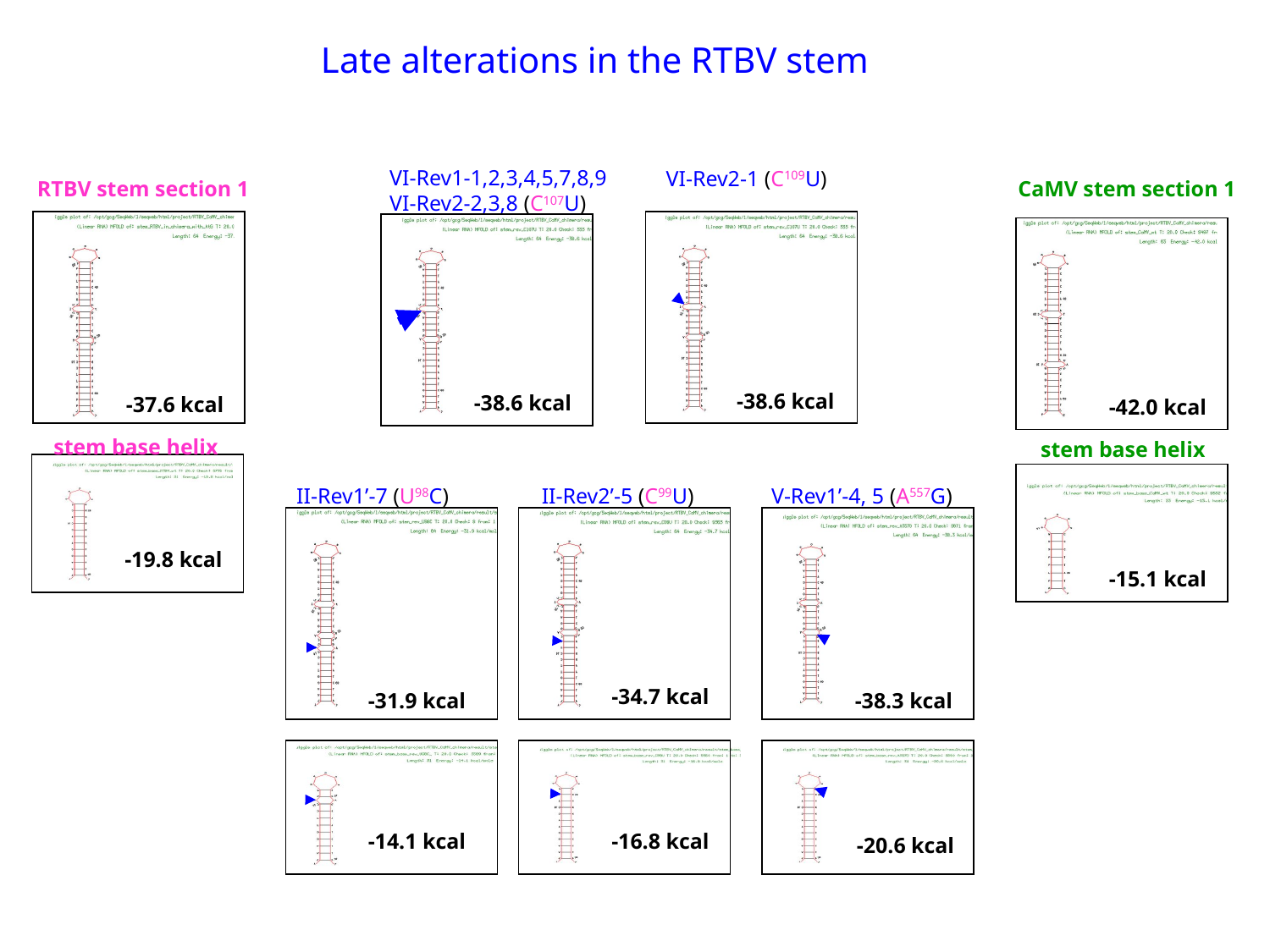

Late alterations in the RTBV stem
VI-Rev1-1,2,3,4,5,7,8,9
VI-Rev2-2,3,8 (C107U)
VI-Rev2-1 (C109U)
RTBV stem section 1
CaMV stem section 1
-38.6 kcal
-38.6 kcal
-37.6 kcal
-42.0 kcal
stem base helix
stem base helix
II-Rev1’-7 (U98C)
II-Rev2’-5 (C99U)
V-Rev1’-4, 5 (A557G)
-19.8 kcal
-15.1 kcal
-34.7 kcal
-31.9 kcal
-38.3 kcal
-14.1 kcal
-16.8 kcal
-20.6 kcal
